# Biological invasions: a global assessment of geographic distributions, long‐term trends, and data gaps

**DOI:** 10.1111/brv.70058

**Published:** 2025-08-12

**Authors:** Hanno Seebens, Laura A. Meyerson, David M. Richardson, Bernd Lenzner, Elena Tricarico, Franck Courchamp, Alla Aleksanyan, Emre Keskin, Hanieh Saeedi, Perpetra Akite, Jake M. Alexander, Sarah A. Bailey, Dino Biancolini, Tim M. Blackburn, Hans Juergen Boehmer, Alejandro Bortolus, Marc W. Cadotte, César Capinha, James T. Carlton, Jo Anne Crouch, Curtis C. Daehler, Franz Essl, Llewellyn C. Foxcroft, Jason D. Fridley, Nicol Fuentes, Mirijam Gaertner, Bella Galil, Emili García‐Berthou, Pablo García‐Díaz, Sylvia Haider, Liam Heneghan, Kevin A. Hughes, Cang Hui, Ekin Kaplan, Andrew M. Liebhold, Chunlong Liu, Elizabete Marchante, Hélia Marchante, Alicia Marticorena, David W. Minter, Rodrigo A. Moreno, Wolfgang Nentwig, Aidin Niamir, Ana Novoa, Ana L. Nunes, Aníbal Pauchard, Sebataolo Rahlao, Anthony Ricciardi, James C. Russell, K.V. Sankaran, Anna Schertler, Evangelina Schwindt, Ross T. Shackleton, Daniel Simberloff, David L. Strayer, Alifereti Tawake, Marco Thines, Cristóbal Villaseñor‐Parada, Jean Ricardo Simões Vitule, Viktoria Wagner, Victoria Werenkraut, Karsten Wesche, Demian A. Willette, Rafael D. Zenni, Petr Pyšek

**Affiliations:** ^1^ Department of Animal Ecology & Systematics Justus Liebig University Giessen Heinrich‐Buff‐Ring 26 Giessen 35392 Germany; ^2^ Senckenberg Biodiversity and Climate Research Centre Senckenberganlage 25 Frankfurt 60325 Germany; ^3^ Department of Natural Resources Science University of Rhode Island Woodward Hall, 9 Easy Alymnii Avenue Kingston RI 02881 USA; ^4^ Institute of Botany, Czech Academy of Sciences Zámek 1 Průhonice CZ‐25243 Czech Republic; ^5^ Centre for Invasion Biology, Department of Botany and Zoology Stellenbosch University Private Bag X1, Matieland Stellenbosch 7602 South Africa; ^6^ Division of BioInvasions, Macroecology and Global Change, Department of Botany and Biodiversity Research University Vienna Rennweg 14 1030 Vienna Austria; ^7^ Department of Biology University of Florence via Madonna del Piano 6 50019 Sesto Fiorentino (FI) Italy; ^8^ CNRS, AgroParisTech, Ecologie, Société & Evolution Université Paris Saclay Paris 91190 France; ^9^ Department of Geobotany and Ecophysiology Institute of Botany aft. A. Takhtajyan NAS RA Acharyan 1 0063 Yerevan Armenia; ^10^ International Scientific Educational Center of NAS RA Baghramyan 24d 0019 Yerevan Armenia; ^11^ Interdisciplinary Research Center of Armenian State University of Economics Nalbandyan 128 0025 Yerevan Armenia; ^12^ Evolutionary Genetics Laboratory (eGL), Department of Fisheries and Aquaculture Ankara University Agricultural Faculty Ankara 06135 Turkey; ^13^ Senckenberg Research Institute and Natural History Museum, Department of Marine Zoology, Biodiversity Information Section Senckenberganlage 25 60325 Frankfurt am Main Germany; ^14^ Goethe University Frankfurt, Department 15 ‐ Life Sciences Institute for Ecology, Evolution and Diversity Max‐von‐Laue‐Straße 13 60438 Frankfurt am Main Germany; ^15^ Department of Zoology, Entomology and Fisheries Sciences Makerere University Makerere Hill Road P.O Box 7062 Kampala Uganda; ^16^ Institute of Integrative Biology, ETH Zurich Universitätsstrasse 16 CH‐8092 Zurich Switzerland; ^17^ Great Lakes Laboratory for Fisheries and Aquatic Sciences, Fisheries and Oceans Canada 867 Lakeshore Road Burlington Ontario L7S 1A1 Canada; ^18^ National Research Council of Italy, Institute for BioEconomy (CNR‐IBE) Via dei Taurini Rome 19 00118 Italy; ^19^ IUCN SSC Invasive Species Specialist Group Viale Cesare Pavese Rome 305 00144 Italy; ^20^ Centre for Biodiversity and Environment Research, Department of Genetics, Evolution and Environment University College London London WC1E 6BT UK; ^21^ Institute of Zoology, Zoological Society of London London NW1 4RY UK; ^22^ Geobotany Section, Institute of Earth System Sciences, Leibniz University Hannover Nienburger Strasse 17 30167 Hannover Germany; ^23^ School of Geography, Earth Science & Environment, University of the South Pacific Suva Fiji; ^24^ Instituto Patagónico para el Estudio de los Ecosistemas Continentales (IPEEC‐CONICET) Puerto Madryn 9120 Chubut Argentina; ^25^ Department of Biological Sciences, University of Toronto Scarborough 1265 Military Trail Toronto Ontario M1C 1A4 Canada; ^26^ Centre of Geographical Studies, Institute of Geography and Spatial Planning, University of Lisbon R. Branca Edmée Marques Lisbon 1600‐276 Portugal; ^27^ Associate Laboratory Terra Tapada da Ajuda Lisbon 1349‐017 Portugal; ^28^ Coastal & Ocean Studies Program, Williams College‐Mystic Seaport 75 Greenmanville Ave Mystic CT 06355 USA; ^29^ United States Dept. of Agriculture, Agricultural Research Service, Foreign Disease/Weed Science Research Unit 1301 Ditto Avenu, Fort Detrick MD 21702 USA; ^30^ School of Life Sciences, University of Hawai'i at Mānoa Honolulu HI 96822 USA; ^31^ Scientific Services, South African National Parks Private Bag X402, Skukuza 1350 South Africa; ^32^ Department of Biological Sciences Clemson University 132 Long Hall Clemson South Carolina 29634 USA; ^33^ Departamento de Botánica, Facultad de Ciencias Naturales y Oceanográficas Universidad de Concepción Casilla 160‐C 4030000 Concepción Chile; ^34^ Nürtingen–Geislingen University Neckarsteige 6‐10 72622 Nürtingen Germany; ^35^ The Steinhardt Museum of Natural History, Israel National Center for Biodiversity Studies, Tel Aviv University Klausner 12 Tel Aviv 69978 Israel; ^36^ GRECO, Institute of Aquatic Ecology, University of Girona Maria Aurèlia Capmany i Farnés 69 Girona 17003 Spain; ^37^ School of Biological Sciences, Zoology building, University of Aberdeen Tillydrone Avenue Aberdeen AB24 2TZ UK; ^38^ Instituto de Ecología Regional (IER), Universidad Nacional de Tucumán (UNT) ‐ Consejo Nacional de Investigaciones Científicas y Técnicas (CONICET) 4107 Yerba Buena Tucumán Argentina; ^39^ Institute of Ecology, Leuphana University of Lüneburg Universitätsallee 1 Lüneburg 21335 Germany; ^40^ Department of Environmental Science and Studies DePaul University 1110 W. Belden Avenue Chicago IL 60614 USA; ^41^ British Antarctic Survey, Natural Environment Research Council High Cross, Madingley Road Cambridge CB30ET UK; ^42^ Centre for Invasion Biology, Department of Mathematical Sciences Stellenbosch University Stellenbosch 7602 South Africa; ^43^ National Institute for Theoretical and Computational Sciences, African Institute for Mathematical Sciences Cape Town 7945 South Africa; ^44^ Faculty of Forestry and Wood Sciences Czech University of Life Sciences Prague Praha 6 Suchdol Czech Republic; ^45^ USDA Forest Service Northern Research Station Morgantown WV 26505 USA; ^46^ The Key Laboratory of Mariculture, Ministry of Education College of Fisheries, Ocean University of China 5 Yushan Road Qingdao Shandong Province 266005 People's Republic of China; ^47^ Centre for Functional Ecology, Associate Laboratory TERRA, Department of Life Sciences University of Coimbra, Calçada Martim de Freitas Coimbra 3000‐456 Portugal; ^48^ Research Centre for Natural Resources Environment and Society (CERNAS), Polytechnic Institute of Coimbra, Coimbra Agriculture School Bencanta Coimbra 3045‐601 Portugal; ^49^ Herbario CONC, Departamento de Botánica, Facultad de Ciencias Naturales y Oceanográficas Universidad de Concepción Casilla 160‐C Concepción Chile; ^50^ CABI, Bakeham Lane, Egham Surrey TW20 9TY UK; ^51^ Fundación Tortumar‐Chile Serrano 1112 PC 1100606 Iquique Chile; ^52^ Institute of Ecology and Evolution, University of Bern Baltzerstrasse 6 Bern 3012 Switzerland; ^53^ Estación Experimental de Zonas Áridas (EEZA‐CSIC) Carretera Sacramento s/n Almería 04120 Spain; ^54^ IUCN, The David Attenborough Building Pembroke Street Cambridge CB2 3QZ UK; ^55^ Laboratorio de Invasiones Biológicas (LIB), Facultad de Ciencias Forestales Universidad de Concepción Victoria Concepción 631 Chile; ^56^ Institute of Ecology and Biodiversity (IEB) Victoria Concepción 631 Chile; ^57^ Oceanographic Research Institute 1 King Shaka Avenue Durban KwaZulu‐Natal South Africa; ^58^ School of Life Sciences, University of KwaZulu Natal Westville campus, University Road, Westville Durban 3629 South Africa; ^59^ Department of Biology McGill University 1205 Docteur Penfield, Montreal Quebec H3A 1B1 Canada; ^60^ School of Biological Sciences, University of Auckland Private Bag 92019 Auckland 1142 New Zealand; ^61^ Kerala Forest Research Institute Peechi 680 653 Kerala India; ^62^ Instituto de Biología de Organismos Marinos (IBIOMAR‐CONICET) Blvd. Brown 2915 Puerto Madryn 9120 Argentina; ^63^ Swiss Federal Institute for Forest, Snow and Landscape Research WSL Zürcherstrasse 111 Birmensdorf 8903 Switzerland; ^64^ Department of Ecology and Evolutionary Biology University of Tennessee Knoxville TN 37996 USA; ^65^ Cary Institute of Ecosystem Studies 1336 Glen Leven Road Ann Arbor MI 48103 USA; ^66^ Graham Sustainability Institute 625 E. Liberty Street, University of Michigan Ann Arbor MI 48104 USA; ^67^ Locally Managed Marine Area Network International Trust 41 Muktaben Place, Vatuwaqa Suva Fiji; ^68^ Goethe University Frankfurt Department of Biological Sciences, Institute of Ecology, Evolution and Diversity Max‐von‐Laue Str. 9 60438 Frankfurt am Main Germany; ^69^ Facultad de Ciencias Universidad Católica de la Santísima Concepción Alonso de Ribera 2850 4090541 Concepción Chile; ^70^ Laboratório de Ecologia e Conservação, Setor de Tecnologia, Departamento de Engenharia Ambiental Universidade Federal do Paraná Brazil; ^71^ Department of Biological Sciences University of Alberta T6G 2E Edmonton Alberta Canada; ^72^ Instituto de Investigaciones en Biodiversidad y Medio Ambiente, Consejo Nacional de investigaciones Científicas y Técnicas ‐ Universidad Nacional del Comahue Río Negro Argentina; ^73^ Senckenberg Museum for Natural History Görlitz Am Museum 1 02826 Görlitz Germany; ^74^ Biology Department Loyola Marymount University Los Angeles CA 90045 USA; ^75^ Departamento de Ecologia e Conservação Instituto de Ciências Naturais, Universidade Federal de Lavras Lavras, Trevo Rotatório Professor Edmir Sá Santos, s/n, Caixa Postal 3037 Lavras MG 37200‐090 Brazil; ^76^ Department of Ecology, Faculty of Science Charles University Viničná 7 Prague CZ‐12844 Czech Republic

**Keywords:** Neobiota, invasive species, non‐native, alien, biogeography, time series, worldwide, future projections, knowledge gaps, IPBES

## Abstract

Biological invasions are one of the major drivers of biodiversity decline and have been shown to have far‐reaching consequences for society and the economy. Preventing the introduction and spread of alien species represents the most effective solution to reducing their impacts on nature and human well‐being. However, implementing effective solutions requires a good understanding of where the species are established and how biological invasions develop over time. Knowledge of the status and trends of biological invasions is thus key for guiding research efforts, informing stakeholders and policymakers, for targeted management efforts, and preparing for the future. However, information about the status and trends of alien species is scattered, patchy, and highly incomplete, making it difficult to assess. Published reports for individual regions and taxonomic groups are available, but large‐scale overviews are scarce. A global assessment therefore requires a review of available knowledge with careful consideration of sampling and reporting biases. This paper provides a comprehensive global assessment of the status and trends of alien species for major taxonomic groups [Bacteria, Protozoa, Stramenopila, Alveolata, and Rhizaria (SAR), fungi, plants, and animals] for Intergovernmental Panel of Biodiversity and Ecosystem Services (IPBES) regions.

The review provides irrefutable evidence that alien species have been introduced to all regions worldwide including Antarctica and have spread to even the most remote islands. The numbers of alien species are increasing within all taxa and across all regions, and are often even accelerating. Large knowledge gaps exist, particularly for taxonomic groups other than vascular plants and vertebrates, for regions in Africa and Central Asia, and for aquatic realms. In fact, for inconspicuous species, such as Bacteria, Protozoa, and to some degree SAR and fungi, we found records for very few species and regions. Observed status and trends are thus highly influenced by research effort. More generally, it is likely that all lists for alien species of any taxonomic group and region are incomplete. The reported species numbers therefore represent minima, and we can expect additions to all lists in the near future. We identified six key challenges which need to be addressed to reduce knowledge gaps and to improve our ability to assess trends and status of biological invasions.

## I. INTRODUCTION

The introduction of alien and invasive alien species (see definitions in Section II below) can have far‐reaching consequences (Pyšek *et al*., 2020) for the distribution of life on Earth (Leroy *et al*., 2023; Aulus‐Giacosa, Ollier & Bertelsmeier, 2024), biodiversity conservation (Pyšek *et al*., 2012b) and native species survival (Blackburn, Bellard & Ricciardi, 2019; Bellard, Bernery & Leclerc, 2021), ecosystem functioning (Linders *et al*., 2019; Pérez, Vilà & Gallardo, 2022), human well‐being and plant and animal health (Lazzaro *et al*., 2018), and the economy (Diagne *et al*., 2021). The rate of new alien species introductions has risen continuously for centuries (Seebens *et al*., 2017) and is expected to keep rising (Seebens *et al*., 2021a). The ongoing accumulation and spread of alien species poses distinct challenges to assessing the current status of biological invasions. Comprehensive and regularly updated assessments of available knowledge about alien species distributions are essential for elucidating the underlying dynamics of biological invasions globally, for assessing current trends and data needs, and for informing and supporting managers and policy‐makers (Latombe *et al*., 2017; Meyerson *et al*., 2022).

The high dynamism of alien species introductions and spread complicates the task of providing up‐to‐date and accurate assessments of the status of biological invasions across the world. The establishment of new alien species is reported frequently worldwide (Seebens *et al*., 2021b). For example, the regularly updated alien flora of the Czech Republic reported an increase of the total number of alien plants from 1378 in 2002 to 1576 in 2022 (Pyšek *et al*., 2022). Fluctuations in alien species numbers result from a multitude of factors, such as the intensity of introductions of new species, environmental changes, or local disturbances (Pyšek *et al*., 2020). However, changes may also arise from the deployment of new technologies (Meyerson *et al*., 2022) and effective alien species management and eradication (Simberloff, 2011; Pluess *et al*., 2012a,b; Spatz *et al*., 2022). Further uncertainty arises from the highly variable intensity of research and the notorious inconsistency in reporting (Hughes *et al*., 2021; Meyerson *et al*., 2022). It is therefore necessary to conduct regular assessments of alien species' distributions, and their temporal trends, to integrate the most comprehensive available information, as well as taking data gaps into account.

Here, we present results of a comprehensive assessment of the historical trends and current distributions of alien species across geographic regions worldwide. This effort builds on the report of the Intergovernmental Panel of Biodiversity and Ecosystem Services of invasive alien species and their control (IPBES, 2023). This IPBES report presents a broad overview and assessment of the current situation of biological invasions, their impacts, management, and policy options. Chapter 2 of the report focuses on the trends and status of alien species (Seebens *et al*., 2023), which has been updated and revised here by assessing: (*i*) the levels of invasions by established alien Bacteria, Protozoa, SAR (monophyletic group consisting of Stramenopila, Alveolata, and Rhizaria; partly considered as Chromista), fungi, plants, and animals across all IPBES regions, both in terms of overall quantitative patterns and individual examples; (*ii*) the trends in the accumulation of established alien taxa and associated invasion dynamics that have resulted in their current distributions; and (*iii*) taxonomic and geographic data gaps, where future research efforts should be directed. We provide overviews of the trends and status of established alien species at the global scale, including an assessment of gaps (Section IV), for individual IPBES regions (Sections V., IX.), and future trends (Section X), followed by recommendations for improving the level of knowledge (Section XI).

## II. METHODS

We define key terms in this article as used in IPBES (2023) (Table 1). The information provided herein was collated in two ways: through comprehensive literature reviews and by generating a database of alien species occurrences to provide a quantitative basis for assessing the distribution of alien species.

**Table 1:** Definitions of key terms following IPBES (2023).

Key terms	Definitions
Trends	Temporal changes in established alien species numbers over decades to centuries.
Status	Currently observed and reported distribution patterns of alien species.
Gap	Absence of information on alien species occurrences, also called the Wallacean shortfall (Lomolino, 2004). A gap usually arises from low sampling effort, but might also be due to other reasons, such as the lack of accessibility of information, underreporting, unresolved taxonomies, or linguistic barriers.
Alien species	Species transported beyond the limits of their native range through human agency and that occur in the new region outside cultivation or captivity. We here refer to ‘established’ alien species, representing those alien species that form self‐sustaining populations in the wild (Richardson *et al*., 2000). This corresponds to category ‘C3’ in the framework proposed by Blackburn *et al*. (2011), and is a synonym of the term ‘naturalised’ (e.g. Richardson *et al*., 2000; Pyšek *et al*., 2017). For the sake of readability, we use ‘alien species’ in the main text although the numbers and examples refer to established alien species.
Invasive alien species	A subset of established alien species that spread and have a negative impact on biodiversity and local ecosystems following the definition of IPBES (2023) and IUCN (2000). We note that neither spread nor negative impact have agreed‐upon quantitative definitions.

The database underlying this article represents a collection of standardised checklists of alien species for regions worldwide and has been generated by applying a published workflow called ‘Standardisation and Integration of Alien Species distribution data’ (SInAS) (Seebens *et al*., 2020). This workflow standardises information available in checklists (i.e. lists of species per region) and integrates data into a single combined database. The R code for applying the workflow has been published previously (Seebens *et al*., 2020); the generation of the final database is thus fully transparent and reproducible. Standardisation included the harmonisation of taxonomic names according to the backbone taxonomy of the Global Biodiversity Information Facility (GBIF, www.gbif.org), the harmonisation of key terms according to Groom *et al*. (2019), matching the spatial resolution of all checklists, and the standardisation of years of first records according to rules defined in the workflow and outlined in Seebens *et al*. (2020). Once all the data were standardised, the checklists were combined, and duplicates removed. Conflicting entries from different databases that could not be resolved were retained and are flagged as such in the database.

To ensure transparency and achieve comprehensiveness, we sought published and freely accessible global databases of alien species checklists. Seven databases (Table 2) met these criteria; these together collate more than 4000 individual sources of information including scientific publications, reports, and regional databases. Integrating these sources resulted in the single database underlying this article that is called ‘SInAS database 2.5’. The integration achieved by applying the SInAS workflow to the seven databases resulted in the largest currently available single database of alien species, with 175,980 occurrences in 264 regions of 37,591 established alien species worldwide. The database is freely available (https://doi.org/10.5281/zenodo.10038256) and follows the data principles of being findable, accessible, interoperable, and reusable (FAIR).

**Table 2:** Databases of alien species used for generating the database underlying the figures and tables herein. In these databases, regions correspond to countries, administrative units in case of large countries, and islands, and a ‘regional record’ denotes a record of an established alien species in such a region.

Database	Content used here	Citation and source
Global Naturalised Alien Flora (GloNAF)	Regional records of alien vascular plants	van Kleunen *et al*. (2019) https://idata.idiv.de/DDM/Data/ShowData/257
Global Avian Invasions Atlas (GAVIA)	Regional records of alien birds	Dyer *et al*. (2017b) https://doi.org/10.1038/sdata.2017.41
Distribution of Alien Mammals (DAMA)	Regional records of alien mammals	Biancolini *et al*. (2021) https://doi.org/10.6084/m9.figshare.13014368
Alien amphibians and reptiles	Regional records of alien amphibians and reptiles	Capinha *et al*. (2017) https://doi.org/10.1111/ddi.12617
MacroFungi	Regional records of alien macrofungi	Monteiro *et al*. (2020) https://doi.org/10.15468/2qky1q
Alien Species First Records (FirstRecords)	First records of alien species in regions across taxonomic groups	Seebens *et al*. (2017) https://doi.org/10.5281/zenodo.4632335
GRIIS	Regional records of alien and invasive alien species across taxonomic groups	Pagad *et al*. (2022) https://doi.org/10.5281/zenodo.6348164

As the spatial delineation of the study, we adopted the classification of IPBES regions and subregions (IPBES, 2021), as used in their recent biodiversity reports (IPBES, 2019, 2023). This classification recognises five IPBES regions (‘Africa’, ‘Asia & the Pacific’, ‘The Americas’, ‘Europe & Central Asia’, and ‘Antarctica’) and 18 subregions (3–5 subregions for each region except Antarctica, which is not subdivided). We do not cover Antarctica in the same detail as the other IPBES regions as it has far fewer reported alien species. We used the IPBES classification for providing overviews in the text and tables, while the maps shown in the main text keep the regional and national categorisation of the underlying databases.

To provide overviews of status and trends, we grouped taxa according to their taxonomic classification following the GBIF backbone taxonomy with two exceptions: We included the group algae, which is a paraphyletic group including red algae (Rhodophyta), green algae (Chlorophyta, Charophyta), cryptophytes (Chryptophyta), and haptophytes (Haptophyta). The GBIF taxonomy includes the kingdom Chromista, which is an outdated classification. Instead, we used the supergroup SAR, which is a monophyletic group consisting of Stramenopila, Alveolata, and Rhizaria.

## IV. A GLOBAL OVERVIEW

### (1) Historical trends and current status

**Fig. 1:**
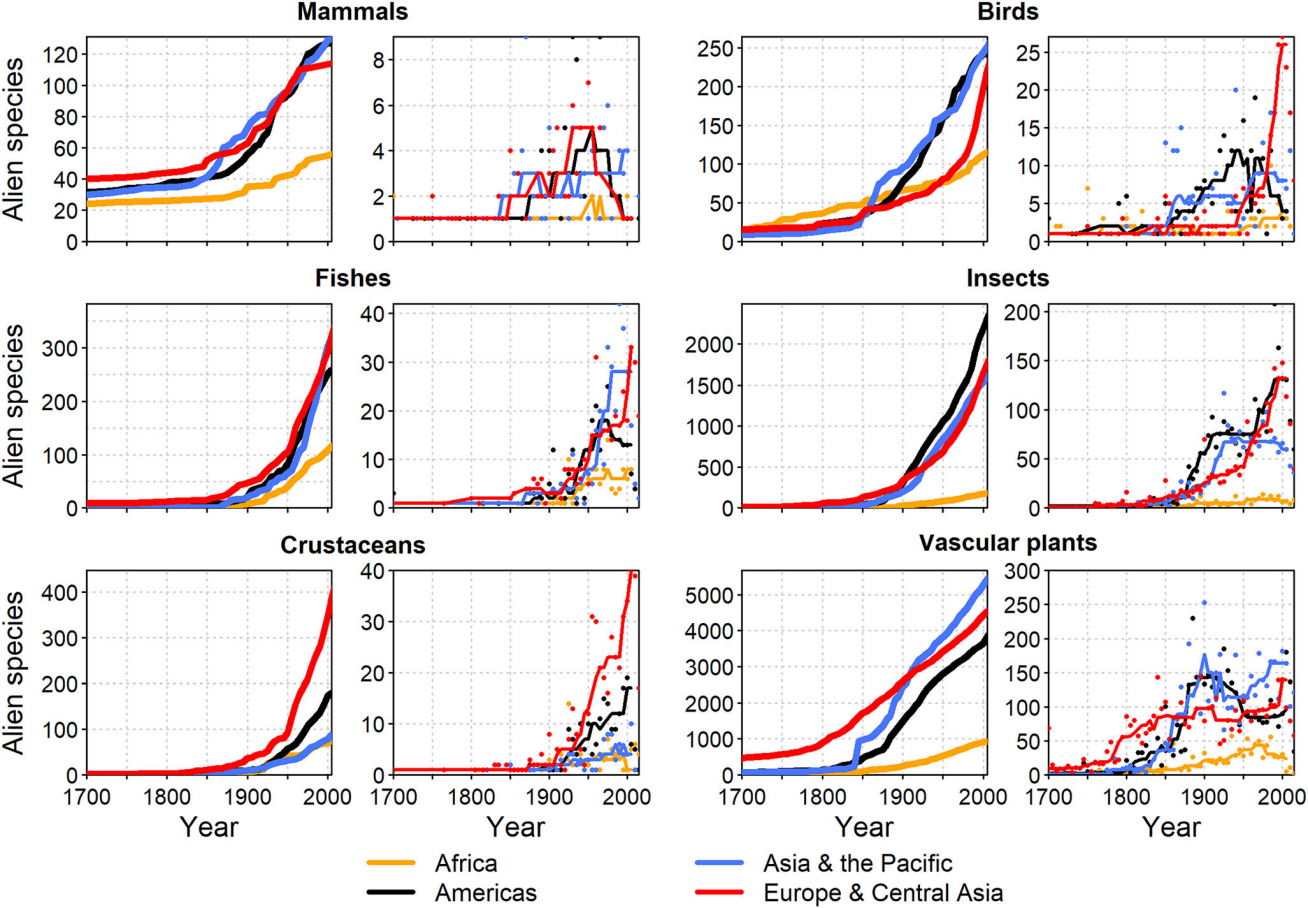
Trends in numbers of established alien species for Intergovernmental Panel of Biodiversity and Ecosystem Services (IPBES) world regions. Panels by taxon show cumulative numbers (left panels, thick lines) and numbers of new alien species per five‐year intervals (right panels, thin lines). Lines in right panels indicate smoothed trends calculated as running medians. Note that the range of the *y*‐axes differs among panels.

The number of alien species records has increased since 1700 CE consistently across all taxonomic groups and regions (Fig. 1). Between 1700 and 1850, the number of alien species was comparatively low and increased only slightly for all taxa. Numbers of alien species records rose markedly during the 19th century, and that trend continues today. The onset of the acceleration in alien species numbers varied slightly among taxonomic groups with a tendency of earlier onsets for mammals, birds, and vascular plants, and a later acceleration for invertebrates (Fig. 1). However, this may partly be an effect of lower research intensity and data availability for the latter group, which resulted in more delayed detections (Seebens *et al*., 2017; Muñoz‐Mas *et al*., 2023). The time series of alien species numbers were very similar for all IPBES regions except Africa, which shows lower values, perhaps in part due to lower data availability. In addition to cumulative numbers, the rates of newly recorded alien species have risen continuously for all taxonomic groups and nearly all regions except for mammals, which peaked around 1950 (Fig. 1). The declining trend in rates of alien mammal introductions is likely due to more stringent regulation of trade, their higher detectability compared to smaller organisms, the comparatively small pool of potential candidate species, and some successful eradications (Simberloff *et al*., 2013; Seebens *et al*., 2017).

**Fig. 2:**
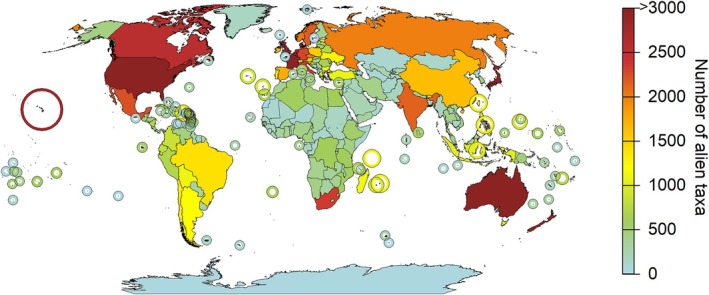
Numbers of established alien species per region. Species from all realms (marine, freshwater, and terrestrial) were considered if they could be assigned to one of the regions used in this study. Species only recorded for the open sea are not included in this map. Note that numbers may deviate from those reported in the text due to variation among data sources.

Currently observed numbers of alien species vary across IPBES regions, with the highest numbers recorded for Europe & Central Asia followed by The Americas, Asia & the Pacific, and Africa (Fig. 2, Table 3). Countries with particularly high numbers of alien species include the USA and Australia, which are also geographically large areas, and New Zealand, France, and the UK. Hawaii stands out as being a group of remote islands with invasion levels comparable to countries, such as Japan or Belgium, which are distinctly larger in area.

**Table 3:** Numbers of established alien species for the Intergovernmental Panel of Biodiversity and Ecosystem Services (IPBES) regions. The numbers are extracted from the SInAS database (see Section II) and may deviate from those reported in regional studies. The numbers should be considered as minimum values as the true level of invasion is likely higher. For mammals, birds, and vascular plants, ranges of values indicate variation among databases. Duplicated taxa for the same region and Totals were removed. SAR, Stramenopila, Alveolata, Rhizaria.

	Africa	The Americas	Asia & the Pacific	Europe & Central Asia	Totals
Mammals	30–80	83–164	97–163	72–164	197–368
Birds	121–133	249–287	287–336	221–630	495–877
Fishes	187	803	633	469	1451
Reptiles	158	192	103	98	411
Amphibians	12	62	43	43	135
Insects	344	2636	2017	2747	6795
Arachnids	94	207	129	289	500
Molluscs	142	255	261	584	826
Crustaceans	125	248	158	563	813
Vascular plants	3109–4498	8005–9325	6141–9101	5146–8519	13,081–18,543
Algae	40	55	66	212	270
Bryophytes		61	44	37	118
Fungi	122	363	363	609	1149
SAR	22	150	103	373	534
Bacteria and protozoans	4	14	12	23	38
Totals	4510 – 5961	13,383–14,822	10,457–13,532	11,486–15,360	26,813–32,828

Despite the larger area, Asia & the Pacific harbour similar numbers of alien amphibians and reptiles to Europe & Central Asia (Table 3), which is possibly a result of stringent biosecurity measures in some areas such as Australia, New Zealand, and Japan (Brenton‐Rule, Barbieri & Lester, 2016; Chapple *et al*., 2016; García‐Díaz *et al*., 2017; Toomes *et al*., 2020). Uneven sampling and reporting likely affect the reported total numbers of alien taxa, particularly in Africa, South America, Central Asia & the Pacific (Henriksen *et al*., 2024). In addition, articles not written in English and grey literature were more difficult for us to include in our analyses, resulting in a bias towards English sources.

Using comprehensive and well‐curated taxon‐specific databases (Table 2), we calculated the range of species numbers for mammals, birds, and vascular plants in the individual taxon‐specific databases compared to the full database (Table 3). The differences in numbers are high with nearly 30% more species reported in the full database compared to the taxon‐specific ones. The difference among databases is likely an effect of using different criteria for adding species by, for example, selecting information that is more or less robust. Thus, absolute numbers of species should be treated with caution and considered as estimates and often minimum numbers of true alien species levels.

Across taxonomic groups, vascular plants are by far the largest contributors to global alien species numbers, followed by insects and fishes (Table 3). For many taxonomic groups, all regions except Africa report similar numbers of established alien species. For instance, the numbers of vascular plant species reported for The Americas, Asia & the Pacific, and Europe & Central Asia are similar, while the number for Africa is much lower. Similar patterns are observed for alien bird, fish, and mammal species. By contrast, algae show a different pattern, with Europe & Central Asia harbouring the highest alien species numbers, followed by Asia & the Pacific, The Americas, and Africa. However, some of the observed patterns are certainly influenced by variation in survey intensity and availability of information around the world.

### (2) Biases and information availability

**Fig. 3:**
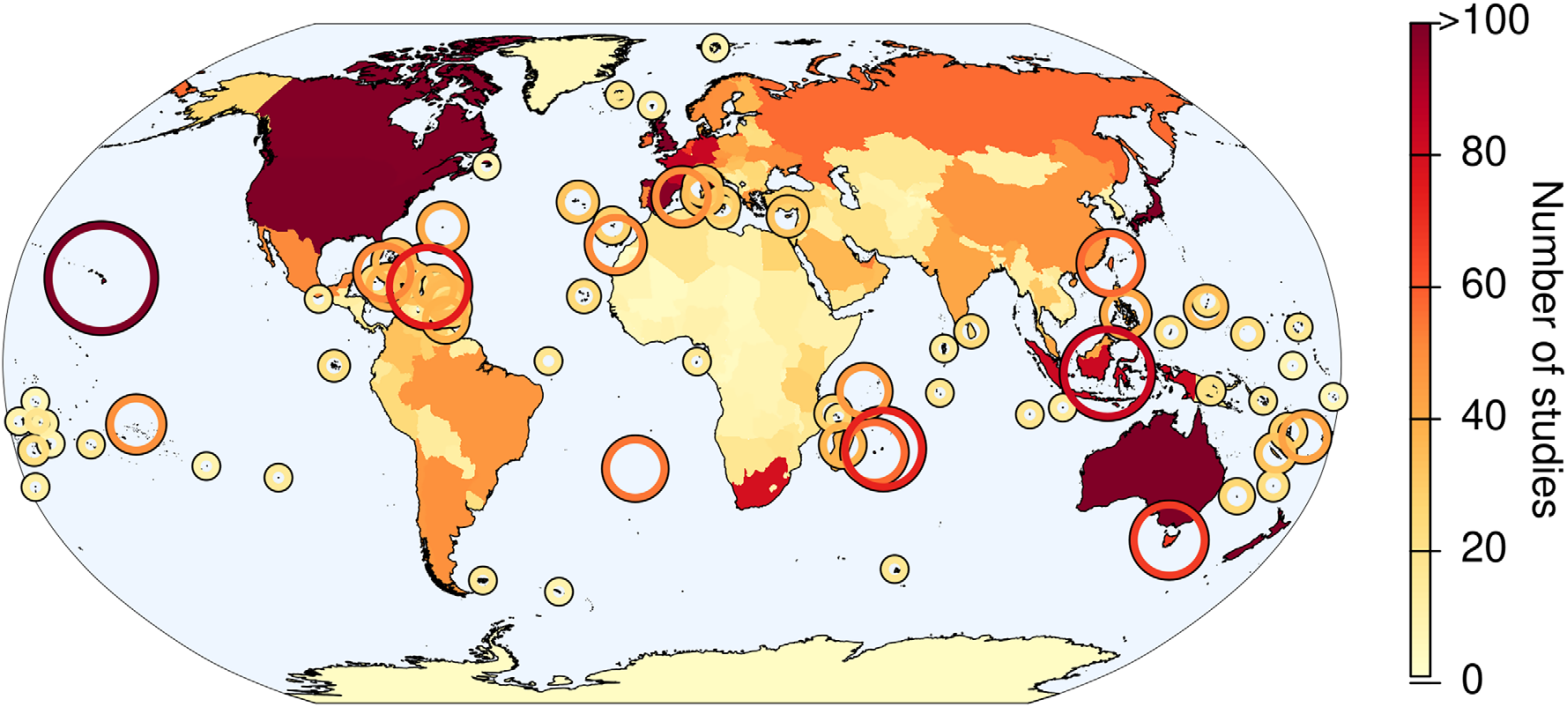
Research intensity and data gaps for global alien species distribution records. Research intensity is indicated by the number of studies available for individual regions as listed in the database of this study. Islands are indicated by circles. Circle sizes increase with increasing numbers of studies.

Patterns of the distribution of alien species are influenced by the uneven sampling of alien species occurrences across the globe and the uneven availability of information. For example, hotspots of alien species occurrences (i.e. areas of high alien species numbers relative to other regions; Dawson *et al*., 2017) coincide with global hotspots of data availability and study sites (Martin, Blossey & Ellis, 2012; Meyer *et al*., 2015), which influence our knowledge of species distributions (Hughes *et al*., 2021). This conclusion is confirmed by the information provided here: mapping the number of available studies used to generate the underlying database of this study revealed that regions with the most information on alien species occurrences (Fig. 3) were also the hotspots of alien species occurrences (Fig. 2). Hence, knowledge of alien species occurrences is biased towards well‐sampled regions, such as Europe and North America, and taxonomic groups, such as vertebrates and plants, with most studies conducted in recent decades (Pyšek *et al*., 2008; Jeschke *et al*., 2012; Bellard & Jeschke, 2016). It remains unclear how much the distribution of alien species and documented hotspots are affected by spatial variation in research intensity and data availability, and how much represents the ‘true’ distributional pattern of alien species. Such biases need to be taken into consideration when assessing the trends and status of alien species (McGeoch *et al*., 2023).

There are conspicuous areas of low research intensity (i.e. fewer studies), particularly over large parts of Africa, Asia, and Antarctica (Fig. 3). Some islands or archipelagos have been thoroughly studied, while information is scarce for many small and remote islands. In addition to regional biases, research intensity varies across taxonomic groups. Considerably more information is available on the distribution of alien vertebrates, particularly mammals and birds, and vascular plants than for other taxa. The reasons for these knowledge gaps include undersampling, underdeveloped taxonomies, incomplete knowledge about invasion status and distributions of alien species (Hughes *et al*., 2021; Carlton & Schwindt, 2024). Very little information is available for alien microorganisms or small invertebrates (e.g. Annelida, Porifera, or Nematoda) worldwide and recorded distributions often reflect the availability of studies rather than true species distributions. Moreover, even when such information is available, it is often highly incomplete.

## V. AFRICA

### (1) Bacteria and Protozoa

Among alien prokaryotes and protozoans, most records represent agents of infectious diseases either in humans or plants. *Vibrio cholerae* (cholera) is a famous example of alien Bacteria and one of the few prokaryotes with good distributional data. This species, originating from South India, was introduced to Africa in the early 19th century and is considered established in several African countries. As another example, the plant pathogen *Erwinia amylovora* (fire blight) was reported for Egypt in the 1960s and other mostly North African countries (Vanneste, 2008). No records of alien protozoans exist for Africa in the SInAS database.

### (2) SAR

Altogether, 22 species of SAR have been reported in the SInAS database (Table 4), including eight species from South Africa, five from Egypt, and three from Libya. A majority of these are aquatic species, such as Phaeophyceae (brown algae), Tubothalamea (Foraminifera), and Dinophyceae (dinoflagellates). The ciliate *Mirofolliculina limnoriae* is reported as being alien for South Africa (Mead *et al*., 2011) and two species of the genus *Sargassum* (brown algae) were found in Sierra Leone (Norman *et al*., 2021). First records are often missing, but two of the earliest records of alien SAR in Africa stem from Libya with *Padina boryana* (brown alga), reported in 1974 (Zenetos *et al*., 2022) and the dinoflagellate *Dinophysis acuminata* in 1991 in South Africa (Mead *et al*., 2011). Several oomycete species, especially of the genus *Phytophthora*, have been recorded across Africa. For example, *P. infestans* (potato blight) was reported first in 1941 in Kenya, from where it spread to other African countries (Njoroge *et al*., 2019). Other species of that genus widespread in Africa are *P. cinnamomi* (root rot), *P. nicotianae* (black shank), and *P. palmivora* (coconut budrot) (Barwell *et al*., 2020). *Plasmopara viticola*, the downy mildew of grapevine, was introduced from North America to other wine‐producing regions of the world and recorded in South Africa for the first time in 1907 (Koopman *et al*., 2007).

**Table 4:** Numbers of established alien species for subregions of Africa. The numbers are extracted from the SInAS database (see Section II) and may deviate from those reported in regional studies. The numbers should be considered as minimum values as the true level of invasion is likely much higher. For mammals, birds, and vascular plants, ranges of values indicate variation among databases. Duplicated taxa for the same region and Totals were removed. SAR, Stramenopila, Alveolata, Rhizaria.

	Central Africa	East Africa and adjacent islands	North Africa	Southern Africa	West Africa	Totals
Mammals	4–17	17–35	5–17	9–54	1–9	30–80
Birds	13–16	77–79	17–20	71–74	14–23	121–133
Fishes	26	56	130	46	17	187
Reptiles	2	33	8	124	9	158
Amphibians		5	2	2	5	12
Insects	33	143	71	227	48	344
Arachnids	9	29	10	70	11	94
Molluscs	2	11	75	67	7	142
Crustaceans	1	11	82	47	3	125
Vascular plants	880–1071	1738–2570	485–1162	1754–2292	645–818	3109–4498
Algae	1	4	30	7	1	40
Bryophytes						
Fungi	19	44	18	82	9	122
SAR	2	1	12	8		22
Bacteria and protozoans	1	2	1	2	1	4
Totals	993–1200	2171–3023	946–1638	2516–3102	771–961	4510–5961

### (3) Fungi

A comparatively low number of 122 alien fungal species is available for Africa according to the SInAS database, while true numbers are likely much higher (Table 4). One of the earliest reported introductions to Africa was *Armillaria mellea* (honey fungus) in South Africa (Cape Town region), which presumably was introduced in the early 17th century by European settlers (Coetzee *et al*., 2001). The plant pathogen *Claviceps africana* (ergot) was first reported in Kenya in 1924 and spread particularly in the 1980s, since when it has been recorded from several sub‐Saharan countries from Ghana to Lesotho (CABI, 2009). *Batrachochytrium dendrobatidis* (chytrid fungus), likely originating from Asia, has caused amphibian declines worldwide, including in Africa (Scheele *et al*., 2019). The trend of recording new alien fungi increased until the 1980s, while new detections of alien fungal plant pathogens in Africa have been declining more recently relative to reported increases in other regions of the world (Waage *et al*., 2008). This is likely a consequence of low research effort rather than true decline, because new records of alien fungi are increasing worldwide (Seebens *et al*., 2017; Sandvik *et al*., 2019; Fuentes *et al*., 2020).

In South Africa, nine alien fungal species are known to infect native plants, while 23 host‐specific fungi of alien plant species have likely been introduced together with their hosts (Wood, 2017). In addition, 11 alien saprotrophic species, and 61 species of alien fungi forming ectomycorrhizae have been reported (Wood, 2017). Furthermore, seven host‐specific alien pathogens have been introduced for the biological control of invasive alien plants (Wood, 2017). *Ceratocystis fimbriata* (ceratocystis blight) was found as an alien plant pathogen in several countries of Central and Southern Africa (CABI, 2022).

Compared to other regions of the world, Africa, with 107 species, has the lowest number of known alien macrofungi (Monteiro *et al*., 2020), possibly because of lower research intensity. Of these, 40% of species belong to Agaricales, 29% to Boletales, and 13% to Russulales. The most widespread macrofungal species are *Pyrrhoderma noxium*, *Amanita muscaria* (fly agaric), *Pisolithus albus* (white dye‐ball fungus), *Rhizopogon luteolus* (yellow false truffle), and *Suillus granulatus* (weeping bolete mushroom), recorded in eight or more countries. The highest numbers of alien macrofungi are reported for South Africa (65), Tanzania (25), Morocco (10), and Kenya (10).

### (4) Plantae

#### (a) Historical trends

**Fig. 4:**
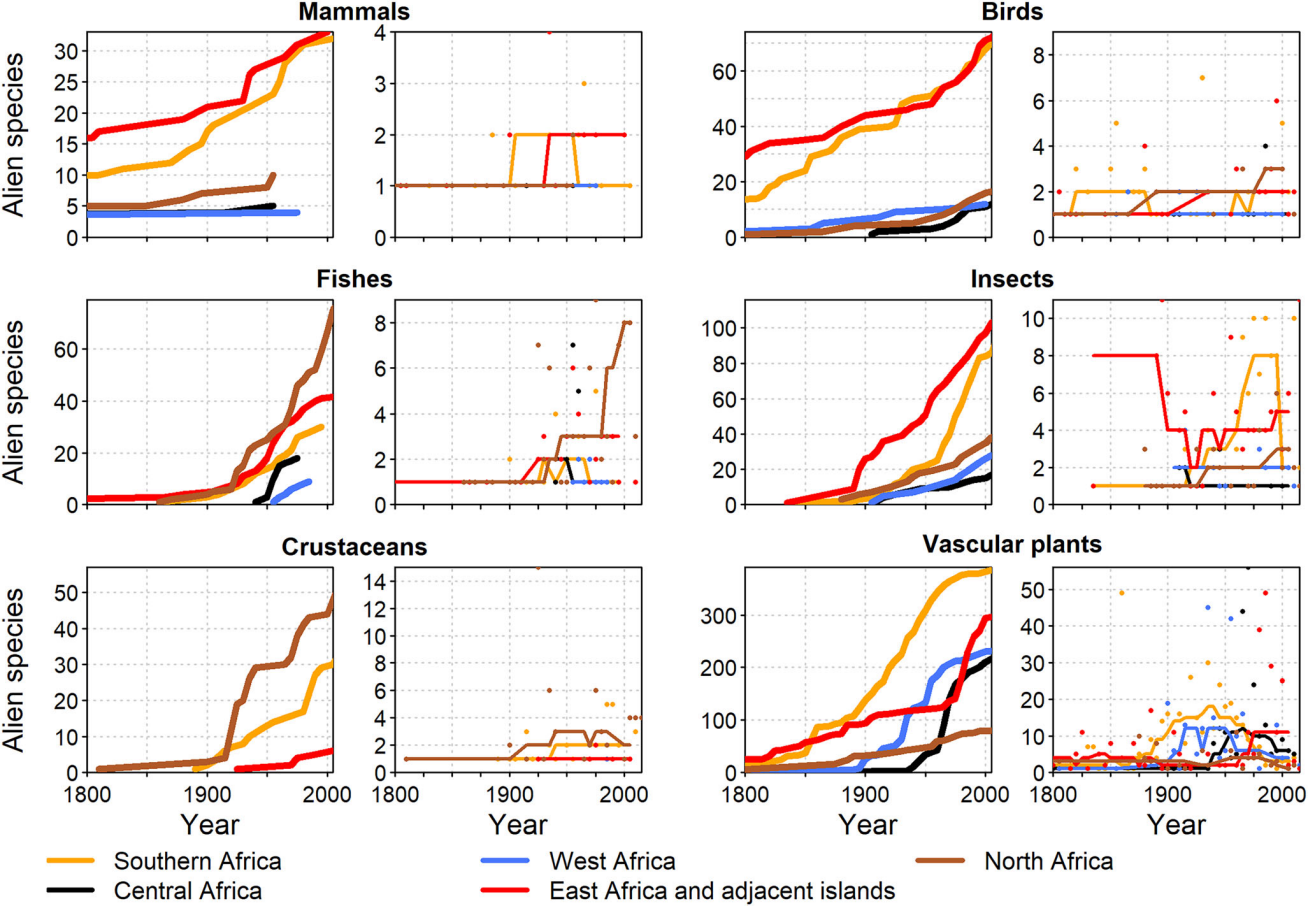
Trends in numbers of established alien species for Africa. Panels by taxon show cumulative numbers (left panels, thick lines) and numbers of new alien species per five‐year intervals (right panels, thin lines). The actual numbers of alien species occurrences are underestimated due to a lack of data. Lines in right panels indicate smoothed trends calculated as running medians. Note that presented numbers may deviate from those reported in the text due to variation among data sources.

The number of alien plant species in Africa has increased continually for centuries as reported for multiple African countries (Henderson, 2006; Maroyi, 2012; Senan *et al*., 2012; Brundu & Camarda, 2013; Shaltout *et al*., 2016). Southern Africa experienced a steady increase in numbers of alien plant species during the entire 19th century, which was the most rapid rise of all African regions; this trend appeared to slow down only towards the end of that century (Fig. 4). By contrast, alien plant numbers in East Africa showed a marked acceleration starting in the final quarter of the 20th century and have not yet slowed down, whereas in North Africa, the numbers of alien plants increased slowly but steadily towards the end of the 19th century. No readily apparent trends could be detected for West Africa. However, trends are certainly affected by data availability and research intensity (Pyšek *et al*., 2008, 2020; Richardson *et al*., 2022).

#### (b) Current status

For most of Africa, very little information is available on the status of alien plants. Although lists of alien plant species have been published for Algeria, Angola, Eswatini, Chad, Ghana, Lesotho, Madagascar, Namibia, and Zimbabwe (many in the last decade; references in Richardson *et al*., 2022), only for South Africa is detailed information of the status of species available – with 759 alien plant species (Richardson *et al*., 2020). The limited data for the rest of the continent summarised in Richardson *et al*. (2022) suggest that seven other countries harbour over 300 alien plant species: Congo (522), Ethiopia (421), Morocco (410), Mozambique (396), Benin (333), Algeria (328), and Eswatini (315). South Africa also has the highest number of invasive alien species (374). Species that are widely distributed over large parts of the continent include *Lantana camara* (lantana), *Tithonia diversifolia* (Mexican sunflower), *Pontederia crassipes* (water hyacinth), *Chromolaena odorata* (Siam weed), *Leucaena leucocephala* (leucaena), *Prosopis juliflora* (mesquite), and *Parthenium hysterophorus* (parthenium weed) (Richardson *et al*., 2022).

Many tree species used in forestry and agroforestry, especially from the genera *Acacia*, *Eucalyptus*, and *Pinus*, have been introduced throughout Africa, and some shrubs and trees, such as *Acacia melanoxylon* (Australian blackwood), *Broussonetia papyrifera* (paper mulberry), and *Calliandra houstoniana* (calliandra), are well established in many parts of the continent (Richardson *et al*., 2022). Australian *Acacia* species are actively promoted for agroforestry in several parts of the continent (Richardson, Binggeli & Botella, 2023) and areas at higher altitudes have been heavily invaded by *A. melanoxylon*, *A. mearnsii* (black wattle), *Pinus patula* (Mexican weeping pine), and *P. radiata* (radiata pine). Pines are particularly invasive in the Southwestern mountains of South Africa and *Acacia* species invade many ecosystems in the country (Holmes *et al*., 2005). *Prosopis juliflora* (mesquite) is common in semi‐arid and arid areas of Southern and Eastern Africa. Other tree and shrub invaders with impacts include several *Rubus* (bramble) species and *Biancaea decapetala* (Mysore thorn). *Azadirachta indica* (neem tree), *Lantana camara* (lantana), and *Leucaena leucocephala* (leucaena) are abundant invaders along the coastline of much of Africa, preferring hot and humid conditions. *Chromolaena odorata* (Siam weed) is now common in many countries in Central and Southern Africa, being abundant in open savanna grasslands, woodlands, riparian zones, forest gaps, and edges (Richardson *et al*., 2022).

La Réunion is estimated to have over 2000 alien plant species, with more than 100 of these classified as invasive, e.g. *Leucaena leucocephala* (leucaena), *Hiptage benghalensis* (hiptage), and *Ulex europaeus* (gorse) (Baret *et al*., 2006; Soubeyran *et al*., 2015). On Socotra, 88 alien plants have been recorded (Senan *et al*., 2012). A total of 28 major alien aquatic plants has been recorded in African waters, 16 of which are alien to the whole of Africa, and 12 are native to other parts of the continent (Howard & Chege, 2007; Sghaier *et al*., 2011). A recent review records the existence of 19 alien freshwater plants only in South Africa, mainly introduced through trade and hitchhiking *via* boating and angling (Hill *et al*., 2020). In South Africa, the most important alien freshwater macrophyte remains *Pontederia crassipes* (water hyacinth). The marine alien seagrass *Halophila stipulacea* is native to the East African coast, yet is invasive along the Mediterranean coast from Egypt to Tunisia, an early introduction through the Suez Canal (Winters *et al*., 2020).

### (5) Animalia

#### (a) Historical trends

The number of alien animal species has increased continuously for all taxonomic groups in all African regions (Fig. 4), similar to the observed global patterns (Fig. 1). European acclimatisation societies were very active, particularly in South Africa, and introduced many plant, bird, and mammal species to ‘improve’ landscape aesthetics to conform with European preferences around the mid‐1800s (Osborne, 2000; van Wilgen *et al*., 2020). Several islands off the African coasts, such as St. Helena, Mauritius, and La Réunion, played important strategic roles for international shipping and ocean crossings during colonial times (Cheke & Hume, 2008), and consequently have many early records of species introductions (Fuller & Boivin, 2009). In São Tomé and Príncipe, species introductions by Europeans began in the 1470s with 14 alien mammal species established on São Tomé and 12 on Príncipe (Dutton, 1994). In the 20th century, increasing global trade accelerated alien species introductions across Africa and, combined with the advent of the game‐farming industry and ecotourism, resulted in a striking rise in introductions of alien vertebrates and invertebrates (Picker & Griffiths, 2017; Measey, Hui & Somers, 2020; van Wilgen *et al*., 2020; Muñoz‐Mas *et al*., 2023).

For mammals, birds, and insects, documented increases tended to be higher in East Africa and adjacent islands, mostly because of many early species introductions on the islands of Mauritius and La Réunion that were not recorded elsewhere. For fishes and crustaceans, particularly high increases of new alien species were recorded in North Africa after 1869 (Fig. 4) when the Suez Canal opened and initiated the Erythraean invasion of marine species (Galil, 2023), a trend that continues until today (Fig. 4). In South Africa, the overall rate of introductions of alien freshwater animals accelerated sharply after 1880 and generally increased over time, with unintentional introductions of invertebrates playing a relevant role (Weyl *et al*., 2020). Only freshwater fish introductions decreased significantly after the 1950s, probably due to new legislation regulating such introductions, which decreased demand for new angling species (Faulkner, Robertson & Wilson, 2020). In general, the number of invertebrate introductions to South Africa rose over time (Faulkner *et al*., 2020), this pattern being reported for freshwater (Weyl *et al*., 2020), terrestrial (Janion‐Scheepers & Griffiths, 2020), and marine invertebrate introductions (Robinson, Peters & Brooker, 2020). The observed rise in alien species seems affected by increased sampling intensity: while only four marine alien species were reported before 1900 (Robinson *et al*., 2020), several other species were likely introduced much earlier (Carlton & Schwindt, 2024).

#### (b) Current status

The African continent currently harbours 44 alien mammals (Biancolini *et al*., 2021), although there is a range of 30–80 reported alien mammals depending on the source (Table 4). These alien species are mainly concentrated along the Western Mediterranean coast, South Africa, and Madagascar. Alien mammals can be found on each group of the 28 island groups in the Western Indian Ocean, with an average richness of five species per island group (Russell *et al*., 2016). There are 12 invasive alien mammal species on La Réunion and six of them on the nearby Îles Éparses (Russell & Le Corre, 2009). Most alien bird species in Africa are found in the far south of the continent, although *Corvus splendens* (house crow) is distributed from Sudan to South Africa along the East coast. The islands of East Africa are important hubs of alien reptiles and amphibians globally: Mauritius and La Réunion are inhabited by 17 and 15 alien species, respectively (Kraus, 2009; Capinha *et al*., 2017; Telford, Channing & Measey, 2019).

Although insects have the highest species richness in general, their proportional representation in Table 4 is still less than expected (344 alien insects out of at least 4510 alien species; Table 4), likely due to under‐reporting of alien insects in the African continent. South Africa is the only African country with a comprehensive list of alien insect species, comprising 300 out of 571 alien animal species in South Africa (Picker & Griffiths, 2017). Most alien arthropods (including insects) in Africa are terrestrial, with the Hemiptera comprising the largest fraction, followed by the Coleoptera (Janion‐Scheepers & Griffiths, 2020). Most insects are introduced accidentally and the Hemiptera are known to be common contaminants of plants that are shipped internationally for propagation in agriculture, forestry or horticulture (Liebhold *et al*., 2012). Some of the most impactful insect invaders, which are widely distributed in Africa, include *Spodoptera frugiperda* (fall armyworm) and *Tuta absoluta* (tomato leafminer), both of which have severe impacts on food production in many African countries (Day *et al*., 2017; Rwomushana *et al*., 2019).

East Africa and its adjacent islands have the second highest numbers of alien fishes, probably because of introductions in the many lakes of the Rift Valley area, including the three largest, Lakes Victoria, Tanganyika, and Malawi (Craig, 1992; Pitcher & Hart, 1995), and Lake Naivasha (Kenya), where the rates of introductions have increased steadily since the 1950s (Gherardi *et al*., 2011). Twenty‐one alien freshwater fishes have been established in South Africa (Ellender & Weyl, 2014; Weyl *et al*., 2020), while 16 alien fish species have been introduced in Central Africa (Brooks, Allen & Darwall, 2011) (26 alien fishes have been reported in total, Table 4). In Madagascar, one quarter of the freshwater fish fauna consists of alien species, with 26 alien species present (Šimková *et al*., 2019). On La Réunion, six species of fish and one decapod crustacean, *Macrobrachium rosenbergii* (giant freshwater prawn) were introduced by 2002, but only four were established by then (Keith, 2002).

Five freshwater alien crayfish have established populations in the wild, of which three have spread widely across Africa: *Procambarus clarkii* (red swamp crayfish), *P. virginalis* (marbled crayfish), and *Cherax quadricarinatus* (redclaw crayfish) (Madzivanzira *et al*., 2021). Seventy‐seven alien freshwater animals, with numbers largely dominated by fishes, molluscs, and crustaceans, are currently established in South Africa, most of which were intentionally introduced (Picker & Griffiths, 2017; Weyl *et al*., 2020). Many species of alien molluscs have been recorded in African fresh waters, with 14 species of gastropods reported by 2011, some of which were released for the biological control of the intermediate hosts of schistosomiasis (Appleton & Brackenbury, 1998; Appleton, 2003). Only a few alien freshwater bivalves have been recorded in African waters, such as *Corbicula fluminea* (Asian clam) and *Sinanodonta woodiana* (Chinese pond mussel), both probably related to fish stocking (Darwall *et al*., 2011; Clavero *et al*., 2012; Mabrouki & Taybi, 2022). Among alien freshwater jellyfish, the cnidarian *Craspedacusta sowerbii* (peach blossom jellyfish) has been recorded in South Africa and Morocco (Oualid *et al*., 2019; Weyl *et al*., 2020).

Information about marine alien species is mostly limited to certain coasts, mainly in South Africa and North Africa: high numbers of marine alien species had been reported by 2020 along Mediterranean and Red Sea coasts of Northern African countries, such as Morocco (24), Algeria (41), Tunisia (166), Libya (77), and Egypt (266) (Galanidi *et al*., 2023). Numbers have since increased to 39 species in Moroccan Mediterranean waters (Mghili *et al*., 2024). Erythraean species are clearly expanding their distribution westwards along the African coast, with over 60% of alien species recorded in Tunisian waters considered to have been introduced through the Suez Canal (Ounifi‐Ben Amor *et al*., 2015). A strong positive relationship between the time elapsed since Erythraean species' first record in the Mediterranean and its westward spread calls attention to a considerable invasion debt advancing westwards (Galil *et al*., 2021). Along the South African coast, which includes two large marine ecosystems, the Agulhas current in the East and the Benguela current in the West (Mead *et al*., 2011; Robinson *et al*., 2020), 95 alien marine species have been reported, of which 56 are considered invasive. A variety of taxa are represented, from small protists (e.g. *Mirofolliculina limnoriae*) and dinoflagellates (e.g. *Alexandrium minutum*) to the most conspicuous macroalgae, molluscs, crustaceans, bryozoans, and tunicates. The most common taxa are ascidians (e.g. *Ciona robusta* and *Botryllus schlosseri*; Peters, Sink & Robinson, 2017). Populations of *Diadumene leucolena* (sea anemone) have been recorded from Senegal (Glon *et al*., 2020).

## VI. THE AMERICAS

### (1) Bacteria and Protozoa

Among Bacteria, 14 alien Proteobacteria have been reported in the SInAS database for The Americas. A well‐known case is the introduction of *Vibrio cholerae*, causing cholera in humans, which has been introduced repeatedly and to different regions of The Americas (Colwell, 1996; Louis *et al*., 2003). The introduction of *V. cholerae* through ballast water was considered the cause of a severe cholera outbreak in South America (Colwell, 1996) but it has also been detected in the Chesapeake Bay, USA (Louis *et al*., 2003). The introduction of *Yersinia pestis*, the agent of bubonic plague, which has been reported from multiple countries in South America, is another example. Agents of infectious diseases, such as the variola virus (smallpox virus; now eradicated globally), *Measles morbillivirus* (measles virus), Bacteria causing typhus, and *Vibrio cholerae* (cholera), were introduced through European colonisation mostly accidentally, although in some cases diseases were intentionally introduced to decimate populations of Native Americans (Oldstone, 2020). Other alien prokaryotes represent plant pathogens, including different species of the genus *Xanthomonas* (*X. campestris* and *X*. *axonopodis*), which have been reported from various countries throughout The Americas. Among protozoans, three species have been recorded as alien in Canada, USA, and Mexico, namely *Haplosporidium nelsoni*, *H. costale*, and *Glugea hertwigi* (Mills *et al*., 1993; Pederson *et al*., 2017; Simpson *et al*., 2018). These species are pathogens of oysters and fish.

### (2) SAR

For The Americas, a total of 150 species of SAR are included in the SInAS database (Table 5). One of the earliest records is the oomycete *Phytophthora cinnamomi* from 1850 in the USA (Aukema *et al*., 2010). This species is considered as one of the most devastating plant pathogens globally, with a widespread distribution and a broad host range of around 5000 known plant hosts (Hardham & Blackman, 2018). Several other species of the same genus have been reported. For example, the sudden oak death pathogen, *P. ramorum*, has infected ecologically, economically, and culturally important genera in North America including *Quercus* and *Notholithocarpus* spp. (McPherson *et al*., 2005). *Phytophthora lateralis* caused declines in the Port‐Orford‐Cedar, endemic to the Eastern USA, with first reports in natural stands from the 1950s (Jung *et al*., 2018).

**Table 5:** Numbers of established alien species for subregions of The Americas. The numbers are extracted from the SInAS database (see Section II) and may deviate from those reported in regional studies. The numbers should be considered as minimum values as the true level of invasion is likely much higher. For mammals, birds, and vascular plants, ranges of values indicate variation among databases. Duplicated taxa for the same region and Totals were removed. SAR, Stramenopila, Alveolata, Rhizaria.

	Caribbean	Mesoamerica	North America	South America	Totals
Mammals	35–62	8–34	49–95	25–77	83–164
Birds	110–113	29–41	210–211	53–114	249–287
Fishes	91	226	619	144	803
Reptiles	60	60	121	56	192
Amphibians	20	8	41	16	62
Insects	153	163	2116	640	2636
Arachnids	33	36	168	76	207
Molluscs	26	60	212	68	255
Crustaceans	10	64	173	79	248
Vascular plants	1402–1761	1600–2242	6571–7424	2492–3099	8005–9325
Algae	2	14	32	21	55
Bryophytes			40	32	61
Fungi	17	15	174	219	363
SAR	4	93	40	34	150
Bacteria and protozoans	1	4	6	5	14
Totals	1964–2353	2380–3060	10,572–11,472	3960–4680	13,383–14,822

Other widespread SAR are marine species, such as the brown algae *Sargassum muticum* (wireweed), *S. fluitans*, and *Undaria pinnatifida*, or the dinoflagellate *Gymnodinium catenatum* (Williams, 2007; Simpson *et al*., 2018). A high number of 94 alien SAR was reported in Mexico according to the GRIIS database (González Martínez *et al*., 2020), followed by the USA (Simpson *et al*., 2018), and Argentina (Zalba *et al*., 2021). For a few countries comprehensive assessments of alien species have been conducted, which also list SAR. For example, five marine SAR have been reported for Chile (Fuentes *et al*., 2020) and 12 for the USA (Simpson *et al*., 2018), most of them being members of Ochrophyta (brown algae).

### (3) Fungi

While it is difficult to trace its initial introduction date, one early introduction was black stem rust of wheat (*Puccinia graminis*) with an epidemic in the USA in 1878. A colony in Rio Grande do Sul, Brazil, reported yield losses due to stem rust already in the 1700s (CABI, 2021). Earlier introductions of other fungal species likely occurred unnoticed. First reports of a cacao disease matching the infestation by *Moniliophthora roreri* (frosty pod rot of cacao) appeared in 1817 in Colombia, which is outside its presumed native range (Bailey *et al*., 2018). Two important forest pathogens are *Cronartium ribicola* (white pine blister rust), first reported in the USA in 1906 (Geils, Hummer & Hunt, 2010), and *Cryphonectria parasitica* (chestnut blight), first reported in the USA in 1904 (Rigling & Prospero, 2018), both having caused significant impacts to native biota. *Cryphonectria parasitica* has rendered *Castanea dentata* (American chestnut) functionally extinct in North America (Dutech *et al*., 2012). *Ophiostoma ulmi* (Dutch elm disease), introduced to North America in 1928, and followed by the more aggressive *O. novo‐ulmi*, severely impacted *Ulmus americana* (American elm) in the USA and Canada (Copeland *et al*., 2023). In South America, *Hemileia vastatrix* (coffee leaf rust) was successfully intercepted in 1903 in Puerto Rico, but established in Brazil in the 1970s, marking the onset of the third great pandemic of coffee rust globally (McCook, 2006). First records of alien fungi in Chile were documented beginning in the early 20th century and have shown a continuous increase until the present (Fuentes *et al*., 2020).

Altogether, 363 alien fungi are reported in the SInAS database for The Americas (Table 5). The USA has the highest number of alien fungi (136), followed by Chile (81), Brazil (80), and Argentina (65) (see also Simpson *et al*., 2018; Fuentes *et al*., 2020). Two widespread and well‐known alien fungal pathogens in The Americas are *Batrachochytrium dendrobatidis* (chytrid fungus), which has caused massive population declines and extinctions in amphibian species since the 1960s (Fisher, Garner & Walker, 2009), and *Claviceps africana* (ergot).

The Americas harbour at least 199 alien macrofungi species, with approximately 36% belonging to the order Agaricales, 32% to Boletales and 11% to Russulales (Monteiro *et al*., 2020). Some of the most widely distributed are ectomycorrhizal fungi, such as *Suillus luteus*, *Rhizopogon roseolus*, and *Suillus granulatus* (weeping bolete), which seemed to play a key role in pine invasions of the Southern Hemisphere (Policelli *et al*., 2019). South American countries with high numbers of known alien fungi include Brazil (75), Argentina (60), and Chile (40) (Monteiro *et al*., 2020). In the remaining IPBES subregions, higher numbers of alien macrofungi were recorded in the USA (50), Canada, and Mexico (seven each).

### (4) Plantae

#### (a) Historical trends

**Fig. 5:**
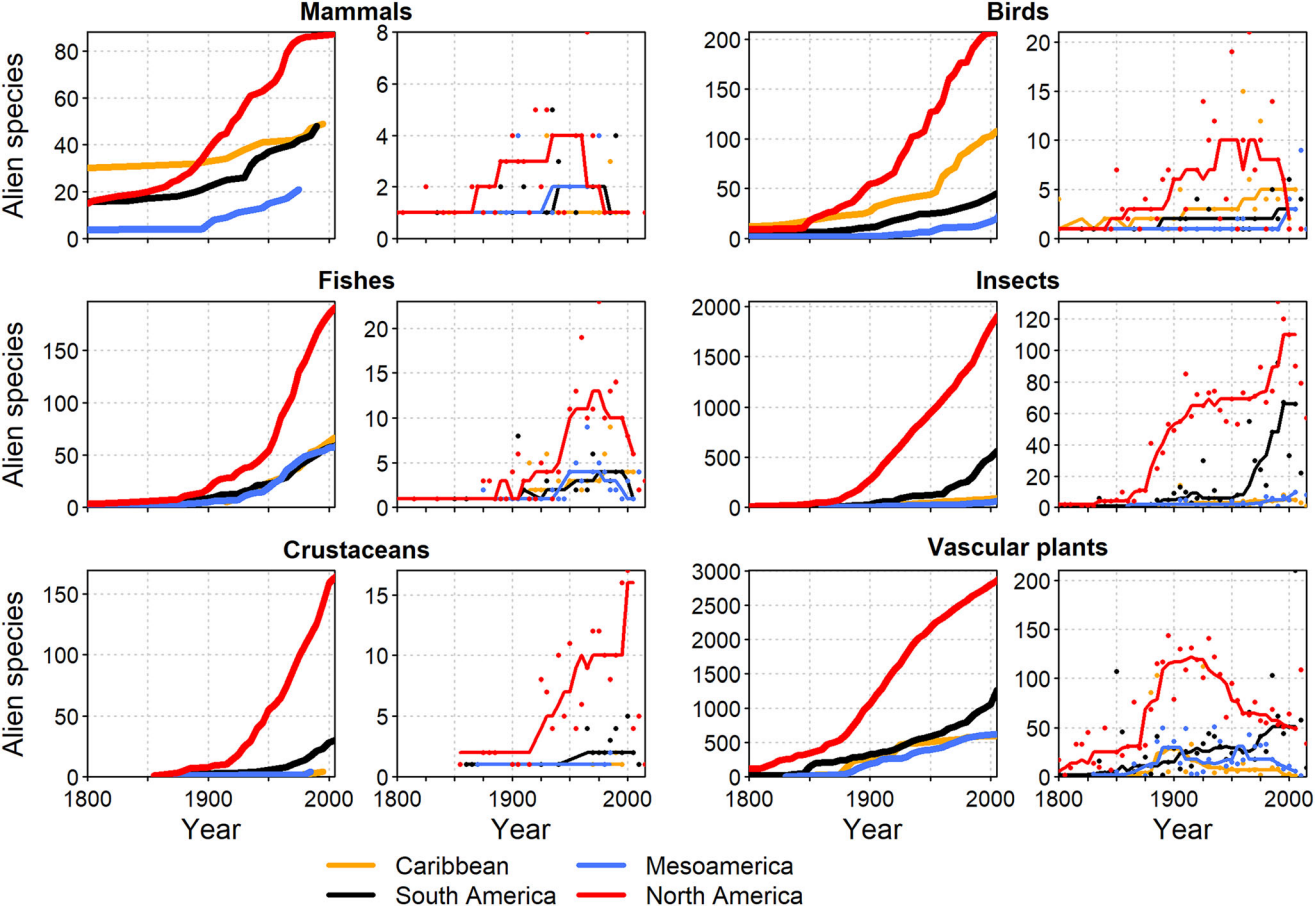
Trends in numbers of established alien species for The Americas. Panels by taxon show cumulative numbers (left panels, thick lines) and number of new alien species per five‐year intervals (right panels, thin lines). Numbers shown here underestimate the real extent of alien species occurrences due to a lack of data. Lines in right panels indicate smoothed trends calculated as running medians. Note that presented numbers may deviate from those reported in the text due to variation among data sources.

The first explorers of the eastern coast of The Americas introduced *Lolium perenne* (perennial ryegrass), and *Trifolium repens* (clover) among others; these alien species were fully established prior to colonisation by Europeans (Crosby, 1986). English colonisation of the North American east coast in the 17th century led to deforestation and the establishment of farms where many European species were cultivated for ornamental [e.g. *Taraxacum officinale* (dandelion)] or medicinal [*Verbascum thapsus* (common mullein)] purposes, directly sown in pastures [*Poa pratensis* (smooth meadow‐grass)], or brought in as weeds [*Capsella bursa‐pastoris* (shepherd's purse)] (Crosby, 1986), or in ship's solid ballast [*Phragmites australis* (common reed)] (Saltonstall, 2002). Over the last two centuries, North America has had the most rapid cumulative rate of increase of alien plant species, accelerating at the end of the 19th century (Fig. 5) (Lavoie *et al*., 2012; Pyšek *et al*., 2019), with macrophytes such as *P. australis* invading across the continent in less than 200 years (Meyerson *et al*., 2025). South America exhibited a slower cumulative increase, likely due to lower research intensity relative to North America (Frehse *et al*., 2016; Schwindt & Bortolus, 2017; Schwindt *et al*., 2020). Nevertheless, as early as 1877, an impressive number of plant species were recorded as introduced in Argentina from Europe through the port of Buenos Aires, from where they rapidly dispersed up to the southernmost end of Patagonia (Berg, 1877; Bortolus & Schwindt, 2022). The rates of newly recorded alien plants began decreasing in the mid‐20th century, particularly in North America (Fig. 5). The magnitude of introductions in the Caribbean and Mesoamerica was much lower, but showed increases similar to those observed in North and South America (Fuentes *et al*., 2008; Ugarte, Fuentes & Klotz, 2010; Rojas‐Sandoval & Acevedo‐Rodríguez, 2015).

#### (b) Current status

North America has the highest number of recorded alien plant species in the world with at least 5958 established taxa (van Kleunen *et al*., 2015; Pyšek *et al*., 2017), while South America harbours 2667 alien plant species (Pyšek *et al*., 2019). Globally, California is richest in alien vascular plants, with 1753 taxa recorded and Florida is another invasion hotspot harbouring 1473 alien plants (Kartesz, 2015). According to Pyšek *et al*. (2017), countries in Mesoamerica also harbour many alien plants (Nicaragua: 671, Mexico: 519, Costa Rica: 280, Panama: 263), but due to their high native diversity, alien plants make up only 2.0–2.8% of the total floras, the exception being Nicaragua with 10.4% (Correa A., Galdames & De Stapf, 2004; Chacón & Saborío, 2012; Pyšek *et al*., 2017). Some regions in the Caribbean are heavily invaded by alien plants, both in terms of actual species numbers (Cuba: 542, Bahamas: 356) and the proportion of alien plants in the national floras (Bahamas 24%, Barbados 14%). Other countries in the Caribbean harbour 20–110 alien plants and contributions to national floras do not exceed 8% (Acevedo‐Rodríguez & Strong, 2008; Kartesz, 2015; Pyšek *et al*., 2017).

Alien species are widespread on islands along both the Pacific and Atlantic coasts of The Americas, notably the Caribbean islands (Kairo *et al*., 2003; Van der Burg *et al*., 2012; Rojas‐Sandoval & Acevedo‐Rodríguez, 2015). As an example, parts of Caribbean island forests are dominated by alien tree species (Chinea & Helmer, 2003; Brandeis *et al*., 2009; Helmer *et al*., 2012), some of which are shade tolerant and could permanently change forest species composition (Brown *et al*., 2006). In addition, several alien species invade forest plantations, livestock pastures, and abandoned agricultural fields causing both economic and environmental impacts. Such is the case for *Dichrostachys cinerea* (sickle bush), an alien species of African origin that occurs across almost 800,000 hectares in Cuba (Hernández, Lahmann & Pérez‐Gil Salcido, 2002).

An example of a widespread marine plant is *Halophila stipulacea* (broadleaf seagrass; native to the Indian Ocean and Red Sea), which invaded the coasts of the Mediterranean Sea as early as 1895 (Winters *et al*., 2020), and then the Caribbean Sea by 2002 (Willette *et al*., 2014). Compared to the slow spread of this seagrass across the Mediterranean from Egypt to Tunisia (~120 years), it invaded coasts from Puerto Rico to Venezuela in just 15 years (Winters *et al*., 2020), becoming the world's first circumglobal marine alien angiosperm. The seagrass *Zostera japonica* (dwarf eelgrass) was introduced to the Pacific northwest in the mid‐1900s likely *via* oyster aquaculture and has since spread and negatively impacted native *Zostera marina* (eelgrass) and ecosystem processes (Shafer, Kaldy & Gaeckle, 2014).

The Galapagos Archipelago harbours an estimated 1700 alien species with *Rubus niveus* (Mysore raspberry) being among the most common (Toral‐Granda *et al*., 2017), posing a serious threat to the foundation species of native forest, endemic daisy tree *Scalesia pedunculata* (Riegl *et al*., 2023) and, together with *Cestrum auriculatum* and *Tradescantia fluminensis*, to the entire forest (Jäger *et al*., 2024). Between the 1980s and 1990s, the number of alien plants has nearly doubled on the Galapagos Islands, reaching nearly 900 species (Torres & Mena, 2018). A study of the residence time and human‐mediated propagule pressure of plants suggests that this archipelago is still in an early stage of plant invasions, due to the booming tourism industry and increasing human population size (Trueman *et al*., 2010).

### (5) Animalia

#### (a) Historical trends

The number of alien animals in The Americas has continuously increased across all taxonomic groups, especially post‐1850, and across all subregions (Fig. 5). Particularly sharp increases were observed for North America, followed by South America, while alien birds steeply increased in the Caribbean. The first introductions of alien mammals date to pre‐Columbian times in the Caribbean islands, e.g. *Didelphis marsupialis* (common opossum) and *Dasyprocta leporina* (agouti) (Long, 2003; Giovas, LeFebvre & Fitzpatrick, 2012; Biancolini *et al*., 2021). The number of alien species introductions began surging in the 15th century with European colonisation, peaked in the 20th century with many introduced game species, and more recently, *via* the pet trade (Long, 2003; Biancolini *et al*., 2021). Since 1900, rates of increase have declined in a few cases, particularly for mammals and fishes (Fig. 5). The number of alien amphibians and reptiles in The Americas has increased since the 1950s with new alien species introductions *via* the pet trade projected to remain steady or accelerate (Kraus, 2009; Powell *et al*., 2011; Stringham & Lockwood, 2018; Lockwood *et al*., 2019; Perella & Behm, 2020).

Several studies indicate increasing numbers of alien insects in North America (Mattson *et al*., 1994; Aukema *et al*., 2010; Nealis *et al*., 2016). Some of the earliest alien insect introductions were ground‐dwelling beetles, such as Carabidae, accidentally transported from Europe with rocks used as ship ballast (Lindroth, 1954). During the early 1900s, many insect species, such as *Quadraspidiotus perniciosus* (San Jose scale), were accidentally introduced with plants imported for agriculture and horticulture (Liebhold & Griffin, 2016). In South America, the number of reported alien aquatic organisms is rapidly increasing (e.g. Fuentes *et al*., 2020; Schwindt *et al*., 2020; Vitule *et al*., 2021). The first alien aquatic species introductions occurred in the 1500s in conjunction with European colonisation. The number of introductions remained low in the 1800s and 1900s, but increased thereafter (Vitule *et al*., 2021). Beginning in the 2000s, both the number of records and the number of studies on alien organisms increased continuously (e.g. Frehse *et al*., 2016; Vitule *et al*., 2021), with no sign of slowing down, both in terms of alien species numbers and new spatiotemporal records (e.g. Vitule *et al*., 2021).

For marine alien species, seminal studies have highlighted rising numbers in American waters (Cohen & Carlton, 1998; Carlton & Eldrege, 2015; Carlton & Schwindt, 2024). For example, in temperate coastal waters of The Americas, new data show an increase in the total number of alien species detected, while the rate of new detections of alien species has stabilised (Bailey *et al*., 2020). Following a distinct intensification of research efforts on marine alien species in South America between 1997 and 2014 (Schwindt & Bortolus, 2017), the number of introduced species increased by 160% between 2009 and 2019 in Brazil (Teixeira & Creed, 2020). In Argentina and Uruguay, marine alien species numbers increased by a factor of 4.5 between 2001 and 2019, with one new species estimated to arrive every 178 days (Schwindt *et al*., 2020).

#### (b) Current status

The Americas host 96 species of alien mammals with particularly high species numbers on the East coast of North America, Alaskan islands, Southern USA, the Caribbean Archipelago, Patagonia, and Falkland Islands (Malvinas) (Biancolini *et al*., 2021). The number of reported alien mammals ranges from 83 to 164, depending on the source (Table 5). One of the most widespread alien mammals in The Americas is *Urva auropunctata* (small Indian mongoose) which has established on many islands in the Caribbean (Hays & Conant, 2007; Louppe *et al*., 2020; Biancolini *et al*., 2021). North America is particularly rich in alien bird species, notably in Florida and California where several alien parrot species have established populations (Dyer, Redding & Blackburn, 2017b). The Americas have the highest number of alien reptiles and amphibians in the world with several hotspots in the USA (Kraus, 2009; Krysko *et al*., 2011, 2016; Capinha *et al*., 2017), including Florida, California, and Puerto Rico (Meshaka, 2011; Powell *et al*., 2011; Perella & Behm, 2020). Other Caribbean islands, such as Cuba and the Bahamas, are also important global alien amphibian hotspots (Kraus, 2009; Knapp *et al*., 2011; Powell *et al*., 2011; Borroto‐Páez *et al*., 2015). In South America, Brazil has the highest number of alien amphibian and reptile species (136), of which at least seven have established wild populations (Kraus, 2009; Fonseca, Both & Cechin, 2019). Globally, North America has the most alien insect species (Liebhold *et al*., 2016) with the largest concentration of species in Northeastern North America (Liebhold *et al*., 2013). Even though there are more described species of Coleoptera than any other order, there are slightly more alien Hemiptera species established in North America (Yamanaka *et al*., 2015; Liebhold *et al*., 2016) and Chile (López *et al*., 2023). The over‐representation of the Hemiptera is most likely the result of the ease with which they are accidentally introduced with imported plants.

The most recent studies of freshwater fishes in South America indicate that more than 75 alien species have been translocated between different basins within South America (Bezerra *et al*., 2019; Vitule *et al*., 2019) and more than 80 alien fish species from other regions of the world (Vitule *et al*., 2019, 2021; Doria *et al*., 2021). A famous example of an alien aquatic invertebrate is *Limnoperna fortunei* (golden mussel) (Schwindt & Bortolus, 2017). North America has a long history of aquatic species introductions, particularly for fish, such as *Salmo trutta* (brown trout) or *Cyprinus carpio* (common carp) (Moyle, 1986; Courtenay & Meffe, 1989; Fuller, Nico & Williams, 1999), as well as many crustaceans and molluscs, such as *Bythotrephes longimanus* (spiny waterflea), *Dreissena* spp. (zebra and quagga mussels), and *Corbicula* spp. (basket clams). The Laurentian Great Lakes have been invaded by nearly 190 alien species (Ricciardi, 2006; Ricciardi & MacIsaac, 2022), more than any other freshwater ecosystem in the world. These species include several invasive alien animals of Ponto‐Caspian origin, mostly introduced through ballast water (Ricciardi & MacIsaac, 2000; Ricciardi, 2001, 2006; Vanderploeg *et al*., 2002). Many species native to small regions of The Americas, such as *Faxonius rusticus* (rusty crayfish), *Gambusia* spp. (mosquitofishes), and salmonids, such as *Oncorhynchus mykiss* (rainbow trout), have been widely introduced throughout The Americas and elsewhere (Marr *et al*., 2013; Schwindt *et al*., 2018; Muñoz‐Mas *et al*., 2023). A spectacular case is the North American beaver (*Castor canadensis*), which was introduced to the Tierra del Fuego Archipelago in 1946 and negatively impacts *Nothofagus* forests; subsequently, abandoned beaver ponds become hotspots for the spread of alien pasture plants (Skewes *et al*., 2006).

Studies of marine alien species across The Americas are geographically and taxonomically patchy. Spatial and temporal surveys are scarce, even in well‐studied regions, such as the USA, making it difficult to draw general conclusions (Bailey *et al*., 2020). The first comprehensive assessment was for the continental coasts of the USA, finding 298 marine alien species (Ruiz *et al*., 2000), but by 2006, 257 marine alien species were identified in California alone (Ruiz *et al*., 2011). In South America, the Southwestern Atlantic region is the most‐investigated area for marine invasive alien species, with fish and molluscs represented in the largest numbers of studies, species, and spatiotemporal occurrence records (Frehse *et al*., 2016; Schwindt & Bortolus, 2017; Bezerra *et al*., 2019). Brazil has the highest number with 138 marine alien species (Teixeira & Creed, 2020), followed by Argentina and Uruguay with 129 alien species in total (Schwindt *et al*., 2020). On the Pacific coast, Chile reported 51 marine alien species (Castilla & Neill, 2009; Villaseñor‐Parada, Pauchard & Macaya, 2017) and Colombia four (Gracia *et al*., 2011). As with essentially all coasts worldwide, the numbers of introductions are distinctly under‐estimated due to an absence of historical baselines and a lack of research (Schwindt & Bortolus, 2017; Carlton & Schwindt, 2024). For example, the number of marine alien species in Chile is likely to be much higher than the reported 51 (Stowhas Salinas *et al*., 2023).

## VII. ASIA & THE PACIFIC

### (1) Bacteria and Protozoa

Little is known about alien prokaryotes with very few reports in Asia & the Pacific. Chen, Sun & Zhan (2017b) reported three Proteobacteria in China, *Pseudomonas syringae*, *Acidovorax avenae*, and *Xanthomonas oryzae* (leaf blight), which are known for their role as plant pathogens, and one marine cyanobacterium *Trichodesmium erythraeum*. The proteobacterium and plant pathogen *Xanthomonas axonopodis* has been recorded across multiple Pacific Islands from Australia to the Hawaiian Islands (EPPO, 2016; Nahrung & Carnegie, 2020). *Erwinia amylovora* (fire blight) has been recorded for multiple countries mostly on mainland Asia (Vanneste, 2008).

### (2) SAR

The SInAS database includes 103 taxa of SAR for Asia & the Pacific (Table 6). While the earliest record is *Phytophthora infestans* (potato blight) from 1900 (APASD, 2024), the number of records showed a strong increase starting in the 1970s and continuing until today. Soilborne plant pathogens like *P. cinnamomi*, which originates from Southeast Asia (Hardham & Blackman, 2018; Thakur *et al*., 2019), and *P*. *ramorum* (sudden oak death) are also widely distributed and occur on several hosts in most countries in Asia (Davison, 2022; Garbelotto & Frankel, 2020) including several Australian islands (Auld & Hutton, 2004; Pickering, Bear & Hill, 2007), Hawaii (Davison, 2022), Fiji, Samoa, Tuvalu, and New Zealand (Campbell, 2010; Thaman, 2011; Thaman & O'Brien, 2011). Other frequently recorded species are the brown alga *Undaria pinnatifida* and the dinoflagellate *Alexandrium minutum*. A large number of the taxa reported in the SInAS database are marine (96 species), including species of the genus *Alexandrium*, *Sargassum*, and *Rhizosolenia*. For China alone, 60 species of SAR (in all realms) have been reported with the majority being Ochrophyta (31 species), such as five species of the genus *Rhizosolenia*, followed by Myzozoa (21) (Xu *et al*., 2012; Chen *et al*., 2017b). For Australia, 13 species have been recorded in the SInAS database, of which six were identified as forest pests (Nahrung & Carnegie, 2020).

**Table 6:** Numbers of established alien species for subregions of Asia & the Pacific. The numbers are extracted from the SInAS database (see Section II) and may deviate from those reported in regional studies. The numbers should be considered as minimum values as the true level of invasion is likely much higher. For mammals, birds, and vascular plants ranges of values indicate variation among databases. Duplicated taxa for the same region and Totals were removed. SAR, Stramenopila, Alveolata, Rhizaria.

	Northeast Asia	Oceania	South Asia	Southeast Asia	Western Asia	Totals
Mammals	28–53	50–105	12–28	38–54	5–20	97–163
Birds	119–129	169–175	29–38	84–85	84–139	287–336
Fishes	287	95	90	296	125	633
Reptiles	41	41	7	35	13	103
Amphibians	24	13	4	12	1	43
Insects	607	1521	111	89	101	2017
Arachnids	67	83	13	18	6	129
Molluscs	81	119	15	24	89	261
Crustaceans	43	75	12	19	63	158
Vascular plants	2219–2454	4631–6747	1055–3142	1313–1598	271–562	6141–9101
Algae	5	37	4	7	27	66
Bryophytes	3	43				44
Fungi	59	303	17	20	1	363
SAR	59	31	6	7	20	103
Bacteria and protozoans	7	4	3	2	4	12
Totals	3649–3919	7215–9392	1378–3490	1964–2266	810–1171	10,457–13,532

### (3) Fungi

For Asia & the Pacific, the highest numbers of alien fungi are reported in New Zealand (180 species) and Australia (132) according to the SInAS database. However, the database *Biota of New Zealand* lists more than 2000 alien fungi just for New Zealand, indicating the huge discrepancy among databases (Manaaki Whenua – Landcare Research, 2024). One of the earliest reports was *Hemileia vastatrix* (coffee leaf rust), which caused an epidemic in Southern India and Sri Lanka (Ceylon) in 1869 and spread further across coffee‐producing regions in the Indian Ocean and Pacific (McCook, 2006). Data from China indicate that of the 27 known alien fungi, only two new additions were reported after 2000 (Xu & Qiang, 2018). Fifteen alien fungal pathogens were intercepted by plant quarantine in India (Akhtar *et al*., 2019, 2021; Dubey *et al*., 2021) between 2015 and 2020. Another significant plant pathogen is *Ceratocystis lukuohia*, the causal agent of Rapid ‘Ōhi‘a Death (ROD), reported since 2010 and causing high mortality in Hawaii's endemic keystone tree species *Metrosideros polymorpha* (Fortini *et al*., 2019).

As for other regions in the Southern Hemisphere, the extensive cultivation of eucalypts and pines led to the co‐invasion of a suite of associated fungi (Vellinga, Wolfe & Pringle, 2009). Ectomycorrhizal fungal communities of *Pinus contorta* in New Zealand were dominated by alien fungi, highlighting co‐invasion as an important process (Dickie *et al*., 2010). In tropical Asia, pathogens of alien eucalypts, such as species of the genera *Mycosphaerella*, *Teratosphaeria*, and *Aulographina eucalypti* and *Cryphonectria cubensis*, were apparently co‐introduced from Australia (Sankaran & Hussain, 2019). Ectomycorrhizal fungi of eucalypts, such as *Laccaria fraterna*, *Pisolithus albus*, and *P. arrhizus*, are alien species introduced into the region. The myrtle rust, *Austropuccinia psidii* (originally from South America), has been recorded in China, Indonesia, Japan, New Zealand, Australia, Singapore (Carnegie & Giblin, 2022), and New Caledonia (Soewarto *et al*., 2018). Other alien fungi recorded from the region include *Ceratocystis fimbriata sensu lato* (wilt of several hosts), *Melampsora medusae* (leaf rust of poplars), and *Puccinia horiana* (white rust) (Akhtar *et al*., 2019; CABI, 2022; EPPO, 2024). Twenty‐seven invasive alien fungal pathogens were recorded from China (Xu & Qiang, 2018), 21 from India (Government of India, 2005; Akhtar *et al*., 2019, 2021; Dubey *et al*., 2021), 30 from the Maldives (Shafia & Saleem, 2003), and 15 from the Lao People's Democratic Republic (Nhoybouakong & Khamphouke, 2003). Of the 42 powdery mildew species (*Erysiphales*) found in Australia, all are classified as introduced, mainly since the European colonisation of the continent (Kiss *et al*., 2020). However, it is clear from studies by Fisher *et al*. (2020) that several new alien fungi may have been introduced to the region from across the globe and the numbers are grossly underestimated. In addition, limited historical data on fungi hinder the identification of native and alien status with any degree of certainty.

A comparatively high number of alien macrofungi have been reported for Asia & the Pacific, which harbours at least 235 alien species (Monteiro *et al*., 2020). The majority belong to the order Agaricales (54%), followed by Boletales (21%), and Russulales (10%). The most widespread alien macrofungus is *Pyrrhoderma noxium*. Available data indicate that the highest numbers of alien macrofungi are known in New Zealand (170 species) followed by Australia (40 species).

### (4) Plantae

#### (a) Historical trends

**Fig. 6:**
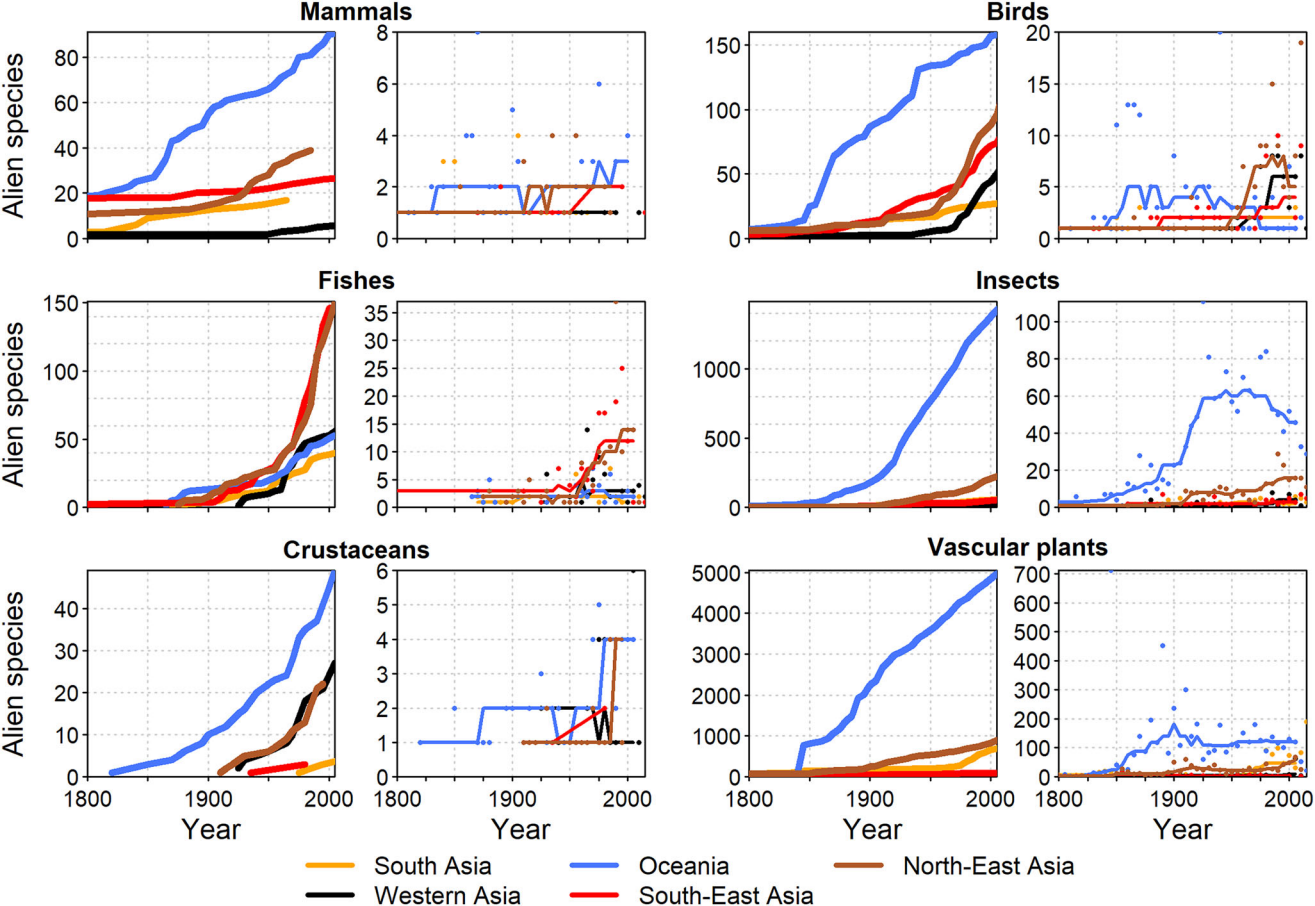
Trends in numbers of established alien species for Asia & the Pacific. Panels by taxon show cumulative numbers (left panels, thick lines) and number of new alien species per five‐year intervals (right panels, thin lines). Numbers shown here underestimate the actual extent of alien species occurrences due to a lack of data. Lines in right panels indicate smoothed trends calculated as running medians. Note that numbers presented may deviate from those reported in the text due to variation among data sources.

First records of alien plant species in Asia & the Pacific date back more than 1000 years (Wijesundara, 2010) and consistent increases in the number of alien species have been recorded for several Asian and Pacific countries (Wijesundara, 2010; Wu *et al*., 2010; Lazkov & Sultanova, 2011; Jaryan *et al*., 2013; Shrestha, 2016; Vinogradov & Kupriyanov, 2016; Chen *et al*., 2017a; Pant *et al*., 2021; Cáceres‐Polgrossi *et al*., 2023). The strongest increase in the cumulative number of alien plant species is recorded for Oceania, including Australia, New Zealand, and the Pacific Islands (Fig. 6). Introduction rates peaked around 1900, followed by a decline, and re‐acceleration in the mid‐20th century (Fig. 6). The trends for other Asian regions are similar to that for Oceania but have markedly lower absolute numbers of alien species per time period.

#### (b) Current status

Regions in Asia & the Pacific include global hotspots of alien plant species (Dawson *et al*., 2017), and this holds true for islands in Oceania (Moser *et al*., 2018; Essl *et al*., 2019). Examples include New Zealand with 1798 alien plants (Brandt *et al*., 2021), Tahiti (1346), and Guam (833; Raulerson, 2006). Australian states harbour from 1186 established species in Western Australia to 1584 in New South Wales, corresponding to 12–25% of the total plant diversity in these states (Randall, 2002; Walsh & Stajsic, 2007; Pyšek *et al*., 2017). Australasia has had the most rapid accumulation of numbers of alien plants with expanding colonisation, and the Pacific islands show the steepest increase of all global regions using this measure (van Kleunen *et al*., 2015). The most widespread alien species on the Pacific Islands include *Euphorbia hirta* (garden spurge), *Cenchrus echinatus* (southern sandbur), and *Phyllanthus amarus* (Jamaica weed). In Australia and New Zealand, it is *Sonchus oleraceus* (common sowthistle), *Solanum americanum* (American black nightshade), and *Chenopodiastrum murale* (nettle‐leaf goosefoot) (Pyšek *et al*., 2017).

Global hotspots also occur in other Asian regions; in South Asia, India (471 alien plants; Inderjit *et al*., 2018), the Philippines (628; Pelser, Barcelona & Nickrent, 2011), and Indonesia (503; SEAMEO BIOTROP, 2003) are invasion hotspots. In Northeast Asia, Japan is richest in alien plants (1311) and numbers from China range from 861 to 933 (Hao & Ma, 2023). The most recent inventory based on 236 studies documented the presence of 241 invasive alien plant species across seven countries of South Asia, with the highest invasive species richness in India (185) followed by Bhutan (53), Sri Lanka (45), Bangladesh (39), Nepal (30), Pakistan (29) and the lowest in Maldives (15) (Gulzar *et al*., 2024). Western Asia is comparatively species poor in alien plants (Pyšek *et al*., 2017). The most widespread alien plants in South and Southeast Asia include *Chromolaena odorata* (siam weed), *Lantana camara* (lantana), *Leucaena leucocephala* (leucaena), and *Prosopis juliflora* (mesquite) (Sankaran & Suresh, 2013). The Hawaiian Islands are a global hotspot of plant invasions with 1466 alien plant species, and numbers for individual main islands within the archipelago range from 398 to 963 alien species (Imada, 2019). Alien plants are a serious issue in forests of many Asian Pacific islands, such as Tahiti (Meyer & Florence, 1996), Fiji (Lenz *et al*., 2022; Forey *et al*., 2023), Lord Howe Island (Auld & Hutton, 2004), and Carnac Island (Abbott, Marchant & Cranfield, 2000). The most problematic species in urban areas of the islands include *Mikania micrantha* and *Spathodea campanulata* (Lowry *et al*., 2020).

### (5) Animalia

#### (a) Historical trends

Before European colonisation, alien mammals in Southeast Asia were introduced *via* ancient exchanges between the Indonesian Archipelago, Papua New Guinea, and Australia with numerous prehistoric introductions, such as *Phalanger orientalis* (northern common cuscus), *Sus celebensis* (Sulawesi pig), and *Dendrolagus matschiei* (Matschie's tree‐kangaroo) (Heinsohn, 2003; Long, 2003; Biancolini *et al*., 2021). Since 1500, the numbers of alien animal species increased continuously for all taxonomic groups and all subregions of Asia & the Pacific (Fig. 6). The steepest increases were observed in Oceania, particularly during the 19th century for all considered animal groups, except for fishes, mostly because of European colonisation. In other subregions, steep increases were mostly observed after 1950 for insects (Huang, Haack & Zhang, 2011; Yamanaka *et al*., 2015), gastropods (Barker, 1999; Roll *et al*., 2009), amphibians and reptiles (Lee *et al*., 2019), and marine alien species of different groups (Hewitt *et al*., 2004; Bailey *et al*., 2020). During the 19th century, acclimatisation societies sought to ‘improve’ local fauna by introducing many aesthetically pleasing and game species to Australia and New Zealand (Lever, 1992; Simberloff & Rejmánek, 2011; Pipek, Pyšek & Blackburn, 2015; Pipek, Blackburn & Pyšek, 2019). The Asia & the Pacific region has also experienced a growing number of alien bird, reptile, and amphibian introductions, a trend likely to continue in the future (Kraus, 2009; Keppel *et al*., 2014; Chapple *et al*., 2016; Lee *et al*., 2019; Pili *et al*., 2020; Toomes *et al*., 2020).

The numbers of alien freshwater species grew slowly in Asia & the Pacific until the 19th century (Fig. 6) after which they increased rapidly (Yuma, Hosoya & Nagata, 1998; Tan *et al*., 2020; Muñoz‐Mas *et al*., 2023). During the 20th century, aquaculture was the main pathway for freshwater fish species introductions (Xiong *et al*., 2015; Saba *et al*., 2020). In addition, mosquitofishes (*Gambusia* spp.) and guppy (*Poecilia reticulata*) were widely introduced for the control of mosquitoes and disease while several other freshwater fish species were introduced for ornamental purposes (Yuma *et al*., 1998; Tan *et al*., 2020). The number of ornamental freshwater fish rapidly increased towards the end of the 20th century and the ornamental trade is now the main pathway of introduction (Yuma *et al*., 1998; Goren & Ortal, 1999; Saba *et al*., 2020).

#### (b) Current status

Asia & the Pacific is the region with the highest number of alien mammals in the world (130 species) (Biancolini *et al*., 2021), with numbers ranging from 97 to 163 depending on the data source (Table 6). Areas with high numbers of alien mammals are Japan, the Indonesian archipelago, Australia, New Zealand, and the Pacific islands. For example, *Oryctolagus cuniculus* (European rabbit) is a prominent invasive alien species in Australia (Kirkpatrick, Page & Massam, 2008), and *Trichosurus vulpecula* (brushtail possum) was introduced to New Zealand in 1858 for the domestic fur and meat trade (Gormley *et al*., 2012; Forsyth *et al*., 2018). Mammals have been widely introduced on islands in Asia & the Pacific (Russell *et al*., 2017), with synanthropic species, such as mice, rats, rabbits, pigs, goats, cats, and foxes, causing devastating impacts on insular ecosystems (Russell & Kueffer, 2019). The number of alien bird species in Asia & the Pacific is among the highest within IPBES regions with particularly high numbers recorded for Oceania, followed by Southeast Asia and Northeast Asia (Table 6). More than 100 alien birds are found in tropical Asia, which is likely a consequence of the intensive bird trade in this region (Corlett *et al*., 2020). Particularly high numbers of alien birds were recorded in Australia, New Zealand, Taiwan, and Japan (Dyer *et al*., 2017a). Asia & the Pacific harbours two of the best‐known examples of alien reptiles and amphibians, namely *Boiga irregularis* (brown tree snake) in Guam and *Rhinella marina* (cane toad) in Australia and other Pacific islands (Lever, 2003; Zug, 2013; Rogers *et al*., 2017; Engeman, Shiels & Clark, 2018; Shine, 2018). Invertebrates outnumber vertebrate species, and many examples of alien invertebrate introductions are known. Hawaii is a classic example of an archipelago heavily invaded by many species' groups, being among the three regions with most records of alien species in the world (Dawson *et al*., 2017), including 3000 arthropods (Nishida, 2002). In Guam, but also on Christmas Island, an *Anoplolepis gracilipes* (yellow crazy ant) invasion was assisted by the invasive *Tachardiaephagus tachardiae* (yellow lac scale insect) (O'Dowd, Green & Lake, 2003; Reaser *et al*., 2007). Other typical examples are gastropod invasions on many Polynesian islands, such as *Lissachatina fulica* (giant African land snail) and *Euglandina rosea* (Tsatsia & Jackson, 2022), with disastrous consequences for the endemic gastropod fauna (Gerlach *et al*., 2021).

The number of alien freshwater fishes is highest in China (61) (Luo *et al*., 2019), followed by Singapore (42) (Tan *et al*., 2020), the Philippines (39) (Casal *et al*., 2007), and Japan (23) (Yuma *et al*., 1998). Most of the alien fishes were introduced for aquaculture, while the proportion of introduced ornamental fishes is much lower (Casal *et al*., 2007; Luo *et al*., 2019; Muñoz‐Mas *et al*., 2023). The national status of alien freshwater invertebrates was only reported in China and Singapore, including 13 alien invertebrates in China (Chen *et al*., 2017b) and 14 alien molluscs in Singapore (Ng *et al*., 2016); these figures include only the most conspicuous invertebrates and distinctly underestimate invertebrate introductions. As with fishes, most known invertebrates were introduced *via* aquaculture and ornamental pathways (Ng *et al*., 2016; Chen *et al*., 2017b). On Pacific islands, about 59 freshwater snails, 38 of them cryptogenic, have been introduced (Cowie, 2001). In Pearl Harbour (Hawaii) alone, 191 inland water species of eight phyla but mostly insects, crustaceans, molluscs, and annelids were identified by 1997–1998 (Englund, 2002).

A regional assessment of marine alien species across Asia & the Pacific is lacking, and, as in many other marine regions, the number of actual introductions is sorely underestimated (Carlton & Schwindt, 2024). Large‐scale studies report 73 alien species for the Central Indo‐Pacific, a region subject to intensive maritime traffic importing marine alien species from around the world for more than 500 years (Carlton & Schwindt, 2024). For the Northwest Pacific, 208 species are reported and 368 for the Northeast Pacific (Lee & Reusser, 2012; Kestrup, Smith & Therriault, 2015). Several marine studies exist for individual countries, such as Japan (42 marine alien species) (Iwasaki, 2006), Republic of Korea (41) (Lutaenko *et al*., 2013), and Russia (66) (Zvyagintsev *et al*., 2011). For the South Pacific Ocean, the highest research effort has been around Australia and New Zealand. In Port Phillip Bay (Australia), 100 marine alien species were reported (Hewitt *et al*., 2004), while 214 alien species were reported for New Zealand (Therriault *et al*., 2018). The knowledge of marine alien species of the Pacific Island countries and territories is scattered and dispersed in diverse publications. Surveys in Pago Pago Harbour (American Samoa) recognised 17 marine alien species (Coles *et al*., 2003), 40 alien species were detected from Guam (Paulay *et al*., 2002), and 11 alien species in Malakal harbour, Palau (Campbell, Hewitt & Miles, 2016). We emphasise that few, if any, of these numbers approach reality.

## VIII. EUROPE & CENTRAL ASIA

### (1) Bacteria and Protozoa

With 55 species of alien Bacteria (Magliozzi *et al*., 2022), Europe is the IPBES region with the highest number of species in this group. The main reason for the comparatively high number of species is the availability of a report of alien Bacteria and viruses (Magliozzi *et al*., 2022), which is unique among IPBES regions. According to this report, the vast majority of alien Bacteria in Europe have been introduced from North America (64%), followed by South America (16%). Highest numbers of alien Bacteria were found in Italy, France, and the Netherlands. However, for a large number of species (56%) the invasion status, and thus the native range, could not be identified. Among prokaryotes, the majority of species are Proteobacteria, such as *Erwinia amylovora* (fire blight), and several species of the genera *Xanthomonas* and *Pseudomonas*. The second largest group is Cyanobacteria including the freshwater species *Cylindrospermopsis raciborskii*, *Sphaerospermopsis aphanizomenoides*, and *Raphidiopsis mediterranea*. For Central Asia, *E. amylovora* has been reported for Armenia (Vanneste, 2008). Eight species of alien protozoans have been reported for Europe & Central Asia with *Bonamia ostreae* being the most widespread species. This species is a pathogen of shellfish and was first reported in 1987 in Ireland (Pederson *et al*., 2017). Other species are the marine algae *Poropila dubia*, *Haplosporidium armoricanum*, and *Haplosporidium nelsoni*, the latter two being oyster pathogens.

### (2) SAR

The number of alien SAR has been rising continuously until recently with a peak in the early 2000s according to the SInAS database. Within the past 20 years, five downy mildew pathogens with the potential to cause significant losses have been introduced into Europe (Thines, 2011; Gilardi *et al*., 2013; Voglmayr, Montes‐Borrego & Landa, 2014; Görg *et al*., 2017; Thines *et al*., 2020) with seed or latently infected plants. In total, 373 alien species of SAR have been reported for Europe & Central Asia (Table 7). The majority belong to Ochrophyta (140 species), followed by Myzozoa (87), Foraminifera (70) and Oomycota (59). Alien species widespread throughout the IPBES region include *Aphanomyces astaci* (crayfish plague), *Colpomenia peregrina* (oyster thief), *Prorocentrum cordatum* (dinoflagellate), and *Sargassum muticum* (brown alga). The highest numbers of alien SAR have been reported from France (72 species), followed by Ukraine (61), Turkey (58), and Germany (38) (see also Çinar *et al*., 2005; Rabitsch & Nehring, 2017). For several regions, comprehensive lists of alien species have been published, which also include SAR; for example, Austria (43 oomycete species; Voglmayr *et al*., 2023), France (36 oomycete species; Desprez‐Loustau *et al*., 2010), Norway (18 species; Sandvik *et al*., 2020), Switzerland (36 oomycete species; Beenken & Senn‐Irlet, 2016), the UK (13 species; Roy *et al*., 2012), and the Mediterranean Sea (85 species, Galanidi *et al*., 2023). The majority of SAR are marine. Europe has a well‐documented history of invasions for certain alien pathogenic oomycetes, such as *A. astaci* (crayfish plague; Mrugała *et al*., 2015), *Phytophthora infestans* that triggered the Irish Great Famine in the 1840s (potato blight; Yoshida *et al*., 2013), *Phytophthora x alni* (alder dieback; Jung *et al*., 2018), and *Plasmopara viticola* (grapevine downy mildew), which was introduced in 1878 (Gessler, Pertot & Perazzolli, 2011). One of the few records of alien SAR from Central Asia is from Georgia with a collection of marine species (Editorial Board of AquaNIS, 2024).

**Table 7:** Numbers of established alien species for subregions of Europe & Central Asia. The numbers are extracted from the SInAS database (see Section II) and may deviate from those reported in regional studies. The numbers should be considered as minimum values as the true level of invasion is likely much higher. Empty cells may reflect a lack of research. For mammals, birds, and vascular plants ranges of values indicate variation among databases. Duplicated taxa for the same region and Totals were removed.

	Central and Western Europe	Central Asia	Eastern Europe	Totals
Mammals	64–133	5–23	24–80	72–164
Birds	218–627	4–5	20–24	221–630
Fishes	423	51	119	469
Reptiles	94		6	98
Amphibians	42	2	5	43
Insects	2698	28	213	2747
Arachnids	289	2	6	289
Molluscs	557	4	75	584
Crustaceans	508	11	120	563
Vascular plants	4498–7896	134–361	1950–2400	5146–8519
Algae	210		5	212
Bryophytes	37		1	37
Fungi	594	3	28	609
SAR	332		79	373
Bacteria and protozoans	22		2	23
Totals	10,586–14,462	244–490	2653–3163	11,486–15,360

### (3) Fungi

In Europe & Central Asia, 609 alien fungi have been reported (Table 7). Nearly all records are from Europe with only a few from Central Asia: five alien fungi in Georgia, two in Kazakhstan, and one in Azerbaijan and Uzbekistan. *Ophiostoma novo‐ulmi* (Dutch elm disease) has been reported from all of these countries (Brasier, 1991; Brasier & Kirk, 2001). In addition to the impact of *B. dendrobatidis* on many amphibians, *Batrachochytrium salamandrivorans* causing declines in *Salamandra salamandra* (salamander) populations has been recorded recently in Germany, Belgium, Spain, and the Netherlands and is likely to invade other areas, such as North America (Scheele *et al*., 2019; Castro Monzon *et al*., 2022).

Earliest first records of alien fungi date back to a report of the mediterranean *Clathrus ruber* (latticed stinkhorn) in 1752 in Germany (Monteiro *et al*., 2020) and the North American *Ustilago maydis* (corn smut) in 1760 in France (Kreisel & Scholler, 1994). *Cronartium ribicola* (white pine blister rust) originates from Eastern Asia and was recorded first in the mid‐19th century in Eastern Europe from where it spread to other European countries and North America (Richardson *et al*., 2009). The SInAS database reports a continuous increase in the number of new alien fungi species with a first peak of a mean of seven new alien fungi annually around 1900 and the highest numbers today (10 annually). Using dried reference‐collection samples, Gross *et al*. (2021) demonstrated that three species of *Erysiphe* could be linked to the incidence of powdery mildew in oaks, a disease that emerged in Europe at the beginning of the 20th century. One century earlier, in 1845, powdery mildew of grapevine (*Eryspihe necator*) was introduced from North America to England, a story analogous to that of the fungus‐like grapevine downy mildew (Gadoury *et al*., 2012).

The highest numbers of alien fungi have been published for Austria (323; Voglmayr *et al*., 2023), Switzerland (247; Beenken & Senn‐Irlet, 2016), Slovenia (216; de Groot *et al*., 2020), and France (191; Desprez‐Loustau *et al*., 2010); mostly a result of high research efforts or national assessments. Those studies show steep increases in the number of newly recorded alien fungi particularly since 1900. A similar increase until today was found in an assessment of 79 alien fungi in Norway (Sandvik *et al*., 2019). For the UK, 157 alien fungi were recorded and the number of new alien fungi did not show a clear trend since 1970 with *ca*. 15–20 new species per five‐year interval (Jones & Baker, 2007). The highest numbers of invasive forest pathogenic fungi are reported from the central part of Europe (France, Italy, and Switzerland; Santini *et al*., 2013); most are native to the Northern Hemisphere, but about one‐third are of unknown origin (Desprez‐Loustau, 2009). Some of the most widespread are *Hymenoscyphus fraxineus* (ash dieback) and *Ophiostoma novo‐ulmi* (Dutch elm disease), which caused severe diebacks in ash and elm, respectively, throughout Europe. The number of alien powdery mildews (Erysiphales) in Europe is rather high (Desprez‐Loustau *et al*., 2010; Beenken & Senn‐Irlet, 2016; Voglmayr *et al*., 2023) and may reflect responses to climate change in a group adapted for long‐distance aerial spore dispersal (Heluta *et al*., 2009).

### (4) Plantae

#### (a) Historical trends

**Fig. 7:**
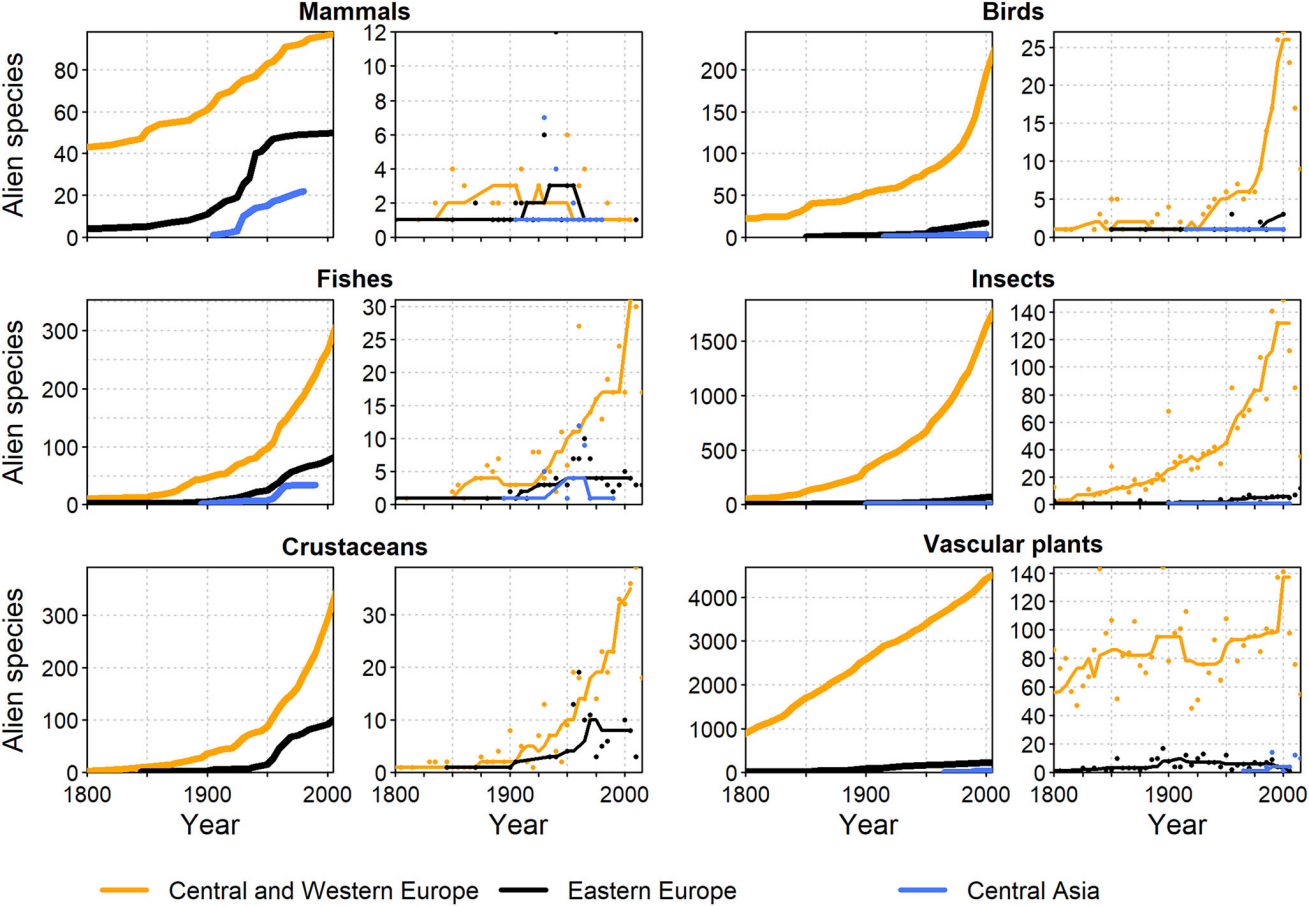
Trends in numbers of established alien species in Europe & Central Asia. Panels by taxon show cumulative numbers (left panels, thick lines) and number of new alien species per five‐year intervals (right panels, thin lines). Numbers shown here underestimate the actual extent of alien species occurrences due to a lack of data. Lines in right panels indicate smoothed trends calculated as running medians. Note that numbers presented may deviate from those reported in the text due to variation among data sources.

Several studies have reported long‐term increasing trends of alien plants for individual countries, such as Estonia (Ööpik *et al*., 2008), Albania (Barina *et al*., 2014), Italy (Celesti‐Grapow *et al*., 2009), Turkey (Çinar *et al*., 2005), Slovakia (Medvecká *et al*., 2012), Poland (Tokarska‐Guzik, 2005), Czech Republic (Pyšek *et al*., 2012a), Ukraine (Protopopova & Shevera, 2014), Iceland (Wasowicz, Przedpelska‐Wasowicz & Kristinsson, 2013), and Portugal (Almeida & Freitas, 2012). The reported temporal patterns are very similar across regions with a distinct acceleration of new plant records either during the early (e.g. Pyšek *et al*., 2012a) or late 19th century (e.g. Wasowicz *et al*., 2013). The first comprehensive study of alien plant invasions across European countries showed a slow increase of new introductions until 1800 and a marked acceleration thereafter (Lambdon *et al*., 2008). Whereas before 1800 the majority of alien plants were of European origin, this changed during the 19th century with the large majority of alien plants now being of non‐European origin (Lambdon *et al*., 2008). In addition to cumulative numbers, the rate of new alien plant records increased from 1800 until today as well (Fig. 7), reaching around 20 new alien species records annually in Europe (Seebens *et al*., 2017). Information about alien plant introductions in Central Asia is scarce (Lazkov & Sultanova, 2011) but it seems likely that trends resemble those of neighbouring regions in Europe and other Asian regions, such as Nepal (Shrestha, 2016) or Siberia (Vinogradov & Kupriyanov, 2016), albeit at different levels.

Similarly, numbers of alien bryophytes have increased continuously, starting with the first observed species introduced to Europe, *Lunularia cruciata*, in 1828 (Essl & Lambdon, 2009). First records for alien ferns indicate similar trends although the available data are too scant to reveal robust trends (Jones *et al*., 2019). The introduction of alien aquatic plants increased after 1950, with the trade in ornamentals being the main pathway, followed by cultivation and contaminants of commodities; the former two exhibited similar rates in different European areas, while contaminants of commodities were mostly recorded in Southern Europe (Nunes *et al*., 2015). The number of alien aquatic plant species is still relatively low in European freshwaters but sharply increasing and has e.g. doubled in ~30 years in Germany (Hussner *et al*., 2010).

#### (b) Current status

The most recent and most comprehensive survey reports 4139 alien vascular plants in Europe & Central Asia (van Kleunen *et al*., 2019). According to this survey, the highest numbers have been recorded in England (1379), Sweden (874), Scotland (861), Wales (835), France (716), and Norway (595) (Pyšek *et al*., 2017), indicating that the Northern part of the continent, particularly the UK and Scandinavia, is heavily invaded by alien plants (Lambdon *et al*., 2008). Only a few regions in Eastern Europe harbour comparably high numbers of alien species, such as the European part of Russia (649), Ukraine (626), and Bulgaria (593). In some countries, such as the UK, Sweden, and Norway, alien species make up 32–47% of the total flora (Pyšek *et al*., 2017). Central Asia is generally less invaded by alien plants, with country floras in this region harbouring 50–70 alien species, corresponding to a 1.9–4.5% contribution to the total plant diversity (Pyšek *et al*., 2017). In some cases, this paucity reflects environmental conditions that are unfavourable for invasion. In Mongolia, a recent study recorded 154 taxa of alien plants, of which 33 are established and 121 casual; the low number of established plants is attributed to harsh conditions and historic isolation of this country (Vanjil *et al*., 2024). In Uzbekistan, intensive long‐term research yielded 242 alien plant species of which 211 are established, and 36 of these are invasive (Makhkamov *et al*., 2024). However, generally low alien species numbers in Central Asia can also be due to low research effort. Thirty‐five alien species have become established in more than 30 regions of Europe, representing at least half of this continent's territory, the most widespread being *Erigeron canadensis* (Pyšek *et al*., 2017). Some of the best‐studied plant invasions in Europe include the terrestrial herbs *Heracleum mantegazzianum* (giant hogweed) (Pyšek *et al*., 2017; Shackleton *et al*., 2020), *Fallopia* sp. div. (knotweed) (Bailey & Conolly, 2000), *Carpobrotus edulis* (Hottentot‐fig) (Novoa *et al*., 2013), *Senecio inaequidens* (narrow‐leaf ragwort) (Heger & Boehmer, 2005), the aquatic herbs *Elodea canadensis* (American duckweed), and the trees *Robinia pseudoacacia* (black locust), *Acer negundo* (ash‐leaf maple), and *Acacia dealbata* (silver wattle) (Nentwig *et al*., 2018).

There are 210 alien plants recorded in European freshwaters, mostly originating from North America and Asia (Nunes *et al*., 2015). *Elodea canadensis* is the most widely distributed alien aquatic plant in Europe, occurring in 41 European countries, followed by *Azolla filiculoides* (water fern) (25), and *Vallisneria spiralis* (tape grass) (22). Some of the established aquatic species are widespread and have increasing negative impacts across Europe, such as *Elodea* spp. (waterweeds), *Hydrocotyle ranunculoides* (floating pennywort), and *Myriophyllum aquaticum* (parrot feather watermilfoil) (Hussner, 2012).

Among alien bryophytes, 32 identified alien species in Europe comprise 21 mosses, 11 liverworts but no hornworts. The countries with the most alien bryophytes are the UK (14 species) and Ireland (six species). Overall, countries and regions with a humid and cool climate are most invaded, whereas countries with drier and warmer climates are poor in alien bryophytes (Essl & Lambdon, 2009). *Campylopus introflexus* (present in 21 countries) and *Orthodontium lineare* (15 countries) are the most widely distributed alien bryophytes in Europe (Essl *et al*., 2014).

Galanidi *et al*. (2023) list 110 marine alien plants from the Mediterranean Sea. The major putative pathway of introductions to the Northwestern Basin and the Adriatic Sea is shellfish transfer, and to the Eastern and Central Mediterranean, the Suez Canal. Well‐known and widespread seaweeds are *Caulerpa taxifolia* (killer alga), *C. cylindracea* (grape caulerpa), *Codium fragile* (dead man's finger), the invasive strain of *Asparagopsis taxiformis*, and *Womersleyella setacea*. *Halophila stipulacea* (broadleaf seagrass), the sole alien seagrass species in the Mediterranean, was first recorded in 1894, a couple of decades after the opening of the Suez Canal (Fritsch, 1895). It has since spread across the Mediterranean. In the 2000s, it spread to the Caribbean (Winters *et al*., 2020).

### (5) Animalia

#### (a) Historical trends

The number of alien animal species in Europe & Central Asia is increasing across various groups, including vertebrates (Rabitsch & Nehring, 2017), insects (Roques *et al*., 2016), molluscs (Peltanová *et al*., 2012), and freshwater animals (Nunes *et al*., 2015; Muñoz‐Mas & García‐Berthou, 2020), especially for Central and Western Europe (Fig. 7). The number of alien mammals introduced to European countries accelerated particularly in the late 19th century and continues to increase (Genovesi *et al*., 2012). The rates of new records of alien species remained low over the last 200 years, but rose sharply in recent decades for birds and invertebrates (Seebens *et al*., 2017). Amphibians, reptiles, and mammals share a similar pattern: historical events and trade routes around the Mediterranean Basin have resulted in some of the oldest known introductions of vertebrates in the world. The observed increases in alien species numbers will likely continue (Seebens *et al*., 2017, 2021a), and the pet trade is expected to contribute more species in the near and medium future (Pleguezuelos, 2002; Mateo, Ayres & López‐Jurado, 2011).

Introductions of alien freshwater fishes increased after the mid‐19th century due to the activities of acclimatisation societies, mainly for angling (Gherardi *et al*., 2009), and again after World War II due to more intensive trade, openings of major inland canals and waterways, and the intensification of aquaculture (Goren & Ortal, 1999; Gherardi *et al*., 2009; Nunes *et al*., 2015). In Central and Northern Europe, interconnected canals and waterways were the main pathways of introduction for aquatic invertebrates, while introductions of vertebrates were mainly by releases and escapes linked to aquaculture and pet and aquarium trades. A slight decrease in introduction rates was reported for recent decades on the Iberian Peninsula (Muñoz‐Mas & García‐Berthou, 2020). Three North American crayfish were intentionally introduced following the decimation of native crayfish populations: *Faxonius limosus* (spiny‐cheek crayfish) in 1890s, *Pacifastacus leniusculus* (signal crayfish) in 1960s, and *Procambarus clarkii* (red swamp crayfish) in the 1970s. These species are ubiquitous and currently spreading in Central and Eastern Europe (Soto *et al*., 2023). At least five North American species (genera *Faxonius* and *Procambarus*) have been subsequently documented. The fastest spreading crayfish is *Procambarus virginalis* (marbled crayfish), a parthenogenetic species previously related to *P. fallax*. Its fast growth, high fecundity, frequent spawning, short embryogenesis and environmental plasticity has enabled it to establish self‐sustaining populations from Estonia to Israel within two decades of its first population in the wild (Kouba, Petrusek & Kozák, 2014; Aluma *et al*., 2023; Carneiro, Galil & Lyko, 2023).

Across European Seas, the number of recorded alien species has increased continuously until today (Galil *et al*., 2014; Zenetos *et al*., 2022). In the North Sea, the annual introduction rate has more than doubled, from 2.9 species per year in 1950–1999 to 4.3 between 2000 and 2014, and 7 between 2015 and 2022 (ICES, 2022), whereas along the coast of Israel their number has tripled between 1970 (138 alien animal species) and 2020 (432 alien animal species), nearly 6 species per year (Galil *et al*., 2021).

#### (b) Current status

Currently, 85 alien mammals are known to be established in Europe & Central Asia, particularly in Central Western Europe, numerous Mediterranean islands, the British Isles, Italy, Scandinavia, Eastern Europe, and European Russia (Biancolini *et al*., 2021). *Ondatra zibethicus* (muskrat), *Nyctereutes procyonoides* (raccoon dog), and *Neovison vison* (American mink) are among the most widespread (Genovesi *et al*., 2012; Biancolini *et al*., 2021; Tedeschi *et al*., 2022). Many islands in European Seas have been invaded by large numbers of alien mammals for centuries (Bonesi & Palazon, 2007; Ruffino *et al*., 2009; Chainho *et al*., 2015; Capizzi, 2020). Europe has also been a hotspot of alien bird introductions for centuries, with highest numbers recorded in the UK, Spain, and Portugal (Dyer *et al*., 2017a). Although European‐Union‐wide import bans on caged birds, established after the bird flu epidemic of 2005, have greatly restricted the bird trade with other continents (Reino *et al*., 2017), there is still an extensive within‐Europe trade of captive‐bred birds that can potentially lead to new introductions. Europe hosts several global hotspots of alien amphibians and reptiles: the Balearic Islands, mainland Spain, Italy, France, and the UK (Ficetola *et al*., 2007; Kark *et al*., 2009; Kraus, 2009; Mateo *et al*., 2011; Capinha *et al*., 2017). Fewer alien reptiles and amphibians have been reported in Central Asian countries than in Europe (Kraus, 2009; Capinha *et al*., 2017). Numbers of alien insects have steadily increased in Europe but this accumulation lagged behind other regions, such as The Americas prior to 1900 (Figs 5 and 7). Historical patterns of insect species spread with Europe have been influenced by changes in governmental alliances and associated changes in trade (Roques *et al*., 2016).

There have been 534 alien animal freshwater species recorded in Europe & Central Asia, with the Iberian Peninsula, France, Italy, the UK, and Germany harbouring the highest numbers (Nunes *et al*., 2015). The most numerous known introduced organisms are fishes, arriving through stocking, aquaculture, or pet and aquarium trades, followed by crustaceans and molluscs, introduced mainly *via* ornamental trade and through canals and waterways (Nunes *et al*., 2015). Fish, such as *Cyprinus carpio* (common carp), *Sander lucioperca* (pike perch), *Silurus glanis* (European catfish), or Ponto‐Caspian gobies, have now established in much of European fresh waters (Leprieur *et al*., 2008) and threaten native fish faunas with a high level of endemism (Clavero & García‐Berthou, 2006). Data from Central Asia are scarce. At least, 31 alien freshwater fishes have been recorded in Uzbekistan (Yuldashov, 2018). Many freshwater and brackish water invertebrates, such as *Dreissena polymorpha* (zebra mussel), *D. bugensis* (quagga mussel), and many amphipods, such as *Dikerogammarus villosus* (killer shrimp), reached Europe from their Ponto‐Caspian native range through human‐made canals built for transport (Bij de Vaate *et al*., 2002). Other Asian crustaceans were introduced through rice cultivation, which brought at least 13 ostracods to Mediterranean wetlands (Bisquert‐Ribes *et al*., 2023). The Chinese pond mussel (*Sinanodonta woodiana*) has been introduced through the introduction of fish, which were infected by the parasitic larvae of the mussel (Douda *et al*., 2025).

The European Union (EU) has encouraged its member states to assess the status of marine alien species as part of the Marine Strategy Framework Directive (MSFD). Yet, there is no scientifically validated current inventory of marine alien animals recorded across European coastal and adjacent waters, and data are currently scattered among national and regional reports and databases. Several studies are available for individual countries, reporting numbers of marine alien animal species off, for example, Finland (27; Outinen *et al*., 2024), Denmark (55; Jensen *et al*., 2023), Republic of Ireland (65; Gittenberger *et al*., 2023a), the Netherlands (133; Gittenberger *et al*., 2023b), and the Atlantic coast of Spain (89; Png‐Gonzalez *et al*., 2023). The number of marine alien animal species recorded in the Mediterranean Sea (where only 8 of 22 countries are EU member states) is 810 (of a total of 1006 alien species) (Galanidi *et al*., 2023), of which about 75% have been introduced through the Suez Canal (Galil *et al*., 2021). Examples of marine invasions are the expansion of *Lagocephalus sceleratus* (silver‐cheeked toadfish) throughout the Mediterranean, an avid predator introduced through the Suez Canal (Ulman *et al*., 2024), the spread of *Neogobius melanostomus* (round goby), a Ponto‐Caspian introduction into the Baltic Sea (Kornis, Mercado‐Silva & Vander Zanden, 2012), and the introduction of the comb jellyfish *Mnemiopsis leidyi* (sea walnut) into the Black Sea (Oguz, Fach & Salihoglu, 2008).

## IX. ANTARCTICA

Antarctica has been much less affected by alien species than other regions, for several reasons: Antarctica is difficult to access, anthropogenic pressures have been low so far (Bennett, 2015; McGeoch *et al*., 2015; Galera *et al*., 2018), and its inhospitable environments, such as low nutrient soils, freezing temperatures, and high ultraviolet (UV) levels, do not favour establishment of alien species. However, climate change and increased human activities through tourism and research are enhancing introductions (Bender, Crosbie & Lynch, 2016; Duffy *et al*., 2017; Bartlett, Convey & Hayward, 2020; Chwedorzewska, Korczak‐Abshire & Znój, 2020). Plants (seeds, fragments, and other propagules) and invertebrates, such as springtails, were introduced on clothing and personal equipment of tourists and national Antarctic programme ship and aircraft personnel, as well as associated with packing materials (Chown *et al*., 2012; Huiskes *et al*., 2014), vehicles (Hughes *et al*., 2010), and fresh food imports (Hughes *et al*., 2011). In 11 years of surveillance (2006–2017) at the Scott Base in the Ross Sea region of continental Antarctica, 68 invertebrate species (16 of which are known to be invasive elsewhere globally, including in some instances the broader Antarctic region) were intercepted on food (60%), clothing and equipment (11%), aircraft and cargo (11%), and packaging material (11%) (Newman *et al*., 2018). During 2007–2008, more than 20 alien lichens and fungi were intercepted in Antarctica in packaging, foodstuffs, and timber (Osyczka, 2010; Osyczka *et al*., 2012). Seeds of eight alien plant species were reported in the topsoil of Fildes Peninsula, King George Island (Antarctica), in areas intensively frequented by humans (Fuentes‐Lillo *et al*., 2017).

Terrestrial alien plants in the Antarctic are predominantly herbs, mostly introduced unintentionally with soils or imported fodder for domestic animals (Frenot *et al*., 2005; Chwedorzewska *et al*., 2015). Alien plants have been introduced on several occasions since the 1950s: for example, *Poa pratensis* (smooth meadow‐grass) was introduced unintentionally during tree transplantation experiments in the 1950s and eradicated in 2015 (Pertierra *et al*., 2017). Altogether, 15 alien species are known to occur in Antarctica: one plant, *Poa annua* (annual bluegrass), and 14 invertebrates [seven Collembola, four Arachnida, two Insecta (Diptera), one Annelida], most of which are found in the Antarctic Peninsula region (Hughes, Cowan & Wilmotte, 2015; Baird *et al*., 2019; Enríquez *et al*., 2019, Hughes *et al*., 2020). This could be due to several factors. The Antarctic Peninsula is the area closest to another continent (South America), it is the least climatically extreme region of Antarctica (and has also experienced a rapid rise in temperatures since the 1950s due to climate change) and has the largest concentration of human activity (due to research teams and tourism) resulting in a relatively high propagule pressure (Hughes *et al*., 2020). On the sub‐Antarctic islands, which circle the continent, at least 108 alien plants, 72 terrestrial invertebrates, and 16 vertebrates are reported (Frenot *et al*., 2005).

Alien vertebrates with established populations are reported only for sub‐Antarctic islands. Environmental conditions on the continent of Antarctica itself are too extreme unless the species can live synanthropically: some mammals, such as rats and mice, were unintentionally introduced as early as the 18th century, while others, such as ungulates, cats, rabbits, and salmonids, were intentionally introduced beginning in the early 20th century (Frenot *et al*., 2005; Lecomte *et al*., 2013). Alien invertebrates, such as the springtail *Hypogastrura viatica*, were reported from the 1940s onwards in the Antarctic (Hack, 1949; Hughes *et al*., 2015).

To date, five marine alien invertebrate species have been found (plus one cryptogenic seaweed species); these were free‐living specimens with no known established populations (McCarthy *et al*., 2019; Cárdenas *et al*., 2020). Marine alien species were likely introduced by vessels (three by hull fouling, one by ballast water), with the first recorded alien species (a bryozoan) dating back to 1960, followed in 1986 by a crab, and in 1996 by a tunicate and a hydroid; the most recent introduction (a mollusc) was recorded in 2019, although it is likely that this species has subsequently disappeared (McCarthy *et al*., 2019; Cárdenas *et al*., 2020). There is no evidence that any of these species are established in Antarctica (McCarthy *et al*., 2019).

The number of alien species is expected to increase in the future due to climate change and increasing human pressure, but reported numbers are also expected to be higher due to the greater research effort, as noted by the growing number of relevant publications (Hughes & Pertierra, 2016; Duffy *et al*., 2017; Ricciardi *et al*., 2017; Chan *et al*., 2019; Chwedorzewska *et al*., 2020). A recent horizon scan for future potentially invasive alien species in the Antarctic Peninsula underlined the main threat posed by marine invertebrates that can be unintentionally transported in ballast waters and on ship hulls (McCarthy *et al*., 2019; Hughes *et al*., 2020). The threat could be even greater considering the cruise ship volume from the Northern Hemisphere to Antarctica that may increase the probability of introduction.

## X. FUTURE TRENDS OF BIOLOGICAL INVASIONS

Many studies have been conducted to explore the potential future developments of the distribution and accumulation of alien species at various geographic scales. Across eight continental regions, alien species numbers for seven major taxonomic groups are projected to increase on average by 36% until 2050 under a business‐as‐usual scenario (Seebens *et al*., 2021a). Taxon‐specific studies at global or regional scales about future invasion potential have been conducted for different individual species or taxonomic groups, such as ants and termites (Chen, 2008; Buczkowski & Bertelsmeier, 2017), beetles (Berzitis *et al*., 2014; Wang *et al*., 2017), flies (Hill *et al*., 2016; Ryan *et al*., 2019), other insects (Hill, Gallardo & Terblanche, 2017; Lu *et al*., 2020), amphibians (Ihlow *et al*., 2016; Forti *et al*., 2017), fish (Liu *et al*., 2019; Dong *et al*., 2020), and mammals (Louppe *et al*., 2019, 2020; Biancolini *et al*., 2024), mostly projecting an increase in range sizes. Species distribution models for 100 alien species with severe impacts (as assessed by the IUCN) found a decreased potential for future global distribution of mammals, birds, fishes, reptiles, and amphibians, but an increase in distributions of aquatic and terrestrial invertebrates due to region‐specific projected changes in climate (Bellard *et al*., 2013).

Species‐specific studies about future distributions of alien microorganisms are available only for a few species and the majority deal with just one species or multiple species from one genus (e.g. *Phytophthora*; Scott *et al*., 2019). In general, invasion potentials are projected to be higher than currently observed, both in terms of numbers of alien fungi present (Bebber *et al*., 2019; Barwell *et al*., 2021) and of occupied range (Kriticos *et al*., 2013; Feldmeier *et al*., 2016). For crop pests including herbivorous arthropods, pathogenic microbes and viruses, numbers within regions are projected to be higher than observed levels (Bebber *et al*., 2019). Hotspots of pest invasion are in Central America, Europe, East Asia, and Australia (Bebber, 2015). Global plant pathogen studies project an increase in potentially suitable areas, especially towards higher latitudes (Burgess *et al*., 2017; Avila *et al*., 2019). Crop pests are projected to shift poleward under climate change and increased human activities (Fisher *et al*., 2012; Bebber, Ramotowski & Gurr, 2013) and under currently observed trends, the main crop‐producing countries will be saturated with crop pathogens by 2050 (Bebber *et al*., 2014).

Future hotspots for alien plant invasions have been identified in Europe, South America, North America, Southwest China, and New Zealand as well as the coast of West Africa and the Southern coast of Asia (Wan, Wang & Yu, 2016), while potential hotspots for cacti emerge in the Mediterranean, tropical savanna regions, and xeric shrubland biomes (Masocha & Dube, 2018). Most projections of future distributions of alien plant species identified expanding ranges (Adhikari, Tiwari & Barik, 2015; Wan *et al*., 2016; Dullinger *et al*., 2017), although for some species ranges are projected to shrink (Bellard *et al*., 2013). Studies for the USA and Europe suggest that most current invasion hotspots will remain spatially stable, but potential alien species numbers will increase by 64–102% (Allen & Bradley, 2016). For Europe, increases of alien plant numbers were projected for northern parts, while southern parts may experience declining numbers (Chytrý *et al*., 2012). South American countries, such as Brazil, Mexico, and Argentina, are expected to face comparatively strong increases in numbers of alien plant species based on global trade dynamics and climate change (Seebens *et al*., 2015), which may invert the current status of North America as more invaded by plants than South America (Pyšek *et al*., 2019).

Studies of alien animals often found projected expansions of current distributions. For example, for birds *Corvus splendens* (house crow) and *Acridotheres tristis* (common myna), the current distributions indicate a large potential to spread to new areas (Nyári, Ryall & Peterson, 2006; Magory Cohen *et al*., 2019). Similarly, mammals, such as *Sus scrofa* (feral pig), *Urva auropunctata* (small Indian mongoose), and *Procyon lotor* (raccoon), often have a large potential for further spread worldwide (Lewis *et al*., 2017; Louppe *et al*., 2019, 2020; Biancolini *et al*., 2024). For insects, several studies investigated the invasion potential of agricultural pest species (Kroschel *et al*., 2013; Kriticos *et al*., 2017; Marchioro & Krechemer, 2018), with the *Spodoptera frugiperda* (fall armyworm) (Early *et al*., 2018) and *Drosophila suzukii* (spotted wing drosophila) being prominent examples (dos Santos *et al*., 2017), and all studies found a high risk of invasion beyond the current realised distribution. The ranges of many problematic invertebrate invaders other than insects have also been projected to expand, such as the harmful freshwater bivalves *Dreissena* (zebra and quagga mussels), *Limnoperna* (golden mussel), and *Corbicula* (basket clams) (Gama *et al*., 2016; Petsch *et al*., 2021). In the marine realm, a study of 19 ascidian species found a large invasion potential especially at higher latitudes (Lins *et al*., 2018). Projections of planktonic and benthic species, as well as algae, suggest that under climate change scenarios the probability of establishment of alien species will increase at higher latitude (Seebens *et al*., 2016; Goldsmit *et al*., 2020).

In summary, the suite of studies available for projections of future dynamics of alien species suggests that overall ranges of alien species are expected to increase in most cases although with large variation due to a continuous introduction of new individuals and an expansion of ranges. In addition, ranges are expected to shift poleward as a consequence of global warming (Walther *et al*., 2009). However, projections of future dynamics of alien and invasive alien species are severely limited by (*i*) data availability of past and current distributions of species, (*ii*) limits in understanding of causal relationships between species occurrences, environmental changes, drivers of biological invasions, and the roles of dispersal, biotic interactions, and impacts caused by invasive alien species, (*iii*) lack of models to project future dynamics of biological invasions robustly, and (*iv*) the lack of scenarios covering a range of plausible future dynamics of drivers, which would allow exploring future trends under different scenarios. While models and scenarios can still be developed further, closing data gaps, particularly of historic distributions, is very difficult and even impossible in many cases.

## XI. WAYS FORWARD TO IMPROVE ASSESSMENTS ON BIOLOGICAL INVASIONS

Although we aimed to provide a balanced global overview for both regions and taxonomic coverage of species, we acknowledge that a complete global coverage across all regions and taxa remains elusive due to many data and knowledge gaps. The true extent of biological invasions is vastly underestimated, as large numbers of species are probably not yet identified as established in many regions. No checklist is likely to be complete, but particularly large gaps in data exist for most African countries and parts of Asia; for invertebrates, fungi, SAR, and Bacteria; and for freshwater and marine species compared to terrestrial species.

**Fig. 8:**
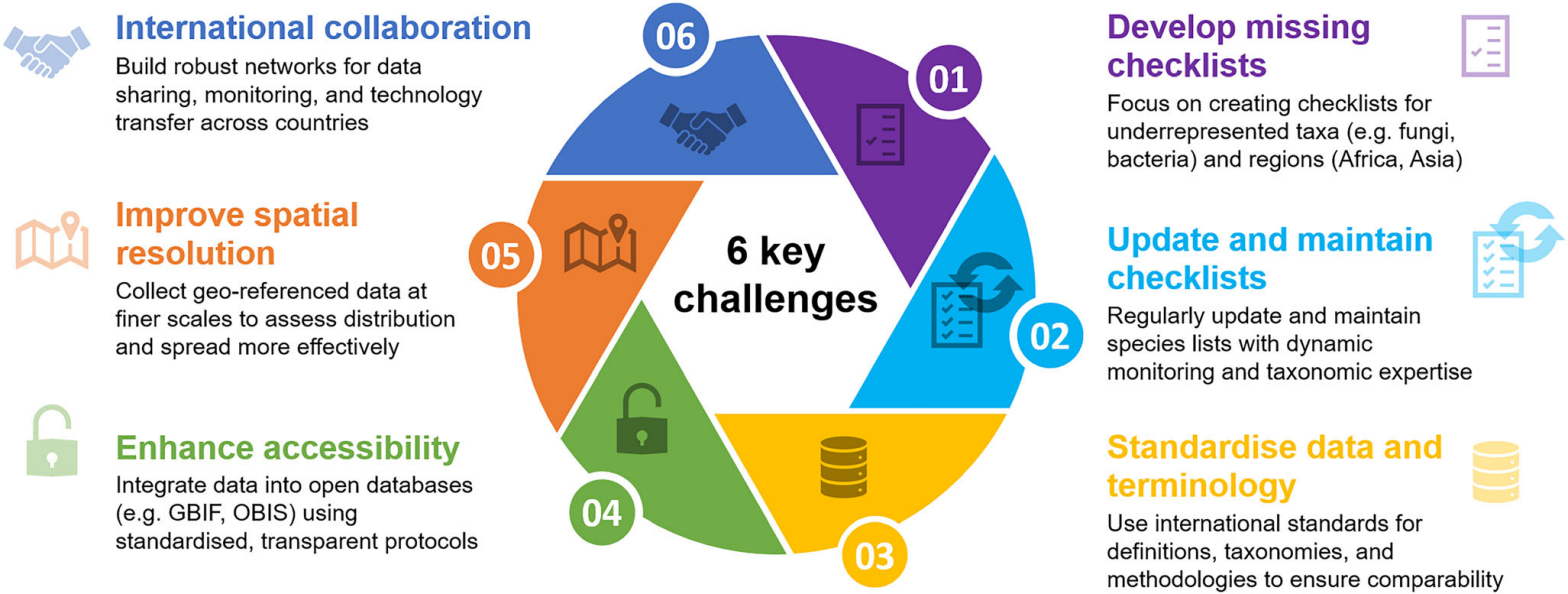
Six recommendations to improve the assessment of the status of the distribution of alien species. GBIF, Global Biodiversity Information Facility; OBIS, Ocean Biodiversity Information System.

To close the information gaps, we call for funding agencies, administrations, and scientists to collaborate to address the following key challenges (Fig. 8).

(1) *Checklists of alien species are lacking for many taxonomic groups and regions*. Checklists (i.e. species lists for individual regions) of alien species are missing for many taxonomic groups, particularly invertebrates, fungi, SAR, and prokaryotes, and for many countries in Africa and Asia. Developing such checklists is essential to enable more comprehensive and robust assessments of biological invasion trends.
(2) *Existing checklists of alien species are often incomplete and outdated*. We found substantial differences in alien species occurrences among neighbouring countries and across taxonomic groups. Such variations may arise from varying survey efforts and accessibility of data of alien species rather than actual occurrences. Because the spread of alien species is highly dynamic, maintaining up‐to‐date occurrence lists requires substantial investment in regular monitoring, taxonomic expertise, and continuous maintenance of databases and standards.
(3) *Available alien species lists lack standardisation*. Studies and reports often use different terms, definitions, concepts, taxonomies, data collection, and sampling methods making comparisons across regions and taxa challenging, particularly for distinguishing invasion status (i.e. introduced, established, and invasive). Such distinctions are often not specified, or if they are, definitions are often lacking (Wilson *et al*., 2020). Comparisons and assessments of biological invasions need international standards in monitoring and reporting (Latombe *et al*., 2017; Packer *et al*., 2017; Meyerson *et al*., 2022).
(4) *Data are often difficult to access or are inaccessible*. Information about alien species occurrences is often stored in formats that are inaccessible or challenging to access, such as appendices of articles or tables in pdf documents (Crall *et al*., 2006). Integration of standardised data into open databases or data portals, such as GBIF or the Ocean Biodiversity Information System (OBIS), would enable researchers and stakeholders to access information in a standardised way and to conduct tailored biodiversity assessments. Recording and storing data should follow standard and published protocols to make science, decision‐making, and the assessment of biodiversity comprehensive, transparent, interoperable, and reproducible, which ultimately increases trust in results and decisions (e.g. De Pooter *et al*., 2017; Groom *et al*., 2017; Roy *et al*., 2018; Haider *et al*., 2022).
(5) *Coarse spatial resolutions of occurrences impede assessments of distribution and spread*. Although checklists provide the basis for analysing status and trends of alien species, their spatial resolution is too coarse to enable a robust assessment of distributions and spread of alien species. This is particularly problematic for large countries, but introduces challenges for monitoring species even within smaller countries. Further, checklists are often restricted to administrative units, such as countries, although information at biogeographic (e.g. biomes, islands) or ecological (e.g. habitat types) levels would allow improved assessments. Accurate assessments of biological invasions across spatial scales require data at finer spatial resolutions that are ideally geo‐referenced. Only very few countries, such as the UK or Czech Republic, have updated information on alien species occurrences in the form of raster mapping (Preston, Pearman & Dines, 2002; Pyšek *et al*., 2022).
(6) *Addressing the challenge of biological invasions requires international collaboration*. Greater international collaboration is needed to build more robust global networks for monitoring, data sharing, and technology transfer (Packer *et al*., 2017; Nuñez *et al*., 2021; Kuebbing *et al*., 2022; Meyerson *et al*., 2022; Soubeyrand *et al*., 2024). While several research networks, database repositories, intergovernmental and international organisations, and international agreements related to biological invasions do exist (reviewed in Meyerson *et al*., 2022), additional coordination and collaboration are needed, particularly because individual countries often lack the capacities to respond appropriately to biological invasions (Early *et al*., 2016; Pyšek *et al*., 2020). Engaging in discussions based on a genuine interchange that recognises and incorporates differences in knowledge, values, perspectives, and interests across political boundaries is critical (Courchamp *et al*., 2017) to increase understanding of biological invasions and support data acquisition.

Thoroughly assessing the trends and status of biodiversity requires deep knowledge about nature and the ecosystems supporting biodiversity. Although information about nature is accumulating at an unprecedented pace, major knowledge gaps persist, particularly for inconspicuous organisms, such as invertebrates, fungi, SAR, and Bacteria, and less‐accessible systems, such as marine ecosystems and inland waters, and in geographic areas such as Central Africa, Central Asia, Antarctica, and several remote islands. Our current knowledge of species interactions with their environment is inadequate to understand comprehensively how species respond to environmental changes in order to build sufficient models to anticipate future biodiversity change under different scenarios of human development and climate change. Reducing these knowledge gaps is therefore key to improve policies that can safeguard nature and move societies towards sustainability. New technologies ranging from satellite products, automated sampling, citizen science and environmental DNA (eDNA) will help in gathering information and reducing gaps. Still, knowledge gaps will persist and must be considered explicitly when assessing status and trends of biological invasions.

## XII. CONCLUSIONS

(1) Increasing amounts of data and new databases on various facets of biological invasions have become available recently. However, drawing robust conclusions from data also requires the assessment of data and knowledge biases and gaps by experts. To evaluate changes over time, such assessments and updates must be done regularly; this is essential for informing stakeholders and policymakers, and for guiding targeted management efforts.
(2) Every region on Earth has experienced introductions of alien species. We have reports of introductions and establishments of alien species even from the most remote and inhospitable places, often in surprisingly large quantities.
(3) The reported distribution of alien species worldwide is highly uneven. Many reports are from economically wealthy countries and far fewer are from countries of the Global South. It remains unclear how much collated distribution patterns reflect sampling effort rather than the ‘true’ distribution of alien species.
(4) Numbers of alien species are increasing for all taxonomic groups and regions, and this trend is likely to continue.
(5) Available information about numbers and distributions of alien species is often very incomplete. As a result, numbers and distributions shown in this review represent (sometimes severe) under‐estimates. Although we can draw robust conclusions about the general trends of biological invasions for many regions and taxonomic groups, much more effort and data are needed to facilitate more thorough assessments of the status and trends for all taxonomic groups and regions.
(6) We provide six recommendations to improve the situation of assessing status and trends of alien species. These cover priorities relating to data collation (including developing and applying standards), free and FAIR data provisioning, and concerted efforts across borders, among others.

## References

[brv70058-bib-0001] Abbott, I. , Marchant, N. & Cranfield, R. (2000). Long‐term change in the floristic composition and vegetation structure of Carnac Island, Western Australia. Journal of Biogeography 27, 333–346.

[brv70058-bib-0002] Acevedo‐Rodríguez, P. & Strong, M. T. (2008). Floristic richness and affinities in the West Indies. The Botanical Review 74, 5–36.

[brv70058-bib-0003] Adhikari, D. , Tiwary, R. & Barik, S. K. (2015). Modelling hotspots for invasive alien plants in India. PLoS One 10, e0134665. 26230513 10.1371/journal.pone.0134665 PMC4521859

[brv70058-bib-0004] Akhtar, J. , Chalam, V. C. , Kumar, P. , Kiran, R. & Dubey, S. C. (2019). Plant quarantine – A phytosanitary requirement for disease free import of plant genetic resources in India. In Integrated Pest Management in Major Crops (eds J. Stanley , K. K. Mishra , A. R. N. S. Subbanna , H. Rajashekara and A. Pattnayak ), pp. 153–163. ICAR‐Vivekananda Parvatiya Krishi Anusandhan Sansthan, Almora.

[brv70058-bib-0005] Akhtar, J. , Gupta, K. , Gawade, B. H. , Kumar, P. , Meena, B. R. , Kiran, R. & Chalam, V. C. (2021). Strategies to combat the threat of quarantine pests to the plant health and food security. In Technology Strides in Plant Health Management (eds N. K. Bharat and H. R. Guatam ), pp. 273–288. Neoti Book Agency Pvt. Ltd., New Delhi.

[brv70058-bib-0006] Allen, J. M. & Bradley, B. A. (2016). Out of the weeds? Reduced plant invasion risk with climate change in the continental United States. Biological Conservation 203, 306–312.

[brv70058-bib-0007] Almeida, J. D. & Freitas, H. (2012). Exotic flora of continental Portugal – a new assessment. Bocconea 24, 231–237.

[brv70058-bib-0008] Aluma, M. O. , Pukk, L. , Hurt, M. & Kaldre, K. (2023). Distribution of non‐indigenous crayfish species in Estonia and their impacts on noble crayfish (*Astacus astacus* L.) populations. Diversity 15, 474.

[brv70058-bib-0009] APASD (2024). Asian‐Pacific Alien Species Database (APASD). National Institute for Agro‐Environmental Sciences, Tsukuba. https://www.naro.affrc.go.jp/archive/niaes/techdoc/apasd/fungus.html.

[brv70058-bib-0010] Appleton, C. C. (2003). Alien and invasive freshwater Gastropoda in South Africa. African Journal of Aquatic Science 28, 69–81.

[brv70058-bib-0011] Appleton, C. C. & Brackenbury, T. D. (1998). Introduced freshwater gastropods in Africa with special reference to *Physa acuta* . In Proceedings of a Workshop on Medical Malacology in Africa, Harare, Zimbabwe, September 22–26, 1997 (eds H. Madsen , C. C. Appleton and M. Chimbari ), pp. 22–26. Danish Bilharziasis Laboratory, Charlottenlund.

[brv70058-bib-0012] Aukema, J. E. , McCullough, D. G. , Holle, B. V. , Liebhold, A. M. , Britton, K. & Frankel, S. J. (2010). Historical accumulation of nonindigenous forest pests in the continental United States. Bioscience 60, 886–897.

[brv70058-bib-0013] Auld, T. D. & Hutton, I. (2004). Conservation issues for the vascular flora of Lord Howe Island. Cunninghamia: a journal of plant ecology for eastern Australia 8, 490–500.

[brv70058-bib-0014] Aulus‐Giacosa, L. , Ollier, S. & Bertelsmeier, C. (2024). Non‐native ants are breaking down biogeographic boundaries and homogenizing community assemblages. Nature Communications 15, 2266. 10.1038/s41467-024-46359-9 PMC10937723 38480710

[brv70058-bib-0015] Avila, G. A. , Davidson, M. , van Helden, M. & Fagan, L. (2019). The potential distribution of the Russian wheat aphid (*Diuraphis noxia*): an updated distribution model including irrigation improves model fit for predicting potential spread. Bulletin of Entomological Research 109, 90–101. 29665868 10.1017/S0007485318000226

[brv70058-bib-0016] Bailey, S. A. , Brown, L. , Campbell, M. L. , Canning‐Clode, J. , Carlton, J. T. , Castro, N. , Chainho, P. , Chan, F. T. , Creed, J. C. , Curd, A. , Darling, J. , Fofonoff, P. , Galil, B. S. , Hewitt, C. L. , Inglis, G. J. , *et al*. (2020). Trends in the detection of aquatic non‐indigenous species across global marine, estuarine and freshwater ecosystems: a 50‐year perspective. Diversity and Distributions 26, 1780–1797. 36960319 10.1111/ddi.13167 PMC10031752

[brv70058-bib-0017] Bailey, J. P. & Conolly, A. P. (2000). Prize‐winners to pariahs – a history of Japanese knotweed s.l. (Polygonaceae) in the British Isles. Watsonia 23, 93–110.

[brv70058-bib-0018] Bailey, B. A. , Evans, H. C. , Phillips‐Mora, W. , Ali, S. S. & Meinhardt, L. W. (2018). *Moniliophthora roreri*, causal agent of cacao frosty pod rot. Molecular Plant Pathology 19, 1580–1594. 29194910 10.1111/mpp.12648 PMC6638017

[brv70058-bib-0019] Baird, H. P. , Janion‐Scheepers, C. , Stevens, M. I. , Leihy, R. I. & Chown, S. L. (2019). The ecological biogeography of indigenous and introduced Antarctic springtails. Journal of Biogeography 46, 1959–1973.

[brv70058-bib-0021] Baret, S. , Rouget, M. , Richardson, D. M. , Lavergne, C. , Egoh, B. , Dupont, J. & Strasberg, D. (2006). Current distribution and potential extent of the most invasive alien plant species on La Reunion (Indian Ocean, Mascarene islands). Austral Ecology 31, 747–758.

[brv70058-bib-0022] Barina, Z. , Rakaj, M. , Somogyi, G. , Erős‐Honti, Z. & Pifkó, D. (2014). The alien flora of Albania: history, current status and future trends. Weed Research 54, 196–215.

[brv70058-bib-0023] Barker, G. M. (1999). Naturalised terrestrial Stylommatophora (Mollusca: Gastropoda). Fauna of New Zealand 38, 1–253.

[brv70058-bib-0024] Bartlett, J. C. , Convey, P. & Hayward, S. A. L. (2020). Surviving the Antarctic winter—life stage cold tolerance and ice entrapment survival in the invasive chironomid midge *Eretmoptera murphyi* . Insects 11, 147. 32111052 10.3390/insects11030147 PMC7143863

[brv70058-bib-0025] Barwell, L. J. , Perez‐Sierra, A. , Henricot, B. , Harris, A. , Burgess, T. , Hardy, G. , Scott, P. , Williams, N. , Cooke, D. , Green, S. , Chapman, D. S. & Purse, B. V. (2020). *Phytophthora global impacts. (Version v1.0.0)*. Zenodo. 10.5281/zenodo.4081474. PMC8048555 33883780

[brv70058-bib-0026] Barwell, L. J. , Perez‐Sierra, A. , Henricot, B. , Harris, A. , Burgess, T. I. , Hardy, G. , Scott, P. , Williams, N. , Cooke, D. E. L. , Green, S. , Chapman, D. S. & Purse, B. V. (2021). Evolutionary trait‐based approaches for predicting future global impacts of plant pathogens in the genus *Phytophthora* . Journal of Applied Ecology 58, 718–730. 33883780 10.1111/1365-2664.13820 PMC8048555

[brv70058-bib-0027] Bebber, D. P. (2015). Range‐expanding pests and pathogens in a warming world. Annual Review of Phytopathology 53, 335–356. 10.1146/annurev-phyto-080614-120207 26047565

[brv70058-bib-0028] Bebber, D. P. , Field, E. , Gui, H. , Mortimer, P. , Holmes, T. & Gurr, S. J. (2019). Many unreported crop pests and pathogens are probably already present. Global Change Biology 25, 2703–2713. 31237022 10.1111/gcb.14698

[brv70058-bib-0029] Bebber, D. P. , Holmes, T. , Smith, D. & Gurr, S. J. (2014). Economic and physical determinants of the global distributions of crop pests and pathogens. New Phytologist 202, 901–910. 24517626 10.1111/nph.12722 PMC4285859

[brv70058-bib-0030] Bebber, D. P. , Ramotowski, M. A. T. & Gurr, S. J. (2013). Crop pests and pathogens move polewards in a warming world. Nature Climate Change 3, 985–988.

[brv70058-bib-0031] Beenken, L. & Senn‐Irlet, B. (2016). Neomyceten in der Schweiz. Stand des Wissens und Abschätzung des Schadpotentials der mit Pflanzen assoziierten gebietsfremden Pilze. WSL, Birmensdorf.

[brv70058-bib-0032] Bellard, C. , Bernery, C. & Leclerc, C. (2021). Looming extinctions due to invasive species: irreversible loss of ecological strategy and evolutionary history. Global Change Biology 27, 4967–4979. 34337834 10.1111/gcb.15771

[brv70058-bib-0033] Bellard, C. & Jeschke, J. M. (2016). A spatial mismatch between invader impacts and research publications. Conservation Biology 30, 230–232. 26308661 10.1111/cobi.12611

[brv70058-bib-0034] Bellard, C. , Thuiller, W. , Leroy, B. , Genovesi, P. , Bakkenes, M. & Courchamp, F. (2013). Will climate change promote future invasions? Global Change Biology 19, 3740–3748. 23913552 10.1111/gcb.12344 PMC3880863

[brv70058-bib-0035] Bender, N. A. , Crosbie, K. & Lynch, H. J. (2016). Patterns of tourism in the Antarctic peninsula region: a 20‐year analysis. Antarctic Science 28, 194–203.

[brv70058-bib-0036] Bennett, J. M. (2015). Agricultural big data: utilisation to discover the unknown and instigate practice change. Farm Policy Journal 12, 43–50.

[brv70058-bib-0037] Berg, C. (1877). Enumeración de las Plantas Européas que se Hallan Como Silvestres en la Provincia de Buenos Aires y en Patagonia. Impr. de Pablo E. Coni, Buenos Aires.

[brv70058-bib-0038] Berzitis, E. A. , Minigan, J. N. , Hallett, R. H. & Newman, J. A. (2014). Climate and host plant availability impact the future distribution of the bean leaf beetle (*Cerotoma trifurcata*). Global Change Biology 20, 2778–2792. 24616016 10.1111/gcb.12557

[brv70058-bib-0039] Bezerra, L. A. V. , Freitas, M. O. , Daga, V. S. , Occhi, T. V. T. , Faria, L. , Costa, A. P. L. , Padial, A. A. , Prodocimo, V. & Vitule, J. R. S. (2019). A network meta‐analysis of threats to South American fish biodiversity. Fish and Fisheries 20, 620–639.

[brv70058-bib-0040] Biancolini, D. , Pacifici, M. , Falaschi, M. , Bellard, C. , Blackburn, T. M. , Ficetola, G. F. & Rondinini, C. (2024). Global distribution of alien mammals under climate change. Global Change Biology 30, e17560. 39545282 10.1111/gcb.17560

[brv70058-bib-0041] Biancolini, D. , Vascellari, V. , Melone, B. , Blackburn, T. M. , Cassey, P. , Scrivens, S. L. & Rondinini, C. (2021). DAMA: the global distribution of alien mammals database. Ecology 102, e03474. 34273183 10.1002/ecy.3474

[brv70058-bib-0042] Bij de Vaate, A. , Jazdzewski, K. , Ketelaars, H. A. M. , Gollasch, S. & Van der Velde, G. (2002). Geographical patterns in range extension of Ponto‐Caspian macroinvertebrate species in Europe. Canadian Journal of Fisheries and Aquatic Sciences 59, 1159–1174.

[brv70058-bib-0043] Bisquert‐Ribes, M. , Horne, D. J. , Benavent, J. M. , Martínez, R. , Vera, P. , Rueda, J. & Mesquita‐Joanes, F. (2023). High incidence of exotic ostracods in the rice fields of a protected Mediterranean wetland. Inland Waters 13, 428–445.

[brv70058-bib-0044] Blackburn, T. M. , Bellard, C. & Ricciardi, A. (2019). Alien versus native species as drivers of recent extinctions. Frontiers in Ecology and the Environment 17, 203–207.

[brv70058-bib-0045] Blackburn, T. M. , Pyšek, P. , Bacher, S. , Carlton, J. T. , Duncan, R. P. , Jarošík, V. , Wilson, J. R. U. & Richardson, D. M. (2011). A proposed unified framework for biological invasions. Trends in Ecology & Evolution 26, 333–339. 21601306 10.1016/j.tree.2011.03.023

[brv70058-bib-0046] Bonesi, L. & Palazon, S. (2007). The American mink in Europe: status, impacts, and control. Biological Conservation 134, 470–483.

[brv70058-bib-0047] Borroto‐Páez, R. , Bosch, R. A. , Fabres, B. A. & García, O. A. (2015). Introduced amphibians and reptiles in the Cuban archipelago. Herpetological Conservation and Biology 10, 985–1012.

[brv70058-bib-0048] Bortolus, A. & Schwindt, E. (2022). Biological invasions and human dimensions: we still need to work hard on our social perspectives. Ecología Austral 32, 767–783.

[brv70058-bib-0049] Brandeis, T. J. , Helmer, E. H. , Marcano‐Vega, H. & Lugo, A. E. (2009). Climate shapes the novel plant communities that form after deforestation in Puerto Rico and the U.S. Virgin Islands. Forest Ecology and Management 258, 1704–1718.

[brv70058-bib-0050] Brandt, A. J. , Bellingham, P. J. , Duncan, R. P. , Etherington, T. R. , Fridley, J. D. , Howell, C. J. , Hulme, P. E. , Jo, I. , McGlone, M. S. , Richardson, S. J. , Sullivan, J. J. , Williams, P. A. & Peltzer, D. A. (2021). Naturalised plants transform the composition and function of the New Zealand flora. Biological Invasions 23, 351–366.

[brv70058-bib-0051] Brasier, C. M. (1991). *Ophiostoma novo‐ulmi* sp. nov., causative agent of current Dutch elm disease pandemics. Mycopathologia 115, 151–161.

[brv70058-bib-0052] Brasier, C. M. & Kirk, S. A. (2001). Designation of the EAN and NAN races of *Ophiostoma novo‐ulmi* as subspecies. Mycological Research 105, 547–554.

[brv70058-bib-0053] Brenton‐Rule, E. C. , Barbieri, R. F. & Lester, P. J. (2016). Corruption, development and governance indicators predict invasive species risk from trade. Proceedings of the Royal Society B: Biological Sciences 283, 20160901. 10.1098/rspb.2016.0901 PMC4920327 27306055

[brv70058-bib-0054] Brooks, E. G. E. , Allen, D. J. & Darwall, W. R. T. (2011). The Status and Distribution of Freshwater Biodiversity in Central Africa. Gland: IUCN (International Union for Conservation of Nature and Natural Resources), https://portals.iucn.org/library/node/9825.

[brv70058-bib-0055] Brown, C. J. , Blossey, B. , Maerz, J. C. & Joule, S. J. (2006). Invasive plant and experimental venue affect tadpole performance. Biological Invasions 8, 327–338.

[brv70058-bib-0056] Brundu, G. & Camarda, I. (2013). The Flora of Chad: a checklist and brief analysis. PhytoKeys 23, 1–18. 10.3897/phytokeys.23.4752 PMC3690977 23805051

[brv70058-bib-0057] Buczkowski, G. & Bertelsmeier, C. (2017). Invasive termites in a changing climate: a global perspective. Ecology and Evolution 7, 974–985. 28168033 10.1002/ece3.2674 PMC5288252

[brv70058-bib-0058] Burgess, T. I. , Scott, J. K. , Mcdougall, K. L. , Stukely, M. J. C. , Crane, C. , Dunstan, W. A. , Brigg, F. , Andjic, V. , White, D. , Rudman, T. , Arentz, F. , Ota, N. & Hardy, G. E. S. J. (2017). Current and projected global distribution of *Phytophthora cinnamomi*, one of the world's worst plant pathogens. Global Change Biology 23, 1661–1674. 27596590 10.1111/gcb.13492

[brv70058-bib-0059] CABI (2022). Ceratocystis fimbriata (Ceratocystis blight). CABI Compendium. CAB International, Wallingford. 12143. 10.1079/cabicompendium.12143.

[brv70058-bib-0060] CABI (2009). Claviceps africana (ergot). CABI Compendium. CAB International, Wallingford. 13787. 10.1079/cabicompendium.13787.

[brv70058-bib-0061] CABI (2021). Puccinia graminis (stem rust of cereals). CABI Compendium. CAB International, Wallingford. 45797. 10.1079/cabicompendium.45797.

[brv70058-bib-0062] Cáceres‐Polgrossi, L. , Di Rico, M. , Parra, D. , Seebens, H. , Galvin, S. D. & Boehmer, H. J. (2023). The relationship between naturalized alien and native plant species: insights from oceanic islands of the south‐east Pacific over the last 200 years. NeoBiota 86, 21–43.

[brv70058-bib-0063] Campbell, F. (2010). Erythrina gall wasp: Quadrastichus erythrinae. Don't Move Firewood. https://www.dontmovefirewood.org/pest_pathogen/erythrina-gall-wasp-html/.

[brv70058-bib-0064] Campbell, M. L. , Hewitt, C. L. & Miles, J. (2016). Marine pests in paradise: capacity building, awareness raising and preliminary introduced species port survey results in the Republic of Palau. Management of Biological Invasions 7, 351–363.

[brv70058-bib-0065] Capinha, C. , Seebens, H. , Cassey, P. , García‐Díaz, P. , Lenzner, B. , Mang, T. , Moser, D. , Pyšek, P. , Rödder, D. , Scalera, R. , Winter, M. , Dullinger, S. & Essl, F. (2017). Diversity, biogeography and the global flows of alien amphibians and reptiles. Diversity and Distributions 23, 1313–1322.

[brv70058-bib-0066] Capizzi, D. (2020). A review of mammal eradications on Mediterranean islands. Mammal Review 50, 124–135.

[brv70058-bib-0067] Cárdenas, L. , Leclerc, J.‐C. , Bruning, P. , Garrido, I. , Détrée, C. , Figueroa, A. , Astorga, M. , Navarro, J. M. , Johnson, L. E. , Carlton, J. T. & Pardo, L. (2020). First mussel settlement observed in Antarctica reveals the potential for future invasions. Scientific Reports 10, 5552. 32218472 10.1038/s41598-020-62340-0 PMC7099062

[brv70058-bib-0068] Carlton, J. T. & Eldrege, L. G. (2015). Update and revision of the marine bioinvasions of Hawai'i: the introduced and cryptogenic marine and estuarine animals and plants of the Hawaiian archipelago. In Lucius G. Eldredge III Memorial Volume: Tribute to a Polymath (eds N. L. Evenhuis and J. T. Carlton ), pp. 25–47. Bishop Museum Press, Honolulu, Hawai'i.

[brv70058-bib-0069] Carlton, J. T. & Schwindt, E. (2024). The assessment of marine bioinvasion diversity and history. Biological Invasions 26, 237–298.

[brv70058-bib-0070] Carnegie, A. J. & Giblin, F. R. (2022). Austropuccinia psidii (myrtle rust). CABI Compendium, CAB International, Wallingford. 45846. 10.1079/cabicompendium.45846

[brv70058-bib-0071] Carneiro, V. C. , Galil, B. & Lyko, F. (2023). A voyage into the levant: the first record of a marbled crayfish *Procambarus virginalis* (Lyko, 2017) population in Israel. BioInvasions Records 12, 829–836.

[brv70058-bib-0072] Casal, C. M. V. , Luna, S. , Froese, R. , Bailly, N. , Atanacio, R. & Agbayani, E. (2007). Alien fish species in The Philippines: pathways, biological characteristics, establishment and invasiveness. Journal of Environmental Science and Management 10, 1–9.

[brv70058-bib-0073] Castilla, J. C. & Neill, P. E. (2009). Marine bioinvasions in the southeastern Pacific: status, ecology, economic impacts, conservation and management. In In Biological Invasions in Marine Ecosystems: Ecological, Management, and Geographic Perspectives (eds G. Rilov and J. A. Crooks ), pp. 439–457. Springer, Berlin, Heidelberg.

[brv70058-bib-0074] Castro Monzon, F. , Rödel, M.‐O. , Ruland, F. , Parra‐Olea, G. & Jeschke, J. M. (2022). *Batrachochytrium salamandrivorans*' amphibian host species and invasion range. EcoHealth 19, 475–486. 36611108 10.1007/s10393-022-01620-9 PMC9898388

[brv70058-bib-0075] Celesti‐Grapow, L. , Alessandrini, A. , Arrigoni, P. V. , Banfi, E. , Bernardo, L. , Bovio, M. , Brundu, G. , Cagiotti, M. R. , Camarda, I. , Carli, E. , Conti, F. , Fascetti, S. , Galasso, G. , Gubellini, L. , La Valva, V. , *et al*. (2009). Inventory of the non‐native flora of Italy. Plant Biosystems 143, 386–430.

[brv70058-bib-0076] Chacón, E. & Saborío, G. (2012). Red Interamericana de Información de Especies Invasoras, Costa Rica. Asociación para la Conservación y el Estudio de la Biodiversidad, San José.

[brv70058-bib-0077] Chainho, P. , Fernandes, A. , Amorim, A. , Ávila, S. P. , Canning‐Clode, J. , Castro, J. J. , Costa, A. C. , Costa, J. L. , Cruz, T. , Gollasch, S. , Grazziotin‐Soares, C. , Melo, R. , Micael, J. , Parente, M. I. , Semedo, J. , *et al*. (2015). Non‐indigenous species in Portuguese coastal areas, coastal lagoons, estuaries and islands. Estuarine, Coastal and Shelf Science 167, 199–211.

[brv70058-bib-0078] Chan, F. T. , Stanislawczyk, K. , Sneekes, A. C. , Dvoretsky, A. , Gollasch, S. , Minchin, D. , David, M. , Jelmert, A. , Albretsen, J. & Bailey, S. A. (2019). Climate change opens new frontiers for marine species in the Arctic: current trends and future invasion risks. Global Change Biology 25, 25–38. 30295388 10.1111/gcb.14469 PMC7379606

[brv70058-bib-0079] Chapple, D. G. , Knegtmans, J. , Kikillus, H. & van Winkel, D. (2016). Biosecurity of exotic reptiles and amphibians in New Zealand: building upon Tony Whitaker's legacy. Journal of the Royal Society of New Zealand 46, 66–84.

[brv70058-bib-0080] Cheke, A. S. & Hume, J. P. (2008). Lost Land of the Dodo: An Ecological History of Mauritius, Réunion & Rodrigues. Yale University Press, New Haven.

[brv70058-bib-0081] Chen, Y. (2008). Global potential distribution of an invasive species, the yellow crazy ant (*Anoplolepis gracilipes*) under climate change. Integrative Zoology 3, 166–175. 21396065 10.1111/j.1749-4877.2008.00095.x

[brv70058-bib-0082] Chen, C. , Wang, Q.‐H. , Wu, J.‐Y. , Huang, D. , Zhang, W.‐H. , Zhao, N. , Li, X.‐F. & Wang, L.‐X. (2017 *a*). Historical introduction, geographical distribution, and biological characteristics of alien plants in China. Biodiversity and Conservation 26, 353–381.

[brv70058-bib-0083] Chen, Y. , Sun, C. & Zhan, A. (2017 *b*). Biological invasions in aquatic ecosystems in China. Aquatic Ecosystem Health & Management 20, 402–412.

[brv70058-bib-0084] Chinea, J. D. & Helmer, E. H. (2003). Diversity and composition of tropical secondary forests recovering from large‐scale clearing: results from the 1990 inventory in Puerto Rico. Forest Ecology and Management 180, 227–240.

[brv70058-bib-0085] Chown, S. L. , Huiskes, A. H. L. , Gremmen, N. J. M. , Lee, J. E. , Terauds, A. , Crosbie, K. , Frenot, Y. , Hughes, K. A. , Imura, S. , Kiefer, K. , Lebouvier, M. , Raymond, B. , Tsujimoto, M. , Ware, C. , Van de Vijver, B. , *et al*. (2012). Continent‐wide risk assessment for the establishment of nonindigenous species in Antarctica. Proceedings of the National Academy of Sciences 109, 4938–4943. 10.1073/pnas.1119787109 PMC3323995 22393003

[brv70058-bib-0086] Chwedorzewska, K. J. , Giełwanowska, I. , Olech, M. , Molina‐Montenegro, M. A. , Wódkiewicz, M. & Galera, H. (2015). *Poa annua* L. in the maritime Antarctic: an overview. Polar Record 51, 637–643.

[brv70058-bib-0087] Chwedorzewska, K. J. , Korczak‐Abshire, M. & Znój, A. (2020). Is Antarctica under threat of alien species invasion? Global Change Biology 26, 1942–1943. 31981270 10.1111/gcb.15013

[brv70058-bib-0088] Chytrý, M. , Wild, J. , Pyšek, P. , Jarošík, V. , Dendoncker, N. , Reginster, I. , Pino, J. , Maskell, L. C. , Vilà, M. , Pergl, J. , Kühn, I. , Spangenberg, J. H. & Settele, J. (2012). Projecting trends in plant invasions in Europe under different scenarios of future land‐use change. Global Ecology and Biogeography 21, 75–87.

[brv70058-bib-0089] Çinar, M. E. , Bilecenoğlu, M. , Öztürk, B. , Katagan, T. & Aysel, V. (2005). Alien species on the coasts of Turkey. Mediterranean Marine Science 6, 119–146.

[brv70058-bib-0090] Clavero, M. , Araujo, R. , Calzada, J. , Delibes, M. , Fernández, N. , Gutiérrez‐Expósito, C. , Revilla, E. & Román, J. (2012). The first invasive bivalve in African fresh waters: invasion portrait and management options. Aquatic Conservation: Marine and Freshwater Ecosystems 22, 277–280.

[brv70058-bib-0091] Clavero, M. & García‐Berthou, E. (2006). Homogenization dynamics and introduction routes of invasive freshwater fish in the Iberian Peninsula. Ecological Applications 16, 2313–2324. 17205906 10.1890/1051-0761(2006)016[2313:hdairo]2.0.co;2

[brv70058-bib-0092] Coetzee, M. P. A. , Wingfield, B. D. , Harrington, T. C. , Steimel, J. , Coutinho, T. A. & Wingfield, M. J. (2001). The root rot fungus *Armillaria mellea* introduced into South Africa by early Dutch settlers. Molecular Ecology 10, 387–396. 11298953 10.1046/j.1365-294x.2001.01187.x

[brv70058-bib-0093] Cohen, A. N. & Carlton, J. T. (1998). Accelerating invasion rate in a highly invaded estuary. Science 279, 555–558. 9438847 10.1126/science.279.5350.555

[brv70058-bib-0094] Coles, S. L. , Reath, P. R. , Skelton, P. A. , Bonito, V. , DeFelice, R. C. & Basch, L. (2003). Introduced Marine Species in Pago Pago Harbor, Fagatele Bay and the National Park Coast. American Samoa, Bishop Museum, Honolulu.

[brv70058-bib-0095] Colwell, R. (1996). Global climate and infectious disease: the cholera paradigm. Science 274, 2025–2031. 8953025 10.1126/science.274.5295.2025

[brv70058-bib-0096] Copeland, C. A. , Harper, R. W. , Brazee, N. J. & Bowlick, F. J. (2023). A review of Dutch elm disease and new prospects for *Ulmus americana* in the urban environment. Arboricultural Journal 45, 3–29.

[brv70058-bib-0097] Corlett, R. T. , Leven, M. R. , Yong, D. L. , Eaton, J. A. & Round, P. D. (2020). Continental analysis of invasive birds: Asia. In Invasive Birds: Global Trends and Impacts, First Edition (eds C. T. Downs and L. A. Hart ), pp. 315–340. CAB International, Wallingford.

[brv70058-bib-0098] Correa, A. M. D. , Galdames, C. & De Stapf, M. S. (2004). Catálogo de las plantas vasculares de Panamá. Smithsonian Tropical Research Institute, Bogotá.

[brv70058-bib-0099] Courchamp, F. , Fournier, A. , Bellard, C. , Bertelsmeier, C. , Bonnaud, E. , Jeschke, J. M. & Russell, J. C. (2017). Invasion biology: specific problems and possible solutions. Trends in Ecology & Evolution 32, 13–22. 27889080 10.1016/j.tree.2016.11.001

[brv70058-bib-0100] Courtenay, W. R. & Meffe, G. K. (1989). Small fishes in strange places: a review of introduced poeciliids. In Ecology and Evolution of Livebearing Fishes (Poeciliidae) (eds G. K. Meffe and F. F. Snelson ), pp. 319–331. Prentice‐Hall, Englewood Cliffs.

[brv70058-bib-0101] Cowie, R. H. (2001). Invertebrate invasions on Pacific Islands and the replacement of unique native faunas: a synthesis of the land and freshwater snails. Biological Invasions 3, 119–136.

[brv70058-bib-0102] Craig, J. F. (1992). Human‐induced changes in the composition of fish communities in the African Great Lakes. Reviews in Fish Biology and Fisheries 2, 93–124.

[brv70058-bib-0103] Crall, A. W. , Meyerson, L. A. , Stohlgren, T. J. , Jarnevich, C. S. , Newman, G. J. & Graham, J. (2006). Show me the numbers: what data currently exist for non‐native species in the USA? Frontiers in Ecology and the Environment 4, 414–418.

[brv70058-bib-0104] Crosby, A. W. (1986). Ecological Imperialism: The Biological Expansion of Europe, 900–1900, First Edition. Cambridge University Press, Cambridge, New York.

[brv70058-bib-0105] Darwall, W. R. T. , Smith, K. G. , Allen, D. J. , Holland, R. A. , Harrison, I. J. & Brooks, E. G. E. (2011). The diversity of life in African fresh‐waters: underwater, under threat. In An Analysis of the Status and Distribution of Freshwater Species throughout Mainland Africa. IUCN, Cambridge and Gland.

[brv70058-bib-0106] Davison, E. (2022). Phytophthora Cinnamomi (Phytophthora Dieback). CABI Compendium, Wallingford. 40957. https://www.cabidigitallibrary.org/doi/full/10.1079/cabicompendium.40957

[brv70058-bib-0107] Dawson, W. , Moser, D. , van Kleunen, M. , Kreft, H. , Pergl, J. , Pyšek, P. , Weigelt, P. , Winter, M. , Lenzner, B. , Blackburn, T. M. , Dyer, E. E. , Cassey, P. , Scrivens, S. L. , Economo, E. P. , Guénard, B. , *et al*. (2017). Global hotspots and correlates of alien species richness across taxonomic groups. Nature Ecology & Evolution 1, 186.

[brv70058-bib-0108] Day, R. , Abrahams, P. , Bateman, M. , Beale, T. , Clottey, V. , Cock, M. , Colmenarez, Y. , Corniani, N. , Early, R. , Godwin, J. , Gomez, J. , Moreno, P. G. , Murphy, S. T. , Oppong‐Mensah, B. , Phiri, N. , *et al*. (2017). Fall armyworm: impacts and implications for Africa. Outlooks on Pest Management 28, 196–201.

[brv70058-bib-0109] De Groot, M. , Povz, M. , Jernej, J. , Vrezec, A. , Ogris, N. , Kus Veenvliet, J. , Wong, L. J. & Pagad, S. (2020). Global Register of Introduced and Invasive Species – Slovenia. Invasive Species Specialist Group ISSG. https://www.gbif.org/dataset/628b9441-36ba-41bc-a260-be5c655bd04b.

[brv70058-bib-0110] De Pooter, D. , Appeltans, W. , Bailly, N. , Bristol, S. , Deneudt, K. , Eliezer, M. , Fujioka, E. , Giorgetti, A. , Goldstein, P. , Lewis, M. , Lipizer, M. , Mackay, K. , Marin, M. , Moncoiffé, G. , Nikolopoulou, S. , *et al*. (2017). Toward a new data standard for combined marine biological and environmental datasets – expanding OBIS beyond species occurrences. Biodiversity Data Journal 5, e10989. 10.3897/BDJ.5.e10989 PMC5345125 28325978

[brv70058-bib-0111] Desprez‐Loustau, M.‐L. (2009). Alien fungi of Europe. In Handbook of Alien Species in Europe, pp. 15–28. Springer, Dordrecht.

[brv70058-bib-0112] Desprez‐Loustau, M.‐L. , Courtecuisse, R. , Robin, C. , Husson, C. , Moreau, P.‐A. , Blancard, D. , Selosse, M.‐A. , Lung‐Escarmant, B. , Piou, D. & Sache, I. (2010). Species diversity and drivers of spread of alien fungi (sensu lato) in Europe with a particular focus on France. Biological Invasions 12, 157–172.

[brv70058-bib-0113] Diagne, C. , Leroy, B. , Vaissière, A.‐C. , Gozlan, R. E. , Roiz, D. , Jarić, I. , Salles, J.‐M. , Bradshaw, C. J. A. & Courchamp, F. (2021). High and rising economic costs of biological invasions worldwide. Nature 592, 571–576. 33790468 10.1038/s41586-021-03405-6

[brv70058-bib-0114] Dickie, I. A. , Bolstridge, N. , Cooper, J. A. & Peltzer, D. A. (2010). Co‐invasion by *Pinus* and its mycorrhizal fungi. New Phytologist 187, 475–484. 20456067 10.1111/j.1469-8137.2010.03277.x

[brv70058-bib-0115] Dong, X. , Ju, T. , Grenouillet, G. , Laffaille, P. , Lek, S. & Liu, J. (2020). Spatial pattern and determinants of global invasion risk of an invasive species, sharpbelly *Hemiculter leucisculus* (Basilesky, 1855). Science of the Total Environment 711, 134661. 31812402 10.1016/j.scitotenv.2019.134661

[brv70058-bib-0116] Doria, C. R. d. C. , Agudelo, E. , Akama, A. , Barros, B. , Bonfim, M. , Carneiro, L. , Briglia‐Ferreira, S. R. , Nobre Carvalho, L. , Bonilla‐Castillo, C. A. , Charvet, P. , dos Santos Catâneo, D. T. B. , da Silva, H. P. , Garcia‐Dávila, C. R. , dos Anjos, H. D. B. , Duponchelle, F. , *et al*. (2021). The silent threat of non‐native fish in the Amazon: ANNF database and review. Frontiers in Ecology and Evolution 9, 646702.

[brv70058-bib-0117] dos Santos, L. A. , Mendes, M. F. , Krüger, A. P. , Blauth, M. L. , Gottschalk, M. S. & Garcia, F. R. M. (2017). Global potential distribution of *Drosophila suzukii* (Diptera, Drosophilidae). PLoS One 12, e0174318. 28323903 10.1371/journal.pone.0174318 PMC5360346

[brv70058-bib-0118] Douda, K. , Zieritz, A. , Vodáková, B. , Urbańska, M. , Bolotov, I. N. , Marková, J. , Froufe, E. , Bogan, A. E. & Lopes‐Lima, M. (2025). Review of the globally invasive freshwater mussels in the genus Sinanodonta Modell, 1945. Hydrobiologia 852, 1243–1273.

[brv70058-bib-0119] Dubey, S. C. , Gupta, K. , Akhtar, J. , Chalam, V. C. , Singh, M. C. , Khan, Z. , Singh, S. P. , Kumar, P. , Gawade, B. H. , Kiran, R. , Boopathi, T. & Kumari, P. (2021). Plant quarantine for biosecurity during transboundary movement of plant genetic resources. Indian Phytopathology 74, 495–508.

[brv70058-bib-0120] Duffy, G. A. , Coetzee, B. W. T. , Latombe, G. , Akerman, A. H. , McGeoch, M. A. & Chown, S. L. (2017). Barriers to globally invasive species are weakening across the Antarctic. Diversity and Distributions 23, 982–996.

[brv70058-bib-0121] Dullinger, I. , Wessely, J. , Bossdorf, O. , Dawson, W. , Essl, F. , Gattringer, A. , Klonner, G. , Kreft, H. , Kuttner, M. , Moser, D. , Pergl, J. , Pyšek, P. , Thuiller, W. , van Kleunen, M. , Weigelt, P. , *et al*. (2017). Climate change will increase the naturalization risk from garden plants in Europe. Global Ecology and Biogeography 26, 43–53. 28111525 10.1111/geb.12512 PMC5216452

[brv70058-bib-0122] Dutech, C. , Barrès, B. , Bridier, J. , Robin, C. , Milgroom, M. G. & Ravigné, V. (2012). The chestnut blight fungus world tour: successive introduction events from diverse origins in an invasive plant fungal pathogen. Molecular Ecology 21, 3931–3946. 22548317 10.1111/j.1365-294X.2012.05575.x

[brv70058-bib-0123] Dutton, J. (1994). Introduced mammals in Sao Tome and Principe: possible threats to biodiversity. Biodiversity and Conservation 3, 927–938.

[brv70058-bib-0124] Dyer, E. E. , Cassey, P. , Redding, D. W. , Collen, B. , Franks, V. , Gaston, K. J. , Jones, K. E. , Kark, S. , Orme, C. D. L. & Blackburn, T. M. (2017 *a*). The global distribution and drivers of alien bird species richness. PLoS Biology 15, e2000942. 28081142 10.1371/journal.pbio.2000942 PMC5230740

[brv70058-bib-0125] Dyer, E. E. , Redding, D. W. & Blackburn, T. M. (2017 *b*). The global avian invasions atlas, a database of alien bird distributions worldwide. Scientific Data 4, 170041. 28350387 10.1038/sdata.2017.41 PMC5369319

[brv70058-bib-0126] Early, R. , Bradley, B. A. , Dukes, J. S. , Lawler, J. J. , Olden, J. D. , Blumenthal, D. M. , Gonzalez, P. , Grosholz, E. D. , Ibañez, I. , Miller, L. P. , Sorte, C. J. B. & Tatem, A. J. (2016). Global threats from invasive alien species in the twenty‐first century and national response capacities. Nature Communications 7, 12485. 10.1038/ncomms12485 PMC4996970 27549569

[brv70058-bib-0127] Early, R. , González‐Moreno, P. , Murphy, S. T. & Day, R. (2018). Forecasting the global extent of invasion of the cereal pest *Spodoptera frugiperda*, the fall armyworm. Neobiota 40, 25–50.

[brv70058-bib-0128] Editorial Board of AquaNIS (2024). Aquatic Non‐Indigeneous and Cryptogenic Species Database (AquaNIS) Information system on Aquatic Non‐Indigenous and Cryptogenic Species. World Wide Web electronic publication, Version 2.36+.

[brv70058-bib-0129] Ellender, B. & Weyl, O. (2014). A review of current knowledge, risk and ecological impacts associated with non‐native freshwater fish introductions in South Africa. Aquatic Invasions 9, 117–132.

[brv70058-bib-0130] Engeman, R. M. , Shiels, A. B. & Clark, C. S. (2018). Objectives and integrated approaches for the control of brown tree snakes: an updated overview. Journal of Environmental Management 219, 115–124. 29738931 10.1016/j.jenvman.2018.04.092

[brv70058-bib-0131] Englund, R. A. (2002). The loss of native biodiversity and continuing nonindigenous species introductions in freshwater, estuarine, and wetland communities of Pearl Harbor, Oahu, Hawaiian islands. Estuaries 25, 418–430.

[brv70058-bib-0132] Enríquez, N. , Pertierra, L. R. , Tejedo, P. , Benayas, J. , Greenslade, P. & Luciáñez, M. J. (2019). The importance of long‐term surveys on species introductions in maritime Antarctica: first detection of *Ceratophysella succinea* (collembola: Hypogastruridae). Polar Biology 42, 1047–1051.

[brv70058-bib-0133] EPPO (2016). *Xanthomonas axonopodis* pv. *allii*. EPPO Bulletin 46, 4–7.

[brv70058-bib-0134] EPPO (2024). Melampsora medusae. EPPO data sheets on pests recommended for regulation. https://gd.eppo.int.

[brv70058-bib-0135] Essl, F. , Dawson, W. , Kreft, H. , Pergl, J. , Pyšek, P. , Van Kleunen, M. , Weigelt, P. , Mang, T. , Dullinger, S. , Lenzner, B. , Moser, D. , Maurel, N. , Seebens, H. , Stein, A. , Weber, E. , *et al*. (2019). Drivers of the relative richness of naturalized and invasive plant species on earth. AoB Plants 11, plz051. 31636882 10.1093/aobpla/plz051 PMC6795282

[brv70058-bib-0136] Essl, F. & Lambdon, P. W. (2009). Alien bryophytes and lichens of Europe. In Handbook of Alien Species in Europe (ed. DAISIE ), pp. 29–41. Springer, Dordrecht.

[brv70058-bib-0137] Essl, F. , Steinbauer, K. , Dullinger, S. , Mang, T. & Moser, D. (2014). Little, but increasing evidence of impacts by alien bryophytes. Biological Invasions 16, 1175–1184.

[brv70058-bib-0138] Faulkner, K. T. , Robertson, M. P. & Wilson, J. R. U. (2020). Stronger regional biosecurity is essential to prevent hundreds of harmful biological invasions. Global Change Biology 26, 2449–2462. 31957142 10.1111/gcb.15006

[brv70058-bib-0139] Feldmeier, S. , Schefczyk, L. , Wagner, N. , Heinemann, G. , Veith, M. & Lötters, S. (2016). Exploring the distribution of the spreading lethal salamander chytrid fungus in its invasive range in Europe – a macroecological approach. PLoS One 11, e0165682. 27798698 10.1371/journal.pone.0165682 PMC5087956

[brv70058-bib-0140] Ficetola, G. F. , Coïc, C. , Detaint, M. , Berroneau, M. , Lorvelec, O. & Miaud, C. (2007). Pattern of distribution of the American bullfrog *Rana catesbeiana* in Europe. Biological Invasions 9, 767–772.

[brv70058-bib-0141] Fisher, M. C. , Garner, T. W. J. & Walker, S. F. (2009). Global emergence of *Batrachochytrium dendrobatidis* and amphibian chytridiomycosis in space, time, and host. Annual Review of Microbiology 63, 291–310. 10.1146/annurev.micro.091208.073435 19575560

[brv70058-bib-0142] Fisher, M. C. , Gurr, S. J. , Cuomo, C. A. , Blehert, D. S. , Jin, H. , Stukenbrock, E. H. , Stajich, J. E. , Kahmann, R. , Boone, C. , Denning, D. W. , Gow, N. A. R. , Klein, B. S. , Kronstad, J. W. , Sheppard, D. C. , Taylor, J. W. , *et al*. (2020). Threats posed by the fungal kingdom to humans, wildlife, and agriculture. MBio 11, e00449‐20. 32371596 10.1128/mBio.00449-20 PMC7403777

[brv70058-bib-0143] Fisher, M. C. , Henk, D. A. , Briggs, C. J. , Brownstein, J. S. , Madoff, L. C. , McCraw, S. L. & Gurr, S. J. (2012). Emerging fungal threats to animal, plant and ecosystem health. Nature 484, 186–194. 22498624 10.1038/nature10947 PMC3821985

[brv70058-bib-0144] Fonseca, É. , Both, C. & Cechin, S. Z. (2019). Introduction pathways and socio‐economic variables drive the distribution of alien amphibians and reptiles in a megadiverse country. Diversity and Distributions 25, 1130–1141.

[brv70058-bib-0145] Forey, E. , Lodhar, S. Y. F. , Galvin, S. D. , Lowry, J. H. , Gopaul, S. , Hanson, G. , Carboni, M. , Chauvat, M. & Boehmer, H. J. (2023). Alien palm invasion leads to selective biotic filtering of resident plant communities towards competitive functional traits. Biological Invasions 25, 1489–1508.

[brv70058-bib-0146] Forsyth, D. M. , Ramsey, D. S. L. , Perry, M. , McKay, M. & Wright, E. F. (2018). Control history, longitude and multiple abiotic and biotic variables predict the abundances of invasive brushtail possums in New Zealand forests. Biological Invasions 20, 2209–2225.

[brv70058-bib-0147] Forti, L. R. , Becker, C. G. , Tacioli, L. , Pereira, V. R. , Santos, A. C. F. A. , Oliveira, I. , Haddad, C. F. B. & Toledo, L. F. (2017). Perspectives on invasive amphibians in Brazil. PLoS One 12, e0184703. 28938024 10.1371/journal.pone.0184703 PMC5609743

[brv70058-bib-0148] Fortini, L. B. , Kaiser, L. R. , Keith, L. M. , Price, J. , Hughes, R. F. , Jacobi, J. D. & Friday, J. B. (2019). The evolving threat of Rapid ‘Ōhi‘a Death (ROD) to Hawai‘i's native ecosystems and rare plant species. Forest Ecology and Management 448, 376–385.

[brv70058-bib-0149] Frehse, F. D. A. , Braga, R. R. , Nocera, G. A. & Vitule, J. R. S. (2016). Non‐native species and invasion biology in a megadiverse country: scientometric analysis and ecological interactions in Brazil. Biological Invasions 18, 3713–3725.

[brv70058-bib-0150] Frenot, Y. , Chown, S. L. , Whinam, J. , Selkirk, P. M. , Convey, P. , Skotnicki, M. & Bergstrom, D. M. (2005). Biological invasions in the Antarctic: extent, impacts and implications. Biological Reviews 80, 45–72. 15727038 10.1017/s1464793104006542

[brv70058-bib-0151] Fritsch, C. (1895). Ueber die Auffindung einer marinen Hydrocharidee im Mittelmeer. Verhandlungen Der Zoologisch‐Botanischen Gesellschaft Wien 45, 104–106.

[brv70058-bib-0152] Fuentes, N. , Marticorena, A. , Saldaña, A. , Jerez, V. , Ortiz, J. C. , Victoriano, P. , Moreno, R. A. , Larraín, J. , Villaseñor‐Parada, C. , Palfner, G. , Sánchez, P. & Pauchard, A. (2020). Multi‐taxa inventory of naturalized species in Chile. NeoBiota 60, 25–41.

[brv70058-bib-0153] Fuentes, N. , Ugarte, E. , Kühn, I. & Klotz, S. (2008). Alien plants in Chile: inferring invasion periods from herbarium records. Biological Invasions 10, 649–657.

[brv70058-bib-0154] Fuentes‐Lillo, E. , Cuba‐Díaz, M. , Troncoso‐Castro, J. M. & Rondanelli‐Reyes, M. (2017). Seeds of non‐native species in King George Island soil. Antarctic Science 29, 324–330.

[brv70058-bib-0155] Fuller, D. Q. & Boivin, N. (2009). Crops, cattle and commensals across the Indian Ocean: current and potential archaeobiological evidence. Études Océan Indien, 42‐43, 13–46. 10.4000/oceanindien.698.

[brv70058-bib-0156] Fuller, P. L. , Nico, L. G. & Williams, J. D. (1999). Nonindigenous Fishes Introduced into Inland Waters of the United States. American Fisheries Society, Bethesda.

[brv70058-bib-0157] Gadoury, D. M. , Cadle‐Davidson, L. , Wilcox, W. F. , Dry, I. B. , Seem, R. C. & Milgroom, M. G. (2012). Grapevine powdery mildew (*Erysiphe necator*): a fascinating system for the study of the biology, ecology and epidemiology of an obligate biotroph. Molecular Plant Pathology 13, 1–16. 21726395 10.1111/j.1364-3703.2011.00728.x PMC6638670

[brv70058-bib-0158] Galanidi, M. , Aissi, M. , Ali, M. , Bakalem, A. , Bariche, M. , Bartolo, A. G. , Bazairi, H. , Beqiraj, S. , Bilecenoglu, M. , Bitar, G. , Bugeja, M. , Carbonell, A. , Castriota, L. , Chalabi, A. , Çinar, M. E. , *et al*. (2023). Validated inventories of non‐indigenous species (NIS) for the Mediterranean Sea as tools for regional policy and patterns of NIS spread. Diversity 15, 962.

[brv70058-bib-0159] Galera, H. , Chwedorzewska, K. J. , Korczak‐Abshire, M. & Wódkiewicz, M. (2018). What affects the probability of biological invasions in Antarctica? Using an expanded conceptual framework to anticipate the risk of alien species expansion. Biodiversity and Conservation 27, 1789–1809.

[brv70058-bib-0160] Galil, B. S. (2023). A sea, a canal, a disaster: the Suez Canal and the transformation of the Mediterranean biota. In The Suez Canal: Past Lessons and Future Challenges (eds C. Lutmar and Z. Rubinovitz ), pp. 199–215. Springer, Cham.

[brv70058-bib-0161] Galil, B. S. , Marchini, A. , Occhipinti‐Ambrogi, A. , Minchin, D. , Narščius, A. , Ojaveer, H. & Olenin, S. (2014). International arrivals: widespread bioinvasions in European seas. Ethology, Ecology & Evolution 26, 152–171. 10.1080/03949370.2014.897651 PMC4034525 24899770

[brv70058-bib-0162] Galil, B. S. , Mienis, H. K. , Hoffman, R. & Goren, M. (2021). Non‐indigenous species along the Israeli Mediterranean coast: tally, policy, outlook. Hydrobiologia 848, 2011–2029.

[brv70058-bib-0163] Gama, M. A. , Crespo, D. B. , Dolbeth, M. & Anastácio, P. A. (2016). Predicting global habitat suitability for *Corbicula fluminea* using species distribution models: the importance of different environmental datasets. Ecological Modelling 319, 163–169.

[brv70058-bib-0164] Garbelotto, M. & Frankel, S. J. (20202). Phytophthora Ramorum (Sudden Oak Death (SOD)). CABI Compendium 40991, CAB International, Wallingford.

[brv70058-bib-0165] García‐Díaz, P. , Ross, J. V. , Woolnough, A. P. & Cassey, P. (2017). Managing the risk of wildlife disease introduction: pathway‐level biosecurity for preventing the introduction of alien ranaviruses. Journal of Applied Ecology 54, 234–241.

[brv70058-bib-0166] Geils, B. W. , Hummer, K. E. & Hunt, R. S. (2010). White pines, *Ribes*, and blister rust: a review and synthesis. Forest Pathology 40, 147–185.

[brv70058-bib-0167] Genovesi, P. , Carnevali, L. , Alonzi, A. & Scalera, R. (2012). Alien mammals in Europe: updated numbers and trends, and assessment of the effects on biodiversity. Integrative Zoology 7, 247–253. 22938522 10.1111/j.1749-4877.2012.00309.x

[brv70058-bib-0168] Gerlach, J. , Barker, G. M. , Bick, C. S. , Bouchet, P. , Brodie, G. , Christensen, C. C. , Collins, T. , Coote, T. , Cowie, R. H. , Fiedler, G. C. , Griffiths, O. L. , Florens, F. B. V. , Hayes, K. A. , Kim, J. , Meyer, J.‐Y. , *et al*. (2021). Negative impacts of invasive predators used as biological control agents against the pest snail *Lissachatina fulica*: the snail *Euglandina ‘rosea’* and the flatworm *Platydemus manokwari* . Biological Invasions 23, 997–1031.

[brv70058-bib-0169] Gessler, C. , Pertot, I. & Perazzolli, M. (2011). *Plasmopara viticola*: a review of knowledge on downy mildew of grapevine and effective disease management. Phytopathologia Mediterranea 50, 3–44.

[brv70058-bib-0170] Gherardi, F. , Britton, J. R. , Mavuti, K. M. , Pacini, N. , Grey, J. , Tricarico, E. & Harper, D. M. (2011). A review of allodiversity in Lake Naivasha, Kenya: developing conservation actions to protect east African lakes from the negative impacts of alien species. Biological Conservation 144, 2585–2596.

[brv70058-bib-0171] Gherardi, F. , Gollasch, S. , Minchin, D. , Olenin, S. & Panov, V. (2009). Alien invertebrates and fish in European inland waters. In Handbook of Alien Species in Europe (ed. DAISIE ), pp. 81–92. Springer, Dordrecht.

[brv70058-bib-0172] Gilardi, G. , Demarchi, S. , Garibaldi, A. & Gullino, M. L. (2013). Management of downy mildew of sweet basil (*Ocimum basilicum*) caused by *Peronospora belbahrii* by means of resistance inducers, fungicides, biocontrol agents and natural products. Phytoparasitica 41, 59–72.

[brv70058-bib-0173] Giovas, C. M. , LeFebvre, M. J. & Fitzpatrick, S. M. (2012). New records for prehistoric introduction of neotropical mammals to the West Indies: evidence from Carriacou, Lesser Antilles. Journal of Biogeography 39, 476–487.

[brv70058-bib-0174] Gittenberger, A. , Mirimin, L. , Boyd, J. , O'Beirn, F. , Devine, G. , O'Brien, M. , Rensing, M. , O'Dwyer, K. & Gittenberger, E. (2023a). Marine non‐indigenous species dynamics in time and space within the coastal waters of the Republic of Ireland. Diversity 15, 1019.

[brv70058-bib-0175] Gittenberger, A. , Rensing, M. , Faasse, M. , Van Walraven, L. , Smolders, S. , Keeler Perez, H. & Gittenberger, E. (2023b). Non‐indigenous species dynamics in time and space within the coastal waters of The Netherlands. Diversity 15, 719.

[brv70058-bib-0176] Glon, H. , Daly, M. , Carlton, J. T. , Flenniken, M. M. & Currimjee, Z. (2020). Mediators of invasions in the sea: life history strategies and dispersal vectors facilitating global sea anemone introductions. Biological Invasions 22, 3195–3222. 32837266 10.1007/s10530-020-02321-6 PMC7429141

[brv70058-bib-0177] Goldsmit, J. , McKindsey, C. W. , Schlegel, R. W. , Stewart, D. B. , Archambault, P. & Howland, K. L. (2020). What and where? Predicting invasion hotspots in the Arctic marine realm. Global Change Biology 26, 4752–4771. 32407554 10.1111/gcb.15159 PMC7496761

[brv70058-bib-0178] González Martínez, A. I. , Barrios, Y. , De Jesús, S. , Wong, L. J. & Pagad, S. (2020). Global Register of Introduced and Invasive Species – Mexico. Version 1.5. Invasive Species Specialist Group ISSG. 10.15468/08knmc.

[brv70058-bib-0179] Goren, M. & Ortal, R. (1999). Biogeography, diversity and conservation of the inland water fish communities in Israel. Biological Conservation 89, 1–9.

[brv70058-bib-0180] Görg, M. , Ploch, S. , Kruse, J. , Kummer, V. , Runge, F. , Choi, Y.‐J. & Thines, M. (2017). Revision of *Plasmopara* (Oomycota, Peronosporales) parasitic to *Impatiens* . Mycological Progress 16, 791–799.

[brv70058-bib-0181] Gormley, A. M. , Holland, E. P. , Pech, R. P. , Thomson, C. & Reddiex, B. (2012). Impacts of an invasive herbivore on indigenous forests. Journal of Applied Ecology 49, 1296–1305.

[brv70058-bib-0182] Government of India (2005). Ministry of Environment and Forests, Country report on status of forest invasive alien species in India, p. 74. New Delhi: Asia‐Pacific Forest Invasive Species Network.

[brv70058-bib-0183] Gracia, A. , Medellin Mora, J. , Gil Agudelo, D. & Puentes, G. (2011). Guía de las especies introducidas marinas y costeras de Colombia. Bogota: Instituto de Investigaciones Marinas y Costeras (INVEMAR) y Ministerio de Ambiente y Desarrollo Sostenible.

[brv70058-bib-0184] Groom, Q. J. , Adriaens, T. , Desmet, P. , Simpson, A. , De Wever, A. , Bazos, I. , Cardoso, A. C. , Charles, L. , Christopoulou, A. , Gazda, A. , Helmisaari, H. , Hobern, D. , Josefsson, M. , Lucy, F. , Marisavljevic, D. , *et al*. (2017). Seven recommendations to make your invasive alien species data more useful. Frontiers in Applied Mathematics and Statistics 3, 13.

[brv70058-bib-0185] Groom, Q. J. , Desmet, P. , Reyserhove, L. , Adriaens, T. , Oldoni, D. , Vanderhoeven, S. , Baskauf, S. J. , Chapman, A. , McGeoch, M. , Walls, R. , Wieczorek, J. , Wilson, J. R. U. , Zermoglio, P. F. & Simpson, A. (2019). Improving Darwin Core for research and management of alien species. Biodiversity Information Science and Standards 3, e38084.

[brv70058-bib-0186] Gross, A. , Petitcollin, C. , Dutech, C. , Ly, B. , Massot, M. , Faivre d'Arcier, J. , Dubois, L. , Saint‐Jean, G. & Desprez‐Loustau, M.‐L. (2021). Hidden invasion and niche contraction revealed by herbaria specimens in the fungal complex causing oak powdery mildew in Europe. Biological Invasions 23, 885–901.

[brv70058-bib-0187] Gulzar, R. , Wani, S. A. , Hassan, T. , Reddy, C. S. , Shrestha, B. B. , Mukul, S. A. , Shabbir, A. , Iqbal, I. M. , Ranwala, S. M. W. , Dorjee, S. , P , Rashid, I. & Khuroo, A. A. (2024). Looking beyond the political boundaries: an integrated inventory of invasive alien flora of South Asia. Biological Invasions 26, 57–78.

[brv70058-bib-0188] Hack, W. H. (1949). Nota sobre un colémbolo de la Antartida Argentina Achorutes viaticus Tullberg. Notas del Museo de La Plata 14, 211–212.

[brv70058-bib-0189] Haider, S. , Lembrechts, J. , McDougall, K. , Pauchard, A. , Alexander, J. M. , Barros, A. , Cavieres, L. , Rashid, I. , Rew, L. , Aleksanyan, A. , Sierra, J. A. , Aschero, V. , Chisholm, C. , Clark, V. R. , Clavel, J. , *et al*. (2022). Think globally, measure locally: the MIREN standardized protocol for monitoring species distributions along elevation gradients. Ecology and Evolution 12, e8590. 35222963 10.1002/ece3.8590 PMC8844121

[brv70058-bib-0190] Hao, Q. & Ma, J.‐S. (2023). Invasive alien plants in China: an update. Plant Diversity 45, 117–121. 36876311 10.1016/j.pld.2022.11.004 PMC9975470

[brv70058-bib-0191] Hardham, A. R. & Blackman, L. M. (2018). Phytophthora cinnamomi. Molecular Plant Pathology 19, 260–285. 28519717 10.1111/mpp.12568 PMC6637996

[brv70058-bib-0192] Hays, W. S. T. & Conant, S. (2007). Biology and impacts of Pacific Island invasive species. 1. A worldwide review of effects of the small Indian mongoose, *Herpestes javanicus* (carnivora: Herpestidae). Pacific Science 61, 3–16.

[brv70058-bib-0193] Heger, T. & Boehmer, H. J. (2005). The invasion of Central Europe by *Senecio Inaequidens* DC. – a complex biogeographical problem. Erdkunde 59, 34–49.

[brv70058-bib-0194] Heinsohn, T. (2003). Animal translocation: long‐term human influences on the vertebrate zoogeography of Australasia (natural dispersal versus ethnophoresy). Australian Zoologist 32, 351–376.

[brv70058-bib-0195] Helmer, E. H. , Ruzycki, T. S. , Benner, J. , Voggesser, S. M. , Scobie, B. P. , Park, C. , Fanning, D. W. & Ramnarine, S. (2012). Detailed maps of tropical forest types are within reach: forest tree communities for Trinidad and Tobago mapped with multiseason Landsat and multiseason fine‐resolution imagery. Forest Ecology and Management 279, 147–166.

[brv70058-bib-0196] Heluta, V. , Takamatsu, S. , Voytyuk, S. & Shiroya, Y. (2009). *Erysiphe kenjiana* (Erysiphales), a new invasive fungus in Europe. Mycological Progress 8, 367–375.

[brv70058-bib-0197] Henderson, L. (2006). Comparisons of invasive plants in southern Africa originating from southern temperate, northern temperate and tropical regions. Bothalia 36, 201–222.

[brv70058-bib-0198] Henriksen, M. V. , Arlé, E. , Pili, A. , Clarke, D. A. , García‐Berthou, E. , Groom, Q. , Lenzner, B. , Meyer, C. , Seebens, H. , Tingley, R. , Winter, M. & McGeoch, M. A. (2024). Global indicators of the environmental impacts of invasive alien species and their information adequacy. Philosophical Transactions of the Royal Society B: Biological Sciences 379, 20230323. 10.1098/rstb.2023.0323 PMC10999262 38583467

[brv70058-bib-0199] Hernández, G. , Lahmann, E. J. & Pérez‐Gil Salcido, R. (2002). Invasores en Mesoamérica y el Caribe: Resultados del Taller Sobre Especies Invasoras: Ante los Retos de su Presencia en Mesoamérica y el Caribe. UICN, Unión Mundial para la Naturaleza, San José.

[brv70058-bib-0200] Hewitt, C. L. , Campbell, M. L. , Thresher, R. E. , Martin, R. B. , Boyd, S. , Cohen, B. F. , Currie, D. R. , Gomon, M. F. , Keough, M. J. , Lewis, J. A. , Lockett, M. M. , Mays, N. , McArthur, M. A. , O'Hara, T. D. , Poore, G. C. B. , *et al*. (2004). Introduced and cryptogenic species in port Phillip Bay, Victoria, Australia. Marine Biology 144, 183–202.

[brv70058-bib-0201] Hill, M. P. , Bertelsmeier, C. , Clusella‐Trullas, S. , Garnas, J. , Robertson, M. P. & Terblanche, J. S. (2016). Predicted decrease in global climate suitability masks regional complexity of invasive fruit fly species response to climate change. Biological Invasions 18, 1105–1119.

[brv70058-bib-0202] Hill, M. P. , Coetzee, J. A. , Martin, G. D. , Smith, R. & Strange, E. F. (2020). Invasive alien aquatic plants in South African freshwater ecosystems. In Biological Invasions in South Africa (eds B. W. van Wilgen , J. Measey , D. M. Richardson , J. R. Wilson and T. A. Zengeya ), pp. 97–114. Springer, Cham.

[brv70058-bib-0203] Hill, M. P. , Gallardo, B. & Terblanche, J. S. (2017). A global assessment of climatic niche shifts and human influence in insect invasions. Global Ecology and Biogeography 26, 679–689.

[brv70058-bib-0204] Holmes, P. M. , Richardson, D. M. , Esler, K. J. , Witkowski, E. T. F. & Fourie, S. (2005). A decision‐making framework for restoring riparian zones degraded by invasive alien plants in South Africa. South African Journal of Science 101, 553–564.

[brv70058-bib-0205] Howard, G. W. & Chege, F. W. (2007). Invasions by plants in the inland waters and wetlands of Africa. In Biological Invaders in Inland Waters: Profiles, Distribution, and Threats (ed. F. Gherardi ), pp. 193–208. Springer, Dordrecht.

[brv70058-bib-0206] Huang, D. , Haack, R. A. & Zhang, R. (2011). Does global warming increase establishment rates of invasive alien species? A centurial time series analysis. PLoS One 6, e24733. 21931837 10.1371/journal.pone.0024733 PMC3169637

[brv70058-bib-0207] Hughes, K. A. , Convey, P. , Maslen, N. R. & Smith, R. I. L. (2010). Accidental transfer of non‐native soil organisms into Antarctica on construction vehicles. Biological Invasions 12, 875–891.

[brv70058-bib-0208] Hughes, K. A. , Cowan, D. A. & Wilmotte, A. (2015). Protection of Antarctic microbial communities – ‘out of sight, out of mind’. Frontiers in Microbiology 6, 151. 25762992 10.3389/fmicb.2015.00151 PMC4340226

[brv70058-bib-0209] Hughes, K. A. , Lee, J. E. , Tsujimoto, M. , Imura, S. , Bergstrom, D. M. , Ware, C. , Lebouvier, M. , Huiskes, A. H. L. , Gremmen, N. J. M. , Frenot, Y. , Bridge, P. D. & Chown, S. L. (2011). Food for thought: risks of non‐native species transfer to the Antarctic region with fresh produce. Biological Conservation 144, 1682–1689.

[brv70058-bib-0210] Hughes, A. C. , Orr, M. C. , Ma, K. , Costello, M. J. , Waller, J. , Provoost, P. , Yang, Q. , Zhu, C. & Qiao, H. (2021). Sampling biases shape our view of the natural world. Ecography 44, 1259–1269.

[brv70058-bib-0211] Hughes, K. A. & Pertierra, L. R. (2016). Evaluation of non‐native species policy development and implementation within the Antarctic treaty area. Biological Conservation 200, 149–159.

[brv70058-bib-0212] Hughes, K. A. , Pescott, O. L. , Peyton, J. M. , Adriaens, T. , Cottier‐Cook, E. J. , Key, G. , Rabitsch, W. , Tricarico, E. , Barnes, D. K. A. , Baxter, N. , Belchier, M. , Blake, D. , Convey, P. , Dawson, W. , Frohlich, D. , *et al*. (2020). Invasive non‐native species likely to threaten biodiversity and ecosystems in the Antarctic peninsula region. Global Change Biology 26, 2702–2716. 31930639 10.1111/gcb.14938 PMC7154743

[brv70058-bib-0213] Huiskes, A. H. L. , Gremmen, N. J. M. , Bergstrom, D. M. , Frenot, Y. , Hughes, K. A. , Imura, S. , Kiefer, K. , Lebouvier, M. , Lee, J. E. , Tsujimoto, M. , Ware, C. , Van de Vijver, B. & Chown, S. L. (2014). Aliens in Antarctica: assessing transfer of plant propagules by human visitors to reduce invasion risk. Biological Conservation 171, 278–284.

[brv70058-bib-0214] Hussner, A. (2012). Alien aquatic plant species in European countries. Weed Research 52, 297–306.

[brv70058-bib-0215] Hussner, A. , Van de Weyer, K. , Gross, E. M. & Hilt, S. (2010). Comments on increasing number and abundance of non‐indigenous aquatic macrophyte species in Germany. Weed Research 50, 519–526.

[brv70058-bib-0216] ICES (2022). Working Group on Introductions and Transfers of Marine Organisms (WGITMO). ICES Scientific Reports 4, 1–23.

[brv70058-bib-0217] Ihlow, F. , Courant, J. , Secondi, J. , Herrel, A. , Rebelo, R. , Measey, G. J. , Lillo, F. , De Villiers, F. A. , Vogt, S. , De Busschere, C. , Backeljau, T. & Rödder, D. (2016). Impacts of climate change on the global invasion potential of the African clawed frog *Xenopus laevis* . PLoS One 11, e0154869. 27248830 10.1371/journal.pone.0154869 PMC4889038

[brv70058-bib-0218] Imada, C. T. (2019). Hawaiian Native and Naturalized Vascular Plants Checklist. *Bishop Museum Technical Report 69*. Bishop Museum, Honolulu.

[brv70058-bib-0219] Inderjit , Pergl, J. , van Kleunen, M. , Hejda, M. , Babu, C. R. , Majumdar, S. , Singh, P. , Singh, S. P. , Salamma, S. , Rao, B. R. P. & Pyšek, P. (2018). Naturalized alien flora of the Indian states: biogeographic patterns, taxonomic structure and drivers of species richness. Biological Invasions 20, 1625–1638.

[brv70058-bib-0220] IPBES (2019). Summary for Policymakers of the Global Assessment Report on Biodiversity and Ecosystem Services of the Intergovernmental Science‐Policy Platform on Biodiversity and Ecosystem Services. IPBES Secretariat, Bonn.

[brv70058-bib-0221] IPBES (2021). *IPBES regions and sub‐regions* (1.2). IPBES technical support unit on knowledge and data. 10.5281/zenodo.5719431.

[brv70058-bib-0222] IPBES (2023). Thematic Assessment Report on Invasive Alien Species and their Control of the Intergovernmental Science‐Policy Platform on Biodiversity and Ecosystem Services. IPBES Secretariat, Bonn.

[brv70058-bib-0223] IUCN (2000). IUCN Guidelines for the Prevention of Biodiversity Loss Caused by Alien Invasive Species. IUCN Species Survival Commission (SSC), Invasive Species Specialist Group, Gland.

[brv70058-bib-0224] Iwasaki, K. (2006). Human‐mediated introduction of marine organisms in Japan: a review. In Assessment and Control of Biological Invasion Risks (eds F. Koike , M. N. Clout , M. Kawamichi , M. de Poorter and K. Iwatsuki ), pp. 104–112. Shoukadoh Book Sellers, Kyoto Japan and the World Conservation Union (IUCN), Gland.

[brv70058-bib-0225] Jäger, H. , San‐José, M. , Peabody, C. , Chango, R. & Sevilla, C. (2024). Restoring the threatened Scalesia forest: insights from a decade of invasive plant management in Galapagos. Frontiers in Forests and Global Change 7, 1350498.

[brv70058-bib-0226] Janion‐Scheepers, C. & Griffiths, C. L. (2020). Alien terrestrial invertebrates in South Africa. In Biological Invasions in South Africa (eds B. W. van Wilgen , J. Measey , D. M. Richardson , J. R. Wilson and T. A. Zengeya ), pp. 185–205. Springer, Cham.

[brv70058-bib-0227] Jaryan, V. , Uniyal, S. K. , Gupta, R. C. & Singh, R. D. (2013). Alien flora of Indian Himalayan state of Himachal Pradesh. Environmental Monitoring and Assessment 185, 6129–6153. 23196408 10.1007/s10661-012-3013-2

[brv70058-bib-0228] Jensen, K. R. , Andersen, P. , Andersen, N. R. , Bruhn, A. , Buur, H. , Carl, H. , Jakobsen, H. , Jaspers, C. , Lundgreen, K. , Nielsen, R. , Strandberg, B. & Stæhr, P. A. U. (2023). Reviewing introduction histories, pathways, invasiveness, and impact of non‐indigenous species in Danish marine waters. Diversity 15, 434.

[brv70058-bib-0229] Jeschke, J. M. , Aparicio, L. G. , Haider, S. , Heger, T. , Lortie, C. J. , Pyšek, P. & Strayer, D. L. (2012). Taxonomic bias and lack of cross‐taxonomic studies in invasion biology. Frontiers in Ecology and the Environment 10, 349–350.

[brv70058-bib-0230] Jones, D. R. & Baker, R. H. A. (2007). Introductions of non‐native plant pathogens into Great Britain, 1970–2004. Plant Pathology 56, 891–910.

[brv70058-bib-0231] Jones, E. J. , Kraaij, T. , Fritz, H. & Moodley, D. (2019). A global assessment of terrestrial alien ferns (Polypodiophyta): species' traits as drivers of naturalisation and invasion. Biological Invasions 21, 861–873.

[brv70058-bib-0232] Jung, T. , Pérez‐Sierra, A. , Durán, A. , Jung, M. H. , Balci, Y. & Scanu, B. (2018). Canker and decline diseases caused by soil‐ and airborne *Phytophthora* species in forests and woodlands. Persoonia ‐ Molecular Phylogeny and Evolution of Fungi 40, 182–220. 10.3767/persoonia.2018.40.08 PMC6146643 30505001

[brv70058-bib-0233] Kairo, M. , Ali, B. , Cheesman, O. , Haysom, K. & Murphy, S. (2003). Invasive Species Threats in the Caribbean Region. Report to the Nature Conservancy. CAB International, Wallingford, https://caribbeaninvasives.org/wp-content/uploads/2020/12/caribbean_invasives_paper.pdf.

[brv70058-bib-0234] Kark, S. , Solarz, W. , Chiron, F. , Clergeau, P. & Shirley, S. M. (2009). Alien birds, amphibians and reptiles of Europe. In Handbook of Alien Species in Europe (ed. DAISIE ), pp. 105–118. Springer, Dordrecht.

[brv70058-bib-0235] Kartesz, J. T. (2015). The Biota of North America Program (BONAP). Taxonomic Data Center. http://www.bonap.net/tdc.

[brv70058-bib-0236] Keith, P. (2002). Freshwater fish and decapod crustacean populations on Réunion Island, with an assessment of species introductions. Bulletin Français de la Pêche et de la Pisciculture 364, 97–107.

[brv70058-bib-0237] Keppel, G. , Morrison, C. , Meyer, J.‐Y. & Boehmer, H. J. (2014). Isolated and vulnerable: the history and future of Pacific Island terrestrial biodiversity. Pacific Conservation Biology 20, 136.

[brv70058-bib-0238] Kestrup, Å. M. , Smith, D. L. & Therriault, T. W. (eds) (2015). Report of Working Group 21 on Non‐indigenous Aquatic Species. PICES Scientific Report No. 48. North Pacific Marine Science Organization (PICES), Sidney.

[brv70058-bib-0239] Kirkpatrick, W. , Page, A. & Massam, M. (2008). European Rabbit (Oryctolagus cuniculus) risk assessment for Australia. Department of Agriculture and Food, West Australia.

[brv70058-bib-0240] Kiss, L. , Vaghefi, N. , Bransgrove, K. , Dearnaley, J. D. W. , Takamatsu, S. , Tan, Y. P. , Marston, C. , Liu, S.‐Y. , Jin, D.‐N. , Adorada, D. L. , Bailey, J. , Cabrera De Álvarez, M. G. , Daly, A. , Dirchwolf, P. M. , Jones, L. , *et al*. (2020). Australia: a continent without native powdery mildews? The first comprehensive catalog indicates recent introductions and multiple host range expansion events, and leads to the re‐discovery of Salmonomyces as a new lineage of the Erysiphales. Frontiers in Microbiology 11, 1571. 32765452 10.3389/fmicb.2020.01571 PMC7378747

[brv70058-bib-0241] Knapp, C. R. , Iverson, J. B. , Buckner, S. D. & Cant, S. V. (2011). Conservation of Amphibians and Reptiles in The Bahamas. Brill, Leiden.

[brv70058-bib-0242] Koopman, T. , Linde, C. C. , Fourie, P. H. & Mcleod, A. (2007). Population genetic structure of *Plasmopara viticola* in the Western Cape Province of South Africa. Molecular Plant Pathology 8, 723–736. 20507533 10.1111/j.1364-3703.2007.00429.x

[brv70058-bib-0243] Kornis, M. S. , Mercado‐Silva, N. & Vander Zanden, M. J. (2012). Twenty years of invasion: a review of round goby *Neogobius melanostomus* biology, spread and ecological implications. Journal of Fish Biology 80, 235–285. 22268429 10.1111/j.1095-8649.2011.03157.x

[brv70058-bib-0244] Kouba, A. , Petrusek, A. & Kozák, P. (2014). Continental‐wide distribution of crayfish species in Europe: update and maps. Knowledge and Management of Aquatic Ecosystems 413, 5.

[brv70058-bib-0245] Kraus, F. (2009). Alien Reptiles and Amphibians – A Scientific Compendium and Analysis. Springer, Dordrecht.

[brv70058-bib-0246] Kreisel, H. & Scholler, M. (1994). Chronology of phytoparasitic fungi introduced to Germany and adjacent countries. Botanica Acta 107, 387–392.

[brv70058-bib-0247] Kriticos, D. J. , Kean, J. M. , Phillips, C. B. , Senay, S. D. , Acosta, H. & Haye, T. (2017). The potential global distribution of the brown marmorated stink bug, *Halyomorpha halys*, a critical threat to plant biosecurity. Journal of Pest Science 90, 1033–1043.

[brv70058-bib-0248] Kriticos, D. J. , Morin, L. , Leriche, A. , Anderson, R. C. & Caley, P. (2013). Combining a climatic niche model of an invasive fungus with its host species distributions to identify risks to natural assets: *Puccinia psidii* sensu lato in Australia. PLoS One 8, e64479. 23704988 10.1371/journal.pone.0064479 PMC3660372

[brv70058-bib-0249] Kroschel, J. , Sporleder, M. , Tonnang, H. E. Z. , Juarez, H. , Carhuapoma, P. , Gonzales, J. C. & Simon, R. (2013). Predicting climate‐change‐caused changes in global temperature on potato tuber moth *Phthorimaea operculella* (Zeller) distribution and abundance using phenology modeling and GIS mapping. Agricultural and Forest Meteorology 170, 228–241.

[brv70058-bib-0250] Krysko, K. L. , Burgess, J. P. , Rochford, M. R. , Gillette, C. R. , Cueva, D. , Enge, K. M. , Somma, L. A. , Stabile, J. L. , Smith, D. C. , Wasilewski, J. A. , Kieckhefer, G. N. , Granatosky, M. C. & Nielsen, S. V. (2011). Verified non‐indigenous amphibians and reptiles in Florida from 1863 through 2010: outlining the invasion process and identifying invasion pathways and stages. Zootaxa 3028, 1–64.

[brv70058-bib-0251] Krysko, K. L. , Somma, L. A. , Smith, D. C. , Gillette, C. R. , Cueva, D. , Wasilewski, J. A. , Enge, K. M. , Johnson, S. A. , Campbell, T. S. & Edwards, J. R. (2016). New verified nonindigenous amphibians and reptiles in Florida, 1976 through 2015, with a summary of over 152 years of introductions. IRCF Reptiles & Amphibians 23, 110–143.

[brv70058-bib-0252] Kuebbing, S. E. , McCary, M. A. , Lieurance, D. , Nuñez, M. A. , Chiuffo, M. C. , Zhang, B. , Seebens, H. , Simberloff, D. & Meyerson, L. A. (2022). A self‐study of editorial board diversity at Biological invasions. Biological Invasions 24, 321–332.

[brv70058-bib-0253] Lambdon, P. W. , Pyšek, P. , Basnou, C. , Hejda, M. , Arianoutsou, M. , Essl, F. , Jarošík, V. , Pergl, J. , Winter, M. , Anastasiu, P. , Andriopoulos, P. , Bazos, I. , Brundu, G. , Celesti‐Grapow, L. , Chassot, P. , *et al*. (2008). Alien flora of Europe: species diversity, temporal trends, geographical patterns and research needs. Preslia 80, 101–149.

[brv70058-bib-0254] Latombe, G. , Pyšek, P. , Jeschke, J. M. , Blackburn, T. M. , Bacher, S. , Capinha, C. , Costello, M. J. , Fernández, M. , Gregory, R. D. , Hobern, D. , Hui, C. , Jetz, W. , Kumschick, S. , McGrannachan, C. , Pergl, J. , *et al*. (2017). A vision for global monitoring of biological invasions. Biological Conservation 213, 295–308.

[brv70058-bib-0255] Lavoie, C. , Saint‐Louis, A. , Guay, G. & Groeneveld, E. (2012). Les plantes vasculaires exotiques naturalisées: une nouvelle liste pour le Québec. Le Naturaliste Canadien 136, 6–32.

[brv70058-bib-0256] Lazkov, G. A. & Sultanova, B. A. (2011). Checklist of Vascular Plants of Kyrgyzstan (in Russian). Finnish Museum of Natural History, Helsinki.

[brv70058-bib-0257] Lazzaro, L. , Essl, F. , Lugliè, A. , Padedda, B. M. , Pyšek, P. & Brundu, G. (2018). Invasive alien plant impacts on human health and well‐being. In Invasive Dpecies and Human Health (eds G. Mazza and E. Tricarico ), pp. 16–33. CAB International, Wallingford.

[brv70058-bib-0258] Lecomte, F. , Beall, E. , Chat, J. , Davaine, P. & Gaudin, P. (2013). The complete history of salmonid introductions in the Kerguelen Islands, Southern Ocean. Polar Biology 36, 457–475.

[brv70058-bib-0259] Lee, H. & Reusser, D. A. (2012). *Atlas of Nonindigenous Marine and Estuarine Species in the North Pacific*. Office of Research and Development, National Health and Environmental Effects Research Laboratory, EPA/600/R/12/631.

[brv70058-bib-0260] Lee, K.‐H. , Chen, T.‐H. , Shang, G. , Clulow, S. , Yang, Y.‐J. & Lin, S.‐M. (2019). A check list and population trends of invasive amphibians and reptiles in Taiwan. ZooKeys 829, 85–130. 30914838 10.3897/zookeys.829.27535 PMC6422934

[brv70058-bib-0261] Lenz, M.‐I. , Galvin, S. , Keppel, G. , Gopaul, S. , Kowasch, M. , Dyer, M. J. , Watling, D. , Lodhar, S. Y. F. , Hanson, G. C. , Erasmi, S. & Juergen Boehmer, H. (2022). Where to invade next: inaction on biological invasions threatens sustainability in a small Island developing state of the tropical South Pacific. In Sustainable Development: Asia‐Pacific Perspectives (ed. P. S. Low ), pp. 393–406. Cambridge University Press, Cambridge.

[brv70058-bib-0262] Leprieur, F. , Beauchard, O. , Blanchet, S. , Oberdorff, T. & Brosse, S. (2008). Fish invasions in the world's river systems: when natural processes are blurred by human activities. PLoS Biology 6, e28. 18254661 10.1371/journal.pbio.0060028 PMC2225436

[brv70058-bib-0263] Leroy, B. , Bellard, C. , Dias, M. S. , Hugueny, B. , Jézéquel, C. , Leprieur, F. , Oberdorff, T. , Robuchon, M. & Tedesco, P. A. (2023). Major shifts in biogeographic regions of freshwater fishes as evidence of the Anthropocene epoch. Science Advances 9, eadi5502. 37976358 10.1126/sciadv.adi5502 PMC10656075

[brv70058-bib-0264] Lever, C. (1992). They Dined on Eland: The Story of the Acclimatisation Societies. Quiller Press, London.

[brv70058-bib-0265] Lever, C. (2003). Naturalized Reptiles and Amphibians of the World. Oxford University Press, Oxford.

[brv70058-bib-0266] Lewis, J. S. , Farnsworth, M. L. , Burdett, C. L. , Theobald, D. M. , Gray, M. & Miller, R. S. (2017). Biotic and abiotic factors predicting the global distribution and population density of an invasive large mammal. Scientific Reports 7, 44152. 28276519 10.1038/srep44152 PMC5343451

[brv70058-bib-0267] Liebhold, A. M. , Brockerhoff, E. G. , Garrett, L. J. , Parke, J. L. & Britton, K. O. (2012). Live plant imports: the major pathway for forest insect and pathogen invasions of the US. Frontiers in Ecology and the Environment 10, 135–143.

[brv70058-bib-0268] Liebhold, A. M. & Griffin, R. L. (2016). The legacy of Charles Marlatt and efforts to limit plant pest invasions. American Entomologist 62, 218–227.

[brv70058-bib-0269] Liebhold, A. M. , McCullough, D. G. , Blackburn, L. M. , Frankel, S. J. , Von Holle, B. & Aukema, J. E. (2013). A highly aggregated geographical distribution of forest pest invasions in the USA. Diversity and Distributions 19, 1208–1216.

[brv70058-bib-0270] Liebhold, A. M. , Yamanaka, T. , Roques, A. , Augustin, S. , Chown, S. L. , Brockerhoff, E. G. & Pyšek, P. (2016). Global compositional variation among native and non‐native regional insect assemblages emphasizes the importance of pathways. Biological Invasions 18, 893–905.

[brv70058-bib-0271] Linders, T. E. W. , Schaffner, U. , Eschen, R. , Abebe, A. , Choge, S. K. , Nigatu, L. , Mbaabu, P. R. , Shiferaw, H. & Allan, E. (2019). Direct and indirect effects of invasive species: biodiversity loss is a major mechanism by which an invasive tree affects ecosystem functioning. Journal of Ecology 107, 2660–2672.

[brv70058-bib-0272] Lindroth, C. H. (1954). Carabidae common to Europe and North America. The Coleopterists Bulletin 8, 35–52.

[brv70058-bib-0273] Lins, D. M. , de Marco, P. , Andrade, A. F. A. & Rocha, R. M. (2018). Predicting global ascidian invasions. Diversity and Distributions 24, 692–704.

[brv70058-bib-0274] Liu, C. , Comte, L. , Xian, W. , Chen, Y. & Olden, J. D. (2019). Current and projected future risks of freshwater fish invasions in China. Ecography 42, 2074–2083.

[brv70058-bib-0275] Lockwood, J. L. , Welbourne, D. J. , Romagosa, C. M. , Cassey, P. , Mandrak, N. E. , Strecker, A. , Leung, B. , Stringham, O. C. , Udell, B. , Episcopio‐Sturgeon, D. J. , Tlusty, M. F. , Sinclair, J. , Springborn, M. R. , Pienaar, E. F. , Rhyne, A. L. , *et al*. (2019). When pets become pests: the role of the exotic pet trade in producing invasive vertebrate animals. Frontiers in Ecology and the Environment 17, 323–330.

[brv70058-bib-0276] Lomolino, M. V. (2004). Conservation biogeography. In Frontiers of Biogeography: New Directions in the Geography of Nature (eds M. V. Lomolino and L. R. Heaney ), pp. 293–296. Sinauer Associates, Sunderland.

[brv70058-bib-0277] Long, J. L. (2003). Introduced Mammals of the World‐their History, Distribution and Influence. CSIRO Publishing, Collingwood and CABI Publishing, Wallingford.

[brv70058-bib-0278] López, D. N. , Fuentes‐Contreras, E. , Ruiz, C. , Ide, S. & Estay, S. A. (2023). A bug's tale: revealing the history, biogeography and ecological patterns of 500 years of insect invasions. NeoBiota 81, 183–197.

[brv70058-bib-0279] Louis, V. R. , Russek‐Cohen, E. , Choopun, N. , Rivera, I. N. G. , Gangle, B. , Jiang, S. C. , Rubin, A. , Patz, J. A. , Huq, A. & Colwell, R. (2003). Predictability of *Vibrio cholerae* in Chesapeake Bay. Applied and Environmental Microbiology 69, 2773–2785. 12732548 10.1128/AEM.69.5.2773-2785.2003 PMC154498

[brv70058-bib-0280] Louppe, V. , Leroy, B. , Herrel, A. & Veron, G. (2019). Current and future climatic regions favourable for a globally introduced wild carnivore, the raccoon *Procyon lotor* . Scientific Reports 9, 9174. 31235806 10.1038/s41598-019-45713-y PMC6591328

[brv70058-bib-0281] Louppe, V. , Leroy, B. , Herrel, A. & Veron, G. (2020). The globally invasive small Indian mongoose *Urva auropunctata* is likely to spread with climate change. Scientific Reports 10, 7461. 32366920 10.1038/s41598-020-64502-6 PMC7198557

[brv70058-bib-0282] Lowry, B. J. , Lowry, J. H. , Jarvis, K. J. , Keppel, G. , Thaman, R. R. & Boehmer, H. J. (2020). Spatial patterns of presence, abundance, and richness of invasive woody plants in relation to urbanization in a tropical Island setting. Urban Forestry & Urban Greening 48, 126516.

[brv70058-bib-0283] Lu, Y. , Zhao, Q. , Cheng, L. , Zhao, L. , Zhang, H. & Wei, J. (2020). The potential global distribution of the white peach scale *Pseudaulacaspis pentagona* (Targioni Tozzetti) under climate change. Forests 11, 192.

[brv70058-bib-0284] Luo, D. , Wei, H. , Chaichana, R. , Yang, D. , Gu, D. , Mu, X. , Xu, M. , Yang, Y. , Jin, S. & Hu, Y. (2019). Current status and potential risks of established alien fish species in China. Aquatic Ecosystem Health & Management 22, 371–384.

[brv70058-bib-0285] Lutaenko, K. A. , Furota, T. , Nakayama, S. , Shin, K. & Xu, J. (2013). Atlas of Marine Invasive Species in the NOWPAP Region. NOWPAP DINRAC (Northwest Pacific Action Plan, Data and Information Network Regional Center), Beijing.

[brv70058-bib-0286] Mabrouki, Y. & Taybi, A. F. (2022). The first record of the invasive Chinese pond mussel *Sinanodonta woodiana* (Lea, 1834) (Bivalvia: Unionidae) in the African continent. Natura Croatica 31, 393–398.

[brv70058-bib-0287] Madzivanzira, T. C. , South, J. , Wood, L. E. , Nunes, A. L. & Weyl, O. L. F. (2021). A review of freshwater crayfish introductions in Africa. Reviews in Fisheries Science & Aquaculture 29, 218–241.

[brv70058-bib-0288] Magliozzi, C. , Artois, M. , Bertaccini, A. , Candresse, T. , Tsiamis, K. , D'Amico, F. , Deriu, I. , Gervasini, E. & Cardoso, A. C. (2022). European primary datasets of alien bacteria and viruses. Scientific Data 9, 403. 35831307 10.1038/s41597-022-01485-1 PMC9279316

[brv70058-bib-0289] Magory Cohen, T. , McKinney, M. , Kark, S. & Dor, R. (2019). Global invasion in progress: modeling the past, current and potential global distribution of the common myna. Biological Invasions 21, 1295–1309.

[brv70058-bib-0290] Makhkamov, T. , Kortz, A. , Hejda, M. , Brundu, G. & Pyšek, P. (2024). Naturalized alien flora of Uzbekistan: species richness, origin and habitats. Biological Invasions 26, 2819–2830.

[brv70058-bib-0291] Manaaki Whenua — Landcare Research (2024). Biota of New Zealand . https://biotaNZ.landcareresearch.co.nz.

[brv70058-bib-0292] Marchioro, C. A. & Krechemer, F. S. (2018). Potential global distribution of *Diabrotica* species and the risks for agricultural production. Pest Management Science 74, 2100–2109. 10.1002/ps.4906 29575502

[brv70058-bib-0293] Maroyi, A. (2012). The casual, naturalised and invasive alien flora of Zimbabwe based on herbarium and literature records. Koedoe 54, a1054.

[brv70058-bib-0294] Marr, S. M. , Olden, J. D. , Leprieur, F. , Arismendi, I. , Ćaleta, M. , Morgan, D. L. , Nocita, A. , Šanda, R. , Serhan Tarkan, A. & García‐Berthou, E. (2013). A global assessment of freshwater fish introductions in mediterranean‐climate regions. Hydrobiologia 719, 317–329.

[brv70058-bib-0295] Martin, L. J. , Blossey, B. & Ellis, E. (2012). Mapping where ecologists work: biases in the global distribution of terrestrial ecological observations. Frontiers in Ecology and the Environment 10, 195–201.

[brv70058-bib-0296] Masocha, M. & Dube, T. (2018). Global terrestrial biomes at risk of cacti invasion identified for four species using consensual modelling. Journal of Arid Environments 156, 77–86.

[brv70058-bib-0297] Mateo, J. A. , Ayres, C. & López‐Jurado, L. F. (2011). Los anfibios y reptiles naturalizados en España. Historia y evolución de una problemática creciente. Boletín de la Asociación Herpetológica Española 22, 2–42.

[brv70058-bib-0298] Mattson, W. J. J. , Niemela, P. , Millers, I. & Inguanzo, Y. (1994). Immigrant Phytophagous Insects on Woody Plants in the United States and Canada: an Annotated List. US Department of Agriculture, St. Paul.

[brv70058-bib-0299] McCarthy, A. H. , Peck, L. S. , Hughes, K. A. & Aldridge, D. C. (2019). Antarctica: the final frontier for marine biological invasions. Global Change Biology 25, 2221–2241. 31016829 10.1111/gcb.14600 PMC6849521

[brv70058-bib-0300] McCook, S. (2006). Global rust belt: *Hemileia vastatrix* and the ecological integration of world coffee production since 1850. Journal of Global History 1, 177–195.

[brv70058-bib-0301] McGeoch, M. A. , Buba, Y. , Arlé, E. , Belmaker, J. , Clarke, D. A. , Jetz, W. , Li, R. , Seebens, H. , Essl, F. , Groom, Q. , García‐Berthou, E. , Lenzner, B. , Meyer, C. , Vicente, J. R. , Wilson, J. R. U. , *et al*. (2023). Invasion trends: an interpretable measure of change is needed to support policy targets. Conservation Letters 16, e12981.

[brv70058-bib-0302] McGeoch, M. A. , Shaw, J. D. , Terauds, A. , Lee, J. E. & Chown, S. L. (2015). Monitoring biological invasion across the broader Antarctic: a baseline and indicator framework. Global Environmental Change 32, 108–125.

[brv70058-bib-0303] McPherson, B. A. , Mori, S. R. , Wood, D. L. , Storer, A. J. , Svihra, P. , Kelly, N. M. & Standiford, R. B. (2005). Sudden oak death in California: disease progression in oaks and tanoaks. Forest Ecology and Management 213, 71–89.

[brv70058-bib-0304] Mead, A. , Carlton, J. T. , Griffiths, C. L. & Rius, M. (2011). Revealing the scale of marine bioinvasions in developing regions: a South African re‐assessment. Biological Invasions 13, 1991–2008.

[brv70058-bib-0305] Measey, J. , Hui, C. & Somers, M. J. (2020). Terrestrial vertebrate invasions in South Africa. In Biological Invasions in South Africa (eds B. W. van Wilgen , J. Measey , D. M. Richardson , J. R. Wilson and T. A. Zengeya ), pp. 115–151. Springer, Cham.

[brv70058-bib-0306] Medvecká, J. , Kliment, J. , Májeková, J. , Halada, Ľ. , Zaliberová, M. , Gojdičová, E. , Feráková, V. & Jarolímek, I. (2012). Inventory of the alien flora of Slovakia. Preslia 84, 257–309.

[brv70058-bib-0307] Meshaka, W. E. Jr. (2011). A runaway train in the making: the exotic amphibians, reptiles, turtles, and crocodilians of Florida. Herpetological Conservation and Biology 6, 1–101.

[brv70058-bib-0308] Meyer, C. , Kreft, H. , Guralnick, R. & Jetz, W. (2015). Global priorities for an effective information basis of biodiversity distributions. Nature Communications 6, 8221. 10.1038/ncomms9221 PMC4569846 26348291

[brv70058-bib-0309] Meyer, J.‐Y. & Florence, J. (1996). Tahiti's native flora endangered by the invasion of *Miconia calvescens* DC. (Melastomataceae). Journal of Biogeography 23, 775–781.

[brv70058-bib-0310] Meyerson, L. A. , Pauchard, A. , Brundu, G. , Carlton, J. T. , Hierro, J. L. , Kueffer, C. , Pandit, M. K. , Pyšek, P. , Richardson, D. M. & Packer, J. G. (2022). Moving toward global strategies for managing invasive alien species. In Global Plant Invasions (eds D. R. Clements , M. K. Upadhyaya , S. Joshi and A. Shrestha ), pp. 331–360. Springer, Cham.

[brv70058-bib-0311] Meyerson, L. A. , Cronin, J. T. , Packer, J. , Pyšek, P. & Saltonstall, K. (2025). Ecology and evolution of *Phragmites australis*, one of the world's most successful plant species. Annual Review of Ecology, Evolution, and Systematics 56, 73–98.

[brv70058-bib-0312] Mghili, B. , Lamine, I. , Rami Laamraoui, M. , Aksissou, M. & Galanidi, M. (2024). Updating the national list of marine alien species in Morocco. Mediterranean Marine Science 25, 231–249.

[brv70058-bib-0313] Mills, E. L. , Leach, J. H. , Carlton, J. T. & Secor, C. L. (1993). Exotic species in the Great Lakes: a history of biotic crises and anthropogenic introductions. Journal of Great Lakes Research 19, 1–54.

[brv70058-bib-0314] Monteiro, M. , Reino, L. , Schertler, A. , Essl, F. , Figueira, R. , Ferreira, M. & Capinha, C. (2020). A database of the global distribution of alien macrofungi. Biodiversity Data Journal 8, e51459. 32280297 10.3897/BDJ.8.e51459 PMC7142166

[brv70058-bib-0315] Moser, D. , Lenzner, B. , Weigelt, P. , Dawson, W. , Kreft, H. , Pergl, J. , Pyšek, P. , van Kleunen, M. , Winter, M. , Capinha, C. , Cassey, P. , Dullinger, S. , Economo, E. P. , García‐Díaz, P. , Guénard, B. , *et al*. (2018). Remoteness promotes biological invasions on islands worldwide. Proceedings of the National Academy of Sciences 115, 9270–9275. 10.1073/pnas.1804179115 PMC6140508 30158167

[brv70058-bib-0316] Moyle, P. B. (1986). Fish introductions into North America: patterns and ecological impact. In Ecology of Biological Invasions of North America and Hawaii (eds H. A. Mooney and J. A. Drake ), pp. 27–43. Springer, New York.

[brv70058-bib-0317] Mrugała, A. , Kozubíková‐Balcarová, E. , Chucholl, C. , Cabanillas Resino, S. , Viljamaa‐Dirks, S. , Vukić, J. & Petrusek, A. (2015). Trade of ornamental crayfish in Europe as a possible introduction pathway for important crustacean diseases: crayfish plague and white spot syndrome. Biological Invasions 17, 1313–1326.

[brv70058-bib-0318] Muñoz‐Mas, R. , Essl, F. , van Kleunen, M. , Seebens, H. , Dawson, W. , Casal, C. M. V. & García‐Berthou, E. (2023). Two centuries of spatial and temporal dynamics of freshwater fish introductions. Global Ecology and Biogeography 32, 1632–1644.

[brv70058-bib-0319] Muñoz‐Mas, R. & García‐Berthou, E. (2020). Alien animal introductions in Iberian inland waters: an update and analysis. Science of the Total Environment 703, 134505. 31734502 10.1016/j.scitotenv.2019.134505

[brv70058-bib-0320] Nahrung, H. F. & Carnegie, A. J. (2020). Non‐native forest insects and pathogens in Australia: establishment, spread, and impact. Frontiers in Forests and Global Change 3, 37.

[brv70058-bib-0321] Nealis, V. G. , Demerchant, I. , Langor, D. , Noseworthy, M. K. , Pohl, G. , Porter, K. , Shanks, E. , Turnquist, R. & Waring, V. (2016). Historical occurrence of alien arthropods and pathogens on trees in Canada. Canada Journal of Forest Research 180, 172–180.

[brv70058-bib-0322] Nentwig, W. , Bacher, S. , Kumschick, S. , Pyšek, P. & Vilà, M. (2018). More than “100 worst” alien species in Europe. Biological Invasions 20, 1611–1621.

[brv70058-bib-0323] Newman, J. , Poirot, C. , Roper‐Gee, R. , Leihy, R. I. & Chown, S. L. (2018). A decade of invertebrate colonization pressure on Scott Base in the Ross Sea region. Biological Invasions 20, 2623–2633.

[brv70058-bib-0324] Ng, T. H. , Tan, S. K. , Wong, W. H. , Meier, R. , Chan, S.‐Y. , Tan, H. H. & Yeo, D. C. J. (2016). Molluscs for sale: assessment of freshwater gastropods and bivalves in the ornamental pet trade. PLoS One 11, e0161130. 27525660 10.1371/journal.pone.0161130 PMC4985174

[brv70058-bib-0325] Nhoybouakong, M. & Khamphouke, K. (2003). Laos. In Invasive Alien Species in South‐Southeast Asia: National Reports & Directory of Resources (eds N. Pallewatta , J. K. Reaser and A. T. Gutierrez ), pp. 33–42. Cape Town, Global Invasive Species Programme.

[brv70058-bib-0326] Nishida, G. M. (ed.) (2002). Hawaiian Terrestrial Arthropod Checklist, Fourth Edition. Bishop Museum, Honolulu.

[brv70058-bib-0327] Njoroge, A. W. , Andersson, B. , Lees, A. K. , Mutai, C. , Forbes, G. A. , Yuen, J. E. & Pelle, R. (2019). Genotyping of *Phytophthora* infestans in eastern Africa reveals a dominating invasive European lineage. Phytopathology 109, 670–680. 30253119 10.1094/PHYTO-07-18-0234-R

[brv70058-bib-0328] Norman, P. E. , Johnny, J. , Moiforay, S. K. & Norman, Y. S. G. E. (2021). Invasive alien species of Sierra Leone. In Invasive Alien Species, First Edition (eds T. Pullaiah and M. R. Ielmini ), pp. 242–262. John Wiley & Sons, Hoboken.

[brv70058-bib-0329] Novoa, A. , González, L. , Moravcová, L. & Pyšek, P. (2013). Constraints to native plant species establishment in coastal dune communities invaded by *Carpobrotus edulis*: implications for restoration. Biological Conservation 164, 1–9.

[brv70058-bib-0330] Nunes, A. L. , Tricarico, E. , Panov, V. E. , Cardoso, A. C. & Katsanevakis, S. (2015). Pathways and gateways of freshwater invasions in Europe. Aquatic Invasions 10, 359–370.

[brv70058-bib-0331] Nuñez, M. A. , Chiuffo, M. C. , Seebens, H. , Kuebbing, S. , McCary, M. A. , Lieurance, D. , Zhang, B. , Simberloff, D. & Meyerson, L. A. (2021). Two decades of data reveal that Biological invasions needs to increase participation beyond North America, Europe, and Australasia. Biological Invasions 24, 333–340.

[brv70058-bib-0332] Nyári, Á. , Ryall, C. & Peterson, A. T. (2006). Global invasive potential of the house crow *Corvus splendens* based on ecological niche modelling. Journal of Avian Biology 37, 306–311.

[brv70058-bib-0333] O'Dowd, D. J. , Green, P. T. & Lake, P. S. (2003). Invasional ‘meltdown’ on an oceanic Island. Ecology Letters 6, 812–817.

[brv70058-bib-0334] Oguz, T. , Fach, B. & Salihoglu, B. (2008). Invasion dynamics of the alien ctenophore *Mnemiopsis leidyi* and its impact on anchovy collapse in the Black Sea. Journal of Plankton Research 30, 1385–1397.

[brv70058-bib-0335] Oldstone, M. B. A. (2020). Viruses, Plagues, and History: Past, Present, and Future. Oxford University Press, New York.

[brv70058-bib-0336] Ööpik, M. , Kukk, T. , Kull, K. & Kull, T. (2008). The importance of human mediation in species establishment: analysis of the alien flora of Estonia. Boreal Environment Research 13, 53–67.

[brv70058-bib-0337] Osborne, M. A. (2000). Acclimatizing the world: a history of the paradigmatic colonial science. Osiris 15, 135–151. 11971295 10.1086/649323

[brv70058-bib-0338] Osyczka, P. (2010). Alien lichens unintentionally transported to the “Arctowski” station (South Shetlands, Antarctica). Polar Biology 33, 1067–1073.

[brv70058-bib-0339] Osyczka, P. , Mleczko, P. , Karasiński, D. & Chlebicki, A. (2012). Timber transported to Antarctica: a potential and undesirable carrier for alien fungi and insects. Biological Invasions 14, 15–20.

[brv70058-bib-0340] Oualid, J. A. , Iazza, B. , Tamsouri, N. M. , El Aamri, F. , Moukrim, A. & López‐González, P. J. (2019). Hidden diversity under morphology‐based identifications of widespread invasive species: the case of the ‘well‐known’ hydromedusa *Craspedacusta sowerbii* Lankester 1880. Animal Biodiversity and Conservation 42, 301–316.

[brv70058-bib-0341] Ounifi‐Ben Amor, K. , Rifi, Μ. , Ghanem, R. , Draeif, I. , Zaouali, J. & Ben Souissi, J. (2015). Update of alien fauna and new records from Tunisian marine waters. Mediterranean Marine Science 17, 124.

[brv70058-bib-0342] Outinen, O. , Katajisto, T. , Nygård, H. , Puntila‐Dodd, R. & Lehtiniemi, M. (2024). National assessment on the status, trends and impacts of marine non‐indigenous species for the European Union marine strategy framework directive. Ecological Indicators 158, 111593.

[brv70058-bib-0343] Packer, J. G. , Meyerson, L. A. , Richardson, D. M. , Brundu, G. , Allen, W. J. , Bhattarai, G. P. , Brix, H. , Canavan, S. , Castiglione, S. , Cicatelli, A. , Čuda, J. , Cronin, J. T. , Eller, F. , Guarino, F. , Guo, W.‐H. , *et al*. (2017). Global networks for invasion science: benefits, challenges and guidelines. Biological Invasions 19, 1081–1096.

[brv70058-bib-0344] Pagad, S. , Bisset, S. , Genovesi, P. , Groom, Q. , Hirsch, T. , Jetz, W. , Ranipeta, A. , Schigel, D. , Sica, Y. V. & McGeoch, M. A. (2022). Country compendium of the global register of introduced and invasive species. Scientific Data 9, 391. 35810161 10.1038/s41597-022-01514-z PMC9271038

[brv70058-bib-1344] Pant, V. , Patwardhan, C. , Patil, K. , Bhowmick, A. R. , Mukherjee, A. , & Banerjee, A. K. (2021). ILORA: A database of alien vascular flora of India. Ecological Solutions and Evidence 2, e312105.

[brv70058-bib-0345] Paulay, G. , Kirkendale, L. , Lambert, G. & Meyer, C. (2002). Anthropogenic biotic interchange in a coral reef ecosystem: a case study from Guam. Pacific Science 56, 403–422.

[brv70058-bib-0346] Pederson, J. A. , Gollasch, S. , Laing, I. , McCollin, T. , Miossec, L. , Occhipinti‐Ambrogi, A. , Wallentinus, I. & Werner, M. (2017). Status of introductions of non‐indigenous marine species to the North Atlantic and adjacent waters 2003–2007. (Volume 334). ICES Cooperative Research Report, Copenhagen, Denmark. 10.17895/ices.pub.1977.

[brv70058-bib-0347] Pelser, P. B. , Barcelona, J. F. & Nickrent, D. L. (2011). Co's Digital Flora of the Philippines. http://www.philippineplants.org.

[brv70058-bib-0348] Peltanová, A. , Petrusek, A. , Kment, P. & Juřičková, L. (2012). A fast snail's pace: colonization of Central Europe by Mediterranean gastropods. Biological Invasions 14, 759–764.

[brv70058-bib-0349] Perella, C. D. & Behm, J. E. (2020). Understanding the spread and impact of exotic geckos in the greater Caribbean region. Biodiversity and Conservation 29, 1109–1134.

[brv70058-bib-0350] Pérez, G. , Vilà, M. & Gallardo, B. (2022). Potential impact of four invasive alien plants on the provision of ecosystem services in Europe under present and future climatic scenarios. Ecosystem Services 56, 101459.

[brv70058-bib-0351] Pertierra, L. R. , Hughes, K. A. , Tejedo, P. , Enriquez, N. , Lucianez, M. J. & Benayas, J. (2017). Eradication of the non‐native *Poa pratensis* colony at Cierva point, Antarctica: a case study of international cooperation and practical management in an area under multi‐party governance. Environmental Science & Policy 69, 50–56.

[brv70058-bib-0352] Peters, K. , Sink, K. & Robinson, T. (2017). Raising the flag on marine alien fouling species. Management of Biological Invasions 8, 1–11.

[brv70058-bib-0353] Petsch, D. K. , Ribas, L. G. D. S. , Mantovano, T. , Pulzatto, M. M. , Alves, A. T. , Pinha, G. D. & Thomaz, S. M. (2021). Invasive potential of golden and zebra mussels in present and future climatic scenarios in the new world. Hydrobiologia 848, 2319–2330.

[brv70058-bib-0354] Picker, M. D. & Griffiths, C. L. (2017). Alien animals in South Africa – composition, introduction history, origins and distribution patterns. Bothalia 47, a2147.

[brv70058-bib-0355] Pickering, C. M. , Bear, R. & Hill, W. (2007). Indirect impacts of nature based tourism and recreation: the association between infrastructure and the diversity of exotic plants in Kosciuszko National Park, Australia. Journal of Ecotourism 6, 146–157.

[brv70058-bib-0356] Pili, A. N. , Tingley, R. , Sy, E. Y. , Diesmos, M. L. L. & Diesmos, A. C. (2020). Niche shifts and environmental non‐equilibrium undermine the usefulness of ecological niche models for invasion risk assessments. Scientific Reports 10, 7972. 32409706 10.1038/s41598-020-64568-2 PMC7224218

[brv70058-bib-0357] Pipek, P. , Blackburn, T. M. & Pyšek, P. (2019). The ins and outs of acclimatisation: imports versus translocations of skylarks and starlings in 19th century New Zealand. Biological Invasions 21, 1395–1413.

[brv70058-bib-0358] Pipek, P. , Pyšek, P. & Blackburn, T. M. (2015). How the yellowhammer became a kiwi: the history of an alien bird invasion revealed. NeoBiota 24, 1–31.

[brv70058-bib-0359] Pitcher, T. J. & Hart, P. J. B. (eds) (1995). The Impact of Species Changes in African Lakes. Springer, Dordrecht.

[brv70058-bib-0360] Pleguezuelos, J. M. (2002). Las especies introducidas de anfibios y reptiles. In Atlas y Libro Rojo de Los Anfibios y Reptiles de España (eds J. M. Pleguezuelos , R. Márquez and M. Lizana ), pp. 501–532. Dirección General de Conservación de la Naturaleza‐Asociación Herpetológica Española, Madrid.

[brv70058-bib-0361] Pluess, T. , Cannon, R. , Jarošík, V. , Pergl, J. , Pyšek, P. & Bacher, S. (2012 *a*). When are eradication campaigns successful? A test of common assumptions. Biological Invasions 14, 1365–1378.

[brv70058-bib-0362] Pluess, T. , Jarošík, V. , Pyšek, P. , Cannon, R. , Pergl, J. , Breukers, A. & Bacher, S. (2012 *b*). Which factors affect the success or failure of eradication campaigns against alien species? PLoS One 7, e48157. 23110197 10.1371/journal.pone.0048157 PMC3482215

[brv70058-bib-0363] Png‐Gonzalez, L. , Comas‐González, R. , Calvo‐Manazza, M. , Follana‐Berná, G. , Ballesteros, E. , Díaz‐Tapia, P. , Falcón, J. M. , García Raso, J. E. , Gofas, S. , González‐Porto, M. , López, E. , Ramos‐Esplá, A. A. , Velasco, E. & Carbonell, A. (2023). Updating the national baseline of non‐indigenous species in Spanish marine waters. Diversity 15, 630.

[brv70058-bib-0364] Policelli, N. , Bruns, T. D. , Vilgalys, R. & Nuñez, M. A. (2019). Suilloid fungi as global drivers of pine invasions. New Phytologist 222, 714–725. 30586169 10.1111/nph.15660

[brv70058-bib-0365] Powell, R. , Henderson, R. W. , Farmer, M. C. , Breuil, M. , Echternacht, A. C. , van Buurt, G. , Romagosa, C. M. & Perry, G. (2011). Introduced amphibians and reptiles in the greater Caribbean: patterns and conservation implications. In Conservation of Caribbean Island Herpetofaunas (eds A. Hailey , B. S. Wilson and J. A. Horrocks ), pp. 63–143. Brill, Leiden, The Netherlands.

[brv70058-bib-0366] Preston, C. D. , Pearman, D. A. & Dines, T. D. (2002). New Atlas of the British and Irish Flora. Oxford University Press, Oxford.

[brv70058-bib-0367] Protopopova, V. V. & Shevera, M. V. (2014). Ergasiophytes of the Ukrainian flora. Biodiversity Research and Conservation 35, 31–46.

[brv70058-bib-0368] Pyšek, P. , Chytrý, M. , Pergl, J. , Sádlo, J. & Wild, J. (2012a). Plant invasions in The Czech Republic: current state, introduction dynamics, invasive species and invaded habitats. Preslia 84, 575–629.

[brv70058-bib-0369] Pyšek, P. , Dawson, W. , Essl, F. , Kreft, H. , Pergl, J. , Seebens, H. , van Kleunen, M. , Weigelt, P. & Winter, M. (2019). Contrasting patterns of naturalized plant richness in the Americas: numbers are higher in the north but expected to rise sharply in the South. Global Ecology and Biogeography 28, 779–783.

[brv70058-bib-0370] Pyšek, P. , Hulme, P. E. , Simberloff, D. , Bacher, S. , Blackburn, T. M. , Carlton, J. T. , Dawson, W. , Essl, F. , Foxcroft, L. C. , Genovesi, P. , Jeschke, J. M. , Kühn, I. , Liebhold, A. M. , Mandrak, N. E. , Meyerson, L. A. , *et al*. (2020). Scientists' warning on invasive alien species. Biological Reviews 95, 1511–1534. 32588508 10.1111/brv.12627 PMC7687187

[brv70058-bib-0371] Pyšek, P. , Jarošík, V. , Hulme, P. E. , Pergl, J. , Hejda, M. , Schaffner, U. & Vilà, M. (2012 *b*). A global assessment of invasive plant impacts on resident species, communities and ecosystems: the interaction of impact measures, invading species' traits and environment. Global Change Biology 18, 1725–1737.

[brv70058-bib-0372] Pyšek, P. , Pergl, J. , Essl, F. , Lenzner, B. , Dawson, W. , Kreft, H. , Weigelt, P. , Winter, M. , Kartesz, J. , Nishino, M. , Antonova, L. A. , Barcelona, J. F. , Cabezas, F. J. , Cárdenas, D. , Cárdenas‐Toro, J. , *et al*. (2017). Naturalized alien flora of the world: species diversity, taxonomic and phylogenetic patterns, geographic distribution and global hotspots of plant invasion. Preslia 89, 203–274.

[brv70058-bib-0373] Pyšek, P. , Richardson, D. M. , Pergl, J. , Jarošík, V. , Sixtová, Z. & Weber, E. (2008). Geographical and taxonomic biases in invasion ecology. Trends in Ecology & Evolution 23, 237–244. 18367291 10.1016/j.tree.2008.02.002

[brv70058-bib-0374] Pyšek, P. , Sádlo, J. , Chrtek, J. , Chytrý, M. , Kaplan, Z. , Pergl, J. , Pokorná, A. , Axmanová, I. , Čuda, J. , Doležal, J. , Dřevojan, P. , Hejda, M. , Kočár, P. , Kortz, A. , Lososová, Z. , *et al*. (2022). Catalogue of alien plants of The Czech Republic (3rd edition). Preslia 94, 447–577.

[brv70058-bib-0375] Rabitsch, W. & Nehring, S. (2017). *Naturschutzfachliche Invasivitätsbewertungen für in Deutschland Wild Lebende Gebietsfremde Aquatische Pilze, Niedere Pflanzen und Wirbellose Tiere*. Bundesamt für Naturschutz (BfN‐Skripten 458), Bonn.

[brv70058-bib-0376] Randall, R. P. (2002). A Global Compendium of Weeds. R. G. and F. J. Richardson, Melbourne.

[brv70058-bib-0377] Raulerson, L. (2006). Checklist of plants of the Mariana Islands. University of Guam Herbarium Contribution 38, 1–69.

[brv70058-bib-0378] Reaser, J. K. , Meyerson, L. A. , Cronk, Q. , De Poorter, M. , Eldrege, L. G. , Green, E. , Kairo, M. , Latasi, P. , Mack, R. N. , Mauremootoo, J. , O'Dowd, D. , Orapa, W. , Sastroutomo, S. , Saunders, A. & Shine, C. (2007). Ecological and socioeconomic impacts of invasive alien species in Island ecosystems. Environmental Conservation 34, 98–111.

[brv70058-bib-0379] Reino, L. , Figueira, R. , Beja, P. , Araújo, M. B. , Capinha, C. & Strubbe, D. (2017). Networks of global bird invasion altered by regional trade ban. Science Advances 3, e1700783. 29181443 10.1126/sciadv.1700783 PMC5699901

[brv70058-bib-0380] Ricciardi, A. (2001). Facilitative interactions among aquatic invaders: is an ‘invasional meltdown’ occurring in the Great Lakes? Canadian Journal of Fisheries and Aquatic Sciences 58, 2513–2525.

[brv70058-bib-0381] Ricciardi, A. (2006). Patterns of invasion in the Laurentian Great Lakes in relation to changes in vector activity. Diversity and Distributions 12, 425–433.

[brv70058-bib-0382] Ricciardi, A. , Blackburn, T. M. , Carlton, J. T. , Dick, J. T. A. , Hulme, P. E. , Iacarella, J. C. , Jeschke, J. M. , Liebhold, A. M. , Lockwood, J. L. , MacIsaac, H. J. , Pyšek, P. , Richardson, D. M. , Ruiz, G. M. , Simberloff, D. , Sutherland, W. J. , *et al*. (2017). Invasion science: looking forward rather than revisiting old ground. Trends in Ecology & Evolution 32, 809–810. 28863858 10.1016/j.tree.2017.08.007

[brv70058-bib-0383] Ricciardi, A. & MacIsaac, H. J. (2000). Recent mass invasion of the north American Great Lakes by Ponto‐Caspian species. Trends in Ecology & Evolution 15, 62–65. 10652557 10.1016/s0169-5347(99)01745-0

[brv70058-bib-0384] Ricciardi, A. & MacIsaac, H. J. (2022). Vector control reduces the rate of species invasion in the world's largest freshwater ecosystem. Conservation Letters 15, e12866.

[brv70058-bib-0385] Richardson, D. M. , Binggeli, P. & Botella, C. (2023). Australian *acacia* species in Africa. In Wattles: Australian Acacia Species around the World (eds D. M. Richardson , J. J. le Roux and E. Marchante ), pp. 181–200. CAB International, Wallingford.

[brv70058-bib-0386] Richardson, D. M. , Foxcroft, L. C. , Latombe, G. , Le Maitre, D. C. , Rouget, M. & Wilson, J. R. (2020). The biogeography of South African terrestrial plant invasions. In Biological Invasions in South Africa (eds B. W. van Wilgen , J. Measey , D. M. Richardson , J. R. Wilson and T. A. Zengeya ), pp. 67–96. Springer, Cham.

[brv70058-bib-0387] Richardson, B. A. , Kim, M. S. , Klopfenstein, N. B. , Ota, Y. , Woo, K. S. & Hamelin, R. C. (2009). Tracking the footsteps of an invasive plant pathogen: intercontinental phylogeographic structure of the white‐pine‐blister‐rust fungus, *Cronartium ribicola* . In Breeding and Genetic Resources of Five‐Needle Pines: Ecophysiology, Disease Resistance and Developmental Biology, Proceedings of the Conference, Yangyang, Korea 22–26 September 2008 (eds D. Noshad , E. W. Noh , J. King and R. A. Sniezko ), pp. 56–60. Korea Forest Research Institute, Seoul.

[brv70058-bib-0388] Richardson, D. M. , Pyšek, P. , Rejmánek, M. , Barbour, M. G. , Panetta, F. D. & West, C. J. (2000). Naturalization and invasion of alien plants: concepts and definitions. Diversity and Distributions 6, 93–107.

[brv70058-bib-0389] Richardson, D. M. , Witt, A. B. R. , Pergl, J. , Dawson, W. , Essl, F. , Kreft, H. , van Kleunen, M. , Weigelt, P. , Winter, M. & Pyšek, P. (2022). Plant invasions in Africa. In Global Plant Invasions (eds D. R. Clements , M. K. Upadhyaya , S. Joshi and A. Shrestha ), pp. 225–252. Springer, Cham.

[brv70058-bib-0390] Riegl, B. , Walentowitz, A. , Sevilla, C. , Chango, R. & Jäger, H. (2023). Invasive blackberry outcompetes the endemic Galapagos tree daisy *Scalesia pedunculata* . Ecological Applications 33, e2846. 36932847 10.1002/eap.2846

[brv70058-bib-0391] Rigling, D. & Prospero, S. (2018). *Cryphonectria parasitica*, the causal agent of chestnut blight: invasion history, population biology and disease control. Molecular Plant Pathology 19, 7–20. 28142223 10.1111/mpp.12542 PMC6638123

[brv70058-bib-0392] Robinson, T. B. , Peters, K. & Brooker, B. (2020). Coastal invasions: the South African context. In Biological Invasions in South Africa (eds B. W. van Wilgen , J. Measey , D. M. Richardson , J. R. Wilson and T. A. Zengeya ), pp. 229–247. Springer, Cham.

[brv70058-bib-0393] Rogers, H. S. , Buhle, E. R. , HilleRisLambers, J. , Fricke, E. C. , Miller, R. H. & Tewksbury, J. J. (2017). Effects of an invasive predator cascade to plants via mutualism disruption. Nature Communications 8, 14557. 10.1038/ncomms14557 PMC5344968 28270682

[brv70058-bib-0394] Rojas‐Sandoval, J. & Acevedo‐Rodríguez, P. (2015). Naturalization and invasion of alien plants in Puerto Rico and the Virgin Islands. Biological Invasions 17, 149–163.

[brv70058-bib-0395] Roll, U. , Dayan, T. , Simberloff, D. & Mienis, H. K. (2009). Non‐indigenous land and freshwater gastropods in Israel. Biological Invasions 11, 1963–1972.

[brv70058-bib-0396] Roques, A. , Auger‐Rozenberg, M. A. , Blackburn, T. M. , Garnas, J. , Pyšek, P. , Rabitsch, W. , Richardson, D. M. , Wingfield, M. J. , Liebhold, A. M. & Duncan, R. P. (2016). Temporal and interspecific variation in rates of spread for insect species invading Europe during the last 200 years. Biological Invasions 18, 907–920.

[brv70058-bib-0397] Roy, H. E. , Bacon, J. , Beckmann, B. , Harrower, C. A. , Hill, M. O. , Isaac, N. J. B. , Preston, C. D. , Rathod, B. , Rorke, S. L. , Marchant, J. H. , Musgrove, A. , Noble, D. , Sewell, J. , Seeley, B. , Sweet, N. , *et al*. (2012). Non‐Native Species in Great Britain: Establishment. Detection and Reporting to Inform Effective Decision Making, Non‐Native Species Secretariat, York.

[brv70058-bib-0398] Roy, H. E. , Rabitsch, W. , Scalera, R. , Stewart, A. , Gallardo, B. , Genovesi, P. , Essl, F. , Adriaens, T. , Bacher, S. , Booy, O. , Branquart, E. , Brunel, S. , Copp, G. H. , Dean, H. , D'hondt, B. , *et al*. (2018). Developing a framework of minimum standards for the risk assessment of alien species. Journal of Applied Ecology 55, 526–538.

[brv70058-bib-0399] Ruffino, L. , Bourgeois, K. , Vidal, E. , Duhem, C. , Paracuellos, M. , Escribano, F. , Sposimo, P. , Baccetti, N. , Pascal, M. & Oro, D. (2009). Invasive rats and seabirds after 2,000 years of an unwanted coexistence on Mediterranean islands. Biological Invasions 11, 1631–1651.

[brv70058-bib-0400] Ruiz, G. M. , Fofonoff, P. , Steves, B. & Dahlstrom, A. (2011). Marine crustacean invasions in North America: a synthesis of historical records and documented impacts. In In the Wrong Place – Alien Marine Crustaceans: Distribution, Biology and Impacts (eds B. S. Galil , P. F. Clark and J. T. Carlton ), pp. 215–250. Springer, Dordrecht.

[brv70058-bib-0401] Ruiz, G. M. , Fofonoff, P. W. , Carlton, J. T. , Wonham, M. J. & Hines, A. H. (2000). Invasion of coastal marine communities in North America: apparent patterns, processes, and biases. Annual Review of Ecology, Evolution, and Systematics 31, 481–531.

[brv70058-bib-0402] Russell, J. C. , Cole, N. C. , Zuël, N. & Rocamora, G. (2016). Introduced mammals on Western Indian Ocean islands. Global Ecology and Conservation 6, 132–144.

[brv70058-bib-0403] Russell, J. C. & Kueffer, C. (2019). Island biodiversity in the Anthropocene. Annual Review of Environment and Resources 44, 31–60.

[brv70058-bib-0404] Russell, J. C. & Le Corre, M. (2009). Introduced mammal impacts on seabirds in the Îles Éparses, western Indian Ocean. Marine Ornithology 37, 121–128.

[brv70058-bib-0405] Russell, J. C. , Meyer, J. Y. , Holmes, N. D. & Pagad, S. (2017). Invasive alien species on islands: impacts, distribution, interactions and management. Environmental Conservation 44, 359–370.

[brv70058-bib-0406] Rwomushana, I. , Beale, T. , Chipabika, G. , Day, R. , Gonzalez‐Moreno, P. , Lamontagne‐Godwin, J. , Makale, F. , Pratt, C. & Tambo, J. (2019). *Tomato Leafminer (*Tuta absoluta*): Impacts and Coping Strategies for Africa*. CABI Working Paper 12.

[brv70058-bib-0407] Ryan, S. F. , Lombaert, E. , Espeset, A. , Vila, R. , Talavera, G. , Dinca, V. , Doellman, M. M. , Renshaw, M. A. , Eng, M. W. , Hornett, E. A. , Li, Y. , Pfrender, M. E. & Shoemaker, D. (2019). Global invasion history of the agricultural pest butterfly *Pieris rapae* revealed with genomics and citizen science. Proceedings of the National Academy of Sciences 116, 20015–20024. 10.1073/pnas.1907492116 PMC6778179 31506352

[brv70058-bib-0408] Saba, A. O. , Ismail, A. , Zulkifli, S. Z. , Shohaimi, S. , Jamil, N. R. , Nawi, N. M. , Ghani, I. F. A. , Halim, M. R. A. & Amal, M. N. A. (2020). Checklists, production trends, and potential ecological and socioeconomic impacts of non‐native freshwater fishes in Malaysia: a review. Aquatic Invasions 15, 646–670.

[brv70058-bib-0409] Saltonstall, K. (2002). Cryptic invasion by a non‐native genotype of the common reed, *Phragmites australis*, into North America. Proceedings of the National Academy of Sciences 99, 2445–2449. 10.1073/pnas.032477999 PMC122384 11854535

[brv70058-bib-0410] Sandvik, H. , Dolmen, D. , Elven, R. , Falkenhaug, T. , Forsgren, E. , Hansen, H. , Hassel, K. , Husa, V. , Kjærstad, G. , Ødegaard, F. , Pedersen, H. C. , Solheim, H. , Stokke, B. G. , Åsen, P. A. , Åström, S. , *et al*. (2019). Alien plants, animals, fungi and algae in Norway: an inventory of neobiota. Biological Invasions 21, 2997–3012.

[brv70058-bib-0411] Sandvik, H. , Hilmo, O. , Henriksen, S. , Elven, R. , Åsen, P. A. , Hegre, H. , Pedersen, O. , Pedersen, P. A. , Solstad, H. , Vandvik, V. , Westergaard, K. B. , Ødegaard, F. , Åström, S. , Elven, H. , Endrestøl, A. , *et al*. (2020). Alien species in Norway: results from quantitative ecological impact assessments. Ecological Solutions and Evidence 1, e12006.

[brv70058-bib-0412] Sankaran, K. V. & Hussain, K. H. (2019). A Checklist of Fungi Recorded on Eucalyptus Part II. *Research report 559/19*. Kerala Forest Research Institute, Peechi, India.

[brv70058-bib-0413] Sankaran, K. V. & Suresh, T. A. (2013). Invasive alien plants in the forests of Asia and the Pacific. Food and Agriculture Organization of the United Nations Regional Office for Asia and the Pacific, Bangkok, Thailand.

[brv70058-bib-0414] Santini, A. , Ghelardini, L. , De Pace, C. , Desprez‐Loustau, M. L. , Capretti, P. , Chandelier, A. , Cech, T. , Chira, D. , Diamandis, S. , Gaitniekis, T. , Hantula, J. , Holdenrieder, O. , Jankovsky, L. , Jung, T. , Jurc, D. , *et al*. (2013). Biogeographical patterns and determinants of invasion by forest pathogens in Europe. New Phytologist 197, 238–250. 23057437 10.1111/j.1469-8137.2012.04364.x

[brv70058-bib-0415] Scheele, B. C. , Pasmans, F. , Skerratt, L. F. , Berger, L. , Martel, A. , Beukema, W. , Acevedo, A. A. , Burrowes, P. A. , Carvalho, T. , Catenazzi, A. , De la Riva, I. , Fisher, M. C. , Flechas, S. V. , Foster, C. N. , Frías‐Álvarez, P. , *et al*. (2019). Amphibian fungal panzootic causes catastrophic and ongoing loss of biodiversity. Science 363, 1459–1463. 30923224 10.1126/science.aav0379

[brv70058-bib-0416] Schwindt, E. , Battini, N. , Giachetti, C. , Castro, K. & Bortolus, A. (2018). Especies exóticas marino‐costeras: Argentina / Marine‐coastal exotic species: Argentina. Vázquez Mazzini Editores, Buenos Aires.

[brv70058-bib-0417] Schwindt, E. & Bortolus, A. (2017). Aquatic invasion biology research in South America: geographic patterns, advances and perspectives. Aquatic Ecosystem Health & Management 20, 322–333.

[brv70058-bib-0418] Schwindt, E. , Carlton, J. , Orensanz, J. , Scarabino, F. & Bortolus, A. (2020). Past and future of the marine bioinvasions along the southwestern Atlantic. Aquatic Invasions 15, 11–29.

[brv70058-bib-0419] Scott, P. , Bader, M. K.‐F. , Burgess, T. , Hardy, G. & Williams, N. (2019). Global biogeography and invasion risk of the plant pathogen genus *Phytophthora* . Environmental Science & Policy 101, 175–182.

[brv70058-bib-0420] SEAMEO BIOTROP (2003). Penyebaran Jenis Tumbuhan Asing di Indonesia. KLH‐Biotrop, Jakarta.

[brv70058-bib-0421] Seebens, H. , Bacher, S. , Blackburn, T. M. , Capinha, C. , Dawson, W. , Dullinger, S. , Genovesi, P. , Hulme, P. E. , Kleunen, M. , Kühn, I. , Jeschke, J. M. , Lenzner, B. , Liebhold, A. M. , Pattison, Z. , Pergl, J. , *et al*. (2021 *a*). Projecting the continental accumulation of alien species through to 2050. Global Change Biology 27, 970–982. 10.1111/gcb.15333 33000893

[brv70058-bib-0422] Seebens, H. , Blackburn, T. M. , Dyer, E. E. , Genovesi, P. , Hulme, P. E. , Jeschke, J. M. , Pagad, S. , Pyšek, P. , Winter, M. , Arianoutsou, M. , Bacher, S. , Blasius, B. , Brundu, G. , Capinha, C. , Celesti‐Grapow, L. , *et al*. (2017). No saturation in the accumulation of alien species worldwide. Nature Communications 8, 14435. 10.1038/ncomms14435 PMC5316856 28198420

[brv70058-bib-0423] Seebens, H. , Blackburn, T. M. , Hulme, P. E. , Kleunen, M. , Liebhold, A. M. , Orlova‐Bienkowskaja, M. , Pyšek, P. , Schindler, S. & Essl, F. (2021b). Around the world in 500 years: inter‐regional spread of alien species over recent centuries. Global Ecology and Biogeography 30, 1621–1632.

[brv70058-bib-0424] Seebens, H. , Clarke, D. A. , Groom, Q. , Wilson, J. R. U. , García‐Berthou, E. , Kühn, I. , Roigé, M. , Pagad, S. , Essl, F. , Vicente, J. , Winter, M. & McGeoch, M. (2020). A workflow for standardising and integrating alien species distribution data. NeoBiota 59, 39–59.

[brv70058-bib-0425] Seebens, H. , Essl, F. , Dawson, W. , Fuentes, N. , Moser, D. , Pergl, J. , Pyšek, P. , van Kleunen, M. , Weber, E. , Winter, M. & Blasius, B. (2015). Global trade will accelerate plant invasions in emerging economies under climate change. Global Change Biology 21, 4128–4140. 26152518 10.1111/gcb.13021

[brv70058-bib-0426] Seebens, H. , Meyerson, L. A. , Rahlao, S. J. , Lenzner, B. , Tricarico, E. , Aleksanyan, A. , Courchamp, F. , Keskin, E. , Saeedi, H. , Tawake, A. & Pyšek, P. (2023). Chapter 2: trends and status of alien and invasive alien species. In Thematic Assessment Report on Invasive Alien Species and their Control of the Intergovernmental Science‐Policy Platform on Biodiversity and Ecosystem Services (eds H. E. Roy , A. Pauchard , P. Stoett and T. Renard Truong ), p. 190. IPBES Secretariat, Bonn, Germany.

[brv70058-bib-0427] Seebens, H. , Schwartz, N. , Schupp, P. J. & Blasius, B. (2016). Predicting the spread of marine species introduced by global shipping. Proceedings of the National Academy of Sciences 113, 5646–5651. 10.1073/pnas.1524427113 PMC4878520 27091983

[brv70058-bib-0428] Senan, A. S. , Tomasetto, F. , Farcomeni, A. , Somashekar, R. K. & Attorre, F. (2012). Determinants of plant species invasions in an arid Island: evidence from Socotra Island (Yemen). Plant Ecology 213, 1381–1392.

[brv70058-bib-0429] Sghaier, Y. R. , Zakhama‐Sraieb, R. , Benamer, I. & Charfi‐Cheikhrouha, F. (2011). Occurrence of the seagrass *Halophila stipulacea* (Hydrocharitaceae) in the southern Mediterranean Sea. Botanica Marina 54, 575–582.

[brv70058-bib-0430] Shackleton, R. T. , Foxcroft, L. C. , Pyšek, P. , Wood, L. E. & Richardson, D. M. (2020). Assessing biological invasions in protected areas after 30 years: revisiting nature reserves targeted by the 1980s SCOPE programme. Biological Conservation 243, 108424.

[brv70058-bib-0431] Shafer, D. J. , Kaldy, J. E. & Gaeckle, J. L. (2014). Science and management of the introduced seagrass *Zostera japonica* in North America. Environmental Management 53, 147–162. 24100942 10.1007/s00267-013-0172-z

[brv70058-bib-0432] Shafia, A. & Saleem, A. (2003). Maldives. In Invasive Alien Species in South‐Southeast Asia (eds N. Pallewatta , J. K. Reaser and A. T. Gutierrez ), p. 111. National Reports & Directory of Resources. Global Invasive Species Programme, Cape Town, South Africa.

[brv70058-bib-0433] Shaltout, K. H. , Hosni, H. A. , El‐Kady, H. F. , El‐Beheiry, M. A. & Shaltout, S. K. (2016). Composition and pattern of alien species in the Egyptian flora. Flora – Morphology, Distribution, Functional Ecology of Plants 222, 104–110.

[brv70058-bib-0434] Shine, R. (2018). Cane Toad Wars. University of California Press, Oakland.

[brv70058-bib-0435] Shrestha, B. B. (2016). Invasive alien plant species in Nepal. In Frontiers of Botany (eds P. K. Jha , M. Siwakoti and S. Rajbhandary ), pp. 269–284. Tribhuvan University, Kirtipur, Kathmandu, Central Department of Botany.

[brv70058-bib-0436] Simberloff, D. (2011). Non‐natives: 141 scientists object. Nature 475, 36. 10.1038/475036a 21734689

[brv70058-bib-0437] Simberloff, D. , Martin, J.‐L. , Genovesi, P. , Maris, V. , Wardle, D. A. , Aronson, J. , Courchamp, F. , Galil, B. , García‐Berthou, E. , Pascal, M. , Pyšek, P. , Sousa, R. , Tabacchi, E. & Vilà, M. (2013). Impacts of biological invasions: what's what and the way forward. Trends in Ecology & Evolution 28, 58–66. 22889499 10.1016/j.tree.2012.07.013

[brv70058-bib-0438] Simberloff, D. & Rejmánek, M. (eds) (2011). Encyclopedia of Biological Invasions. University of California Press, Berkeley and Los Angeles.

[brv70058-bib-0439] Šimková, A. , Řehulková, E. , Rasoloariniaina, J. R. , Jorissen, M. W. P. , Scholz, T. , Faltýnková, A. , Mašová, Š. & Vanhove, M. P. M. (2019). Transmission of parasites from introduced tilapias: a new threat to endemic Malagasy ichthyofauna. Biological Invasions 21, 803–819.

[brv70058-bib-0440] Simpson, A. , Eyler, M. C. , Guala, G. , Cannister, M. J. , Kozlowsky, N. , Libby, R. & Sellers, E. A. (2018). *A Comprehensive List of Non‐Native Species Established in Three Major Regions of the United States: Version 3.0*. U.S. Geological Survey Data Release. https://www.sciencebase.gov/catalog/item/5b911a5ce4b0702d0e808588.

[brv70058-bib-0441] Skewes, O. , Gonzalez, F. , Olave, R. , Ávila, A. , Vargas, V. , Paulsen, P. & König, H. E. (2006). Abundance and distribution of American beaver, *Castor canadensis* (Kuhl 1820), in Tierra del Fuego and Navarino islands, Chile. European Journal of Wildlife Research 52, 292–296.

[brv70058-bib-0442] Soewarto, J. , Carriconde, F. , Hugot, N. , Bocs, S. , Hamelin, C. & Maggia, L. (2018). Impact of *Austropuccinia psidii* in New Caledonia, a biodiversity hotspot. Forest Pathology 48, e12402.

[brv70058-bib-0443] Soto, I. , Ahmed, D. A. , Beidas, A. , Oficialdegui, F. J. , Tricarico, E. , Angeler, D. G. , Amatulli, G. , Briski, E. , Datry, T. , Dohet, A. , Domisch, S. , England, J. , Feio, M. J. , Forcellini, M. , Johnson, R. K. , *et al*. (2023). Long‐term trends in crayfish invasions across European rivers. Science of the Total Environment 867, 161537. 36640879 10.1016/j.scitotenv.2023.161537

[brv70058-bib-0444] Soubeyran, Y. , Meyer, J.‐Y. , Lebouvier, M. , De Thoisy, B. , Lavergne, C. , Urtizberea, F. & Kirchner, F. (2015). Dealing with invasive alien species in the French overseas territories: results and benefits of a 7‐year initiative. Biological Invasions 17, 545–554.

[brv70058-bib-0445] Soubeyrand, S. , Estoup, A. , Cruaud, A. , Malembic‐Maher, S. , Meynard, C. , Ravigné, V. , Barbier, M. , Barrès, B. , Berthier, K. , Boitard, S. , Dallot, S. , Gaba, S. , Grosdidier, M. , Hannachi, M. , Jacques, M.‐A. , *et al*. (2024). Building integrated plant health surveillance: a proactive research agenda for anticipating and mitigating disease and pest emergence. CABI Agriculture and Bioscience 5, 72.

[brv70058-bib-0446] Spatz, D. R. , Holmes, N. D. , Will, D. J. , Hein, S. , Carter, Z. T. , Fewster, R. M. , Keitt, B. , Genovesi, P. , Samaniego, A. , Croll, D. A. , Tershy, B. R. & Russell, J. C. (2022). The global contribution of invasive vertebrate eradication as a key Island restoration tool. Scientific Reports 12, 13391. 35948555 10.1038/s41598-022-14982-5 PMC9365850

[brv70058-bib-0447] Stowhas Salinas, P. , Carlton, J. , Thiel, M. , Santibañez, J. , Sáez, R. , Puga, A. , Munizaga, M. & Brante, A. (2023). Marine bioinvasions in Chile: a national research and conservation management agenda. Management of Biological Invasions 14, 595–618.

[brv70058-bib-0448] Stringham, O. C. & Lockwood, J. L. (2018). Pet problems: biological and economic factors that influence the release of alien reptiles and amphibians by pet owners. Journal of Applied Ecology 55, 2632–2640.

[brv70058-bib-0449] Tan, H. H. , Lim, K. K. P. , Liew, J. H. , Low, B. W. , Lim, B. H. R. , Kwik, J. T. B. & Yeo, D. C. J. (2020). The non‐native freshwater fishes of Singapore: an annotated compilation. Raffles Bulletin of Zoology 68, 150–195.

[brv70058-bib-0450] Tedeschi, L. , Biancolini, D. , Capinha, C. , Rondinini, C. & Essl, F. (2022). Introduction, spread, and impacts of invasive alien mammal species in Europe. Mammal Review 52, 252–266. 35875182 10.1111/mam.12277 PMC9299096

[brv70058-bib-0451] Teixeira, L. & Creed, J. (2020). A decade on: an updated assessment of the status of marine non‐indigenous species in Brazil. Aquatic Invasions 15, 30–43.

[brv70058-bib-0452] Telford, N. S. , Channing, A. & Measey, J. (2019). Origin of invasive populations of the guttural toad (*Sclerophrys gutturalis*) on Réunion and Mauritius Islands and in Constantia, South Africa. Herpetological Conservation and Biology 14, 380–392.

[brv70058-bib-0453] Thakur, M. P. , van der Putten, W. H. , Cobben, M. M. P. , van Kleunen, M. & Geisen, S. (2019). Microbial invasions in terrestrial ecosystems. Nature Reviews Microbiology 17, 621–631. 31350537 10.1038/s41579-019-0236-z

[brv70058-bib-0454] Thaman, R. R. (2011). The silent invasion of our islands. Mai Life 55, 64–65.

[brv70058-bib-0455] Thaman, R. R. & O'Brien, K. (2011). Caterpillar devastates kanava and undermines resilience to climate change in Tuvalu. Mai Life 50, 56–57.

[brv70058-bib-0456] Therriault, T. W. , Nelson, J. C. , Carlton, J. T. , Liggan, L. , Otani, M. , Kawai, H. , Scriven, D. , Ruiz, G. M. & Murray, C. C. (2018). The invasion risk of species associated with Japanese tsunami marine debris in Pacific North America and Hawaii. Marine Pollution Bulletin 132, 82–89. 29395102 10.1016/j.marpolbul.2017.12.063

[brv70058-bib-0457] Thines, M. (2011). Recent outbreaks of downy mildew on grape ivy (*Parthenocissus tricuspidata*, Vitaceae) in Germany are caused by a new species of *Plasmopara* . Mycological Progress 10, 415–422.

[brv70058-bib-0458] Thines, M. , Buaya, A. , Ali, T. & Brand, T. (2020). *Peronospora aquilegiicola* made its way to Germany: the start of a new pandemic? Mycological Progress 19, 791–798.

[brv70058-bib-0459] Tokarska‐Guzik, B. (2005). The Establishment and Spread of Alien Plant Species (Kenophytes) in the Flora of Poland. Wydawnictwo Uniwersytetu Śląskiego, Katowice.

[brv70058-bib-0460] Toomes, A. , García‐Díaz, P. , Wittmann, T. A. , Virtue, J. & Cassey, P. (2020). New aliens in Australia: 18 years of vertebrate interceptions. Wildlife Research 47, 55–67.

[brv70058-bib-0461] Toral‐Granda, M. V. , Causton, C. E. , Jäger, H. , Trueman, M. , Izurieta, J. C. , Araujo, E. , Cruz, M. , Zander, K. K. , Izurieta, A. & Garnett, S. T. (2017). Alien species pathways to the Galapagos Islands, Ecuador. PLoS One 12, e0184379. 28902860 10.1371/journal.pone.0184379 PMC5597199

[brv70058-bib-0462] Torres, M. D. L. & Mena, C. F. (eds) (2018). Understanding Invasive Species in the Galapagos Islands: From the Molecular to the Landscape. Springer, Cham.

[brv70058-bib-0463] Trueman, M. , Atkinson, R. , Guézou, A. & Wurm, P. (2010). Residence time and human‐mediated propagule pressure at work in the alien flora of Galapagos. Biological Invasions 12, 3949–3960.

[brv70058-bib-0464] Tsatsia, H. & Jackson, G. (2022). Giant African snail (050). Pacific Pests, Pathogens, Weed & Pesticides – Online edition. https://apps.lucidcentral.org/pppw_v11/text/web_full/entities/giant_african_snail_050.htm.

[brv70058-bib-0465] Ugarte, E. , Fuentes, N. & Klotz, S. (2010). European plant in southern South America. In Unwanted Visitors? In Atlas of Biodiversity Risk (ed. J. Settele ), pp. 148–150. Pensoft Publishers, Sofia and Moscow.

[brv70058-bib-0466] Ulman, A. , Abd Rabou, A. F. N. , Al Mabruk, S. , Bariche, M. , Bilecenoğlu, M. , Demirel, N. , Galil, B. S. , Hüseyinoğlu, M. F. , Jimenez, C. , Hadjioannou, L. , Kosker, A. R. , Peristeraki, P. , Saad, A. , Samaha, Z. , Stoumboudi, M. T. , *et al*. (2024). Assessment of human health impacts from invasive pufferfish (attacks, poisonings and fatalities) across the eastern Mediterranean. Biology 13, 208. 38666820 10.3390/biology13040208 PMC11048499

[brv70058-bib-0467] Van der Burg, W. J. , de Freitas, J. , Debrot, A. O. & Lotz, L. A. P. (2012). Naturalised and Invasive Alien Plant Species in the Caribbean Netherlands: Status, Distribution, Threats, Priorities and Recommendations. Plant Research International report 437. Plant Research International, Wageningen.

[brv70058-bib-0468] Vanderploeg, H. A. , Nalepa, T. F. , Jude, D. J. , Mills, E. L. , Holeck, K. T. , Liebig, J. R. , Grigorovich, I. A. & Ojaveer, H. (2002). Dispersal and emerging ecological impacts of Ponto‐Caspian species in the Laurentian Great Lakes. Canadian Journal of Fisheries and Aquatic Sciences 59, 1209–1228.

[brv70058-bib-0469] Vanjil, G. , Kortz, A. , Lenzner, B. , Chuluunbat, J. , Chuluunbat, S. , Magsar, U. , Tsagaan, K. , Erdenechuluun, M. , Tsogtbayar, D. , Bayarmagnai, D. , Sanjaajav, E. , Batbayar, K. , Essl, F. & Pyšek, P. (2024). Alien flora of Mongolia: species richness, introduction dynamics and spatial patterns. Biological Invasions 26, 2407–2419.

[brv70058-bib-0470] Van Kleunen, M. , Dawson, W. , Essl, F. , Pergl, J. , Winter, M. , Weber, E. , Kreft, H. , Weigelt, P. , Kartesz, J. , Nishino, M. , Antonova, L. A. , Barcelona, J. F. , Cabezas, F. J. , Cárdenas, D. , Cárdenas‐Toro, J. , *et al*. (2015). Global exchange and accumulation of non‐native plants. Nature 525, 100–103. 26287466 10.1038/nature14910

[brv70058-bib-0471] Van Kleunen, M. , Pyšek, P. , Dawson, W. , Essl, F. , Kreft, H. , Pergl, J. , Weigelt, P. , Stein, A. , Dullinger, S. , König, C. , Lenzner, B. , Maurel, N. , Moser, D. , Seebens, H. , Kartesz, J. , *et al*. (2019). The global naturalized alien Flora (GloNAF) database. Ecology 100, e02542. 30341991 10.1002/ecy.2542

[brv70058-bib-0472] Vanneste, J. (2008). Erwinia amylovora (fireblight). CABI Compendium , CAB International, Wallingford. 21908. 10.1079/cabicompendium.21908.

[brv70058-bib-0473] Van Wilgen, B. W. , Measey, J. , Richardson, D. M. , Wilson, J. R. & Zengeya, T. A. (eds) (2020). Biological Invasions in South Africa. Springer, Cham.

[brv70058-bib-0474] Vellinga, E. C. , Wolfe, B. E. & Pringle, A. (2009). Global patterns of ectomycorrhizal introductions. New Phytologist 181, 960–973. 19170899 10.1111/j.1469-8137.2008.02728.x

[brv70058-bib-0475] Villaseñor‐Parada, C. , Pauchard, A. & Macaya, E. C. (2017). Ecología de invasiones marinas en Chile continental: ¿qué sabemos y que nos falta por saber? Revista de Biología Marina y Oceanografía 52, 1–17.

[brv70058-bib-0476] Vinogradov, Y. K. & Kupriyanov, A. N. (2016). The Black Book of the Flora of Siberia. Academic publishing house ‘Geo’, Novosibirsk.

[brv70058-bib-0477] Vitule, J. R. S. , Occhi, T. V. T. , Carneiro, L. , Daga, V. S. , Frehse, F. A. , Bezerra, L. A. V. , Forneck, S. , de Pereira, H. S. , Freitas, M. O. , Hegel, C. G. Z. , Abilhoa, V. , Grombone‐Guaratini, M. T. , Queiroz‐Sousa, J. , Pivello, V. R. , Silva‐Matos, D. M. , *et al*. (2021). Non‐native species introductions, invasions, and biotic homogenization in the Atlantic forest. In The Atlantic Forest (eds M. C. M. Marques and C. E. V. Grelle ), pp. 269–295. Springer, Cham.

[brv70058-bib-0478] Vitule, J. R. S. , Occhi, T. V. T. , Kang, B. , Matsuzaki, S. I. , Bezerra, L. A. , Daga, V. S. , Faria, L. , Frehse, F. A. , Walter, F. & Padial, A. A. (2019). Intra‐country introductions unraveling global hotspots of alien fish species. Biodiversity and Conservation 28, 3037–3043.

[brv70058-bib-0479] Voglmayr, H. , Montes‐Borrego, M. & Landa, B. B. (2014). Disentangling *Peronospora* on *Papaver*: phylogenetics, taxonomy, nomenclature and host range of downy mildew of opium poppy (*Papaver somniferum*) and related species. PLoS One 9, e96838. 24806292 10.1371/journal.pone.0096838 PMC4013089

[brv70058-bib-0480] Voglmayr, H. , Schertler, A. , Essl, F. & Krisai‐Greilhuber, I. (2023). Alien and cryptogenic fungi and oomycetes in Austria: an annotated checklist (2nd edition). Biological Invasions 25, 27–38. 36643959 10.1007/s10530-022-02896-2 PMC9832105

[brv70058-bib-0481] Waage, J. K. , Woodhall, J. W. , Bishop, S. J. , Smith, J. J. , Jones, D. R. & Spence, N. J. (2008). Patterns of plant pest introductions in Europe and Africa. Agricultural Systems 99, 1–5.

[brv70058-bib-0482] Walsh, N. G. & Stajsic, V. (eds) (2007). A Census of the Vascular Plants of Victoria, Eightth Edition. National Herbarium of Victoria, Royal Botanic Gardens Melbourne, Melbourne.

[brv70058-bib-0483] Walther, G.‐R. , Roques, A. , Hulme, P. E. , Sykes, M. T. , Pyšek, P. , Kühn, I. , Zobel, M. , Bacher, S. , Botta‐Dukát, Z. , Bugmann, H. , Czúcz, B. , Dauber, J. , Hickler, T. , Jarošík, V. , Kenis, M. , *et al*. (2009). Alien species in a warmer world: risks and opportunities. Trends in Ecology & Evolution 24, 686–693. 19712994 10.1016/j.tree.2009.06.008

[brv70058-bib-0484] Wan, J.‐Z. , Wang, C.‐J. & Yu, F.‐H. (2016). Risk hotspots for terrestrial plant invaders under climate change at the global scale. Environmental Earth Sciences 75, 1012.

[brv70058-bib-0485] Wang, C. , Hawthorne, D. , Qin, Y. , Pan, X. , Li, Z. & Zhu, S. (2017). Impact of climate and host availability on future distribution of Colorado potato beetle. Scientific Reports 7, 4489. 28674384 10.1038/s41598-017-04607-7 PMC5495769

[brv70058-bib-0486] Wasowicz, P. , Przedpelska‐Wasowicz, E. M. & Kristinsson, H. (2013). Alien vascular plants in Iceland: diversity, spatial patterns, temporal trends, and the impact of climate change. Flora – Morphology, Distribution, Functional Ecology of Plants 208, 648–673.

[brv70058-bib-0487] Weyl, O. L. F. , Ellender, B. R. , Wassermann, R. J. , Truter, M. , Dalu, T. , Zengeya, T. A. & Smit, N. J. (2020). Alien freshwater fauna in South Africa. In Biological Invasions in South Africa (eds B. W. van Wilgen , J. Measey , D. M. Richardson , J. R. Wilson and T. A. Zengeya ), pp. 153–183. Springer, Cham.

[brv70058-bib-0488] Wijesundara, S. (2010). Invasive alien plants in Sri Lanka. In Invasive Alien Species – Strengthening Capacity to Control Introduction and Spread in Sri Lanka (eds B. Marambe , P. Silva , S. Wijesundara and N. Atapattu ), pp. 27–38. Biodiversity Secretariat of the Ministry of Environment, Sri Lanka.

[brv70058-bib-0489] Willette, D. A. , Chalifour, J. , Debrot, A. O. D. , Engel, M. S. , Miller, J. , Oxenford, H. A. , Short, F. T. , Steiner, S. C. C. & Védie, F. (2014). Continued expansion of the trans‐Atlantic invasive marine angiosperm *Halophila stipulacea* in the eastern Caribbean. Aquatic Botany 112, 98–102.

[brv70058-bib-0490] Williams, S. L. (2007). Introduced species in seagrass ecosystems: status and concerns. Journal of Experimental Marine Biology and Ecology 350, 89–110.

[brv70058-bib-0491] Wilson, J. R. U. , Datta, A. , Hirsch, H. , Keet, J.‐H. , Mbobo, T. , Nkuna, K. V. , Nsikani, M. M. , Pyšek, P. , Richardson, D. M. , Zengeya, T. A. & Kumschick, S. (2020). Is invasion science moving towards agreed standards? The influence of selected frameworks. NeoBiota 62, 569–590.

[brv70058-bib-0492] Winters, G. , Beer, S. , Willette, D. A. , Viana, I. G. , Chiquillo, K. L. , Beca‐Carretero, P. , Villamayor, B. , Azcárate‐García, T. , Shem‐Tov, R. , Mwabvu, B. , Migliore, L. , Rotini, A. , Oscar, M. A. , Belmaker, J. , Gamliel, I. , *et al*. (2020). The tropical seagrass *Halophila stipulacea*: reviewing what we know from its native and invasive habitats, alongside identifying knowledge gaps. Frontiers in Marine Science 7, 300.

[brv70058-bib-0493] Wood, A. R. (2017). Fungi and invasions in South Africa. Bothalia 47, a2124.

[brv70058-bib-0494] Wu, S.‐H. , Yang, T. Y. A. , Teng, Y. , Chang, C.‐Y. , Yang, K. & Hsieh, C.‐F. (2010). Insights of the latest naturalized flora of Taiwan: change in the past eight years. Taiwania 55, 139–159.

[brv70058-bib-0495] Xiong, W. , Sui, X. , Liang, S.‐H. & Chen, Y. (2015). Non‐native freshwater fish species in China. Reviews in Fish Biology and Fisheries 25, 651–687.

[brv70058-bib-0496] Xu, H. , Qiang, S. , Genovesi, P. , Ding, H. , Wu, J. , Meng, L. , Han, Z. , Miao, J. , Hu, B. , Guo, J. , Sun, H. , Huang, C. , Lei, J. , Le, Z. , Zhang, X. , *et al*. (2012). An inventory of invasive alien species in China. NeoBiota 15, 1–26.

[brv70058-bib-0497] Xu, H. G. & Qiang, S. (2018). China's Invasive Alien Species. Science Press, Beijing.

[brv70058-bib-0498] Yamanaka, T. , Morimoto, N. , Nishida, G. M. , Kiritani, K. , Moriya, S. & Liebhold, A. M. (2015). Comparison of insect invasions in North America, Japan and their islands. Biological Invasions 17, 3049–3061.

[brv70058-bib-0499] Yoshida, K. , Schuenemann, V. J. , Cano, L. M. , Pais, M. , Mishra, B. , Sharma, R. , Lanz, C. , Martin, F. N. , Kamoun, S. , Krause, J. , Thines, M. , Weigel, D. & Burbano, H. A. (2013). The rise and fall of the *Phytophthora infestans* lineage that triggered the Irish potato famine. eLife 2, e00731. 23741619 10.7554/eLife.00731 PMC3667578

[brv70058-bib-0500] Yuldashov, M. A. (2018). Introduction of alien fish species to waterbodies of Uzbekistan. International Journal of Science and Research 7, 1213–1219.

[brv70058-bib-0501] Yuma, M. , Hosoya, K. & Nagata, Y. (1998). Distribution of the freshwater fishes of Japan: an historical overview. Environmental Biology of Fishes 52, 97–124.

[brv70058-bib-0502] Zalba, S. M. , Sanhueza, C. , Cuevas, Y. , Wong, L. J. & Pagad, S. (2021). *Global register of introduced and invasive species – Argentina. Version 1.6*. Invasive species specialist group ISSG. 10.15468/qr5pjs.

[brv70058-bib-0503] Zenetos, A. , Tsiamis, K. , Galanidi, M. , Carvalho, N. , Bartilotti, C. , Canning‐Clode, J. , Castriota, L. , Chainho, P. , Comas‐González, R. , Costa, A. C. , Dragičević, B. , Dulčić, J. , Faasse, M. , Florin, A.‐B. , Gittenberger, A. , *et al*. (2022). Status and trends in the rate of introduction of marine non‐indigenous species in European seas. Diversity 14, 1077.

[brv70058-bib-0504] Zug, G. R. (2013). Reptiles and Amphibians of the Pacific Islands: A Comprehensive Guide. University of California Press, Berkeley.

[brv70058-bib-0505] Zvyagintsev, A. Y. , Radashevsky, V. I. , Ivin, V. V. , Kashin, I. A. & Gorodkov, A. N. (2011). Nonindigenous species in the far eastern seas of Russia. Russian Journal of Biological Invasions 2, 164–182.

